# The substrate‐dependent regulatory effects of the AfeI/R system in *Acidithiobacillus ferrooxidans* reveals the novel regulation strategy of quorum sensing in acidophiles

**DOI:** 10.1111/1462-2920.15163

**Published:** 2020-08-02

**Authors:** Xue‐Yan Gao, Chang‐Ai Fu, Likai Hao, Xiu‐Feng Gu, Rui Wang, Jian‐Qiang Lin, Xiang‐Mei Liu, Xin Pang, Cheng‐Jia Zhang, Jian‐Qun Lin, Lin‐Xu Chen

**Affiliations:** ^1^ State Key Laboratory of Microbial Technology Shandong University, No. 72 Binhai Road Qingdao 266237 China; ^2^ State Key Laboratory of Environmental Geochemistry Institute of Geochemistry, Chinese Academy of Sciences No. 99 Lincheng West Road, Guiyang 550081 China; ^3^ CAS Center for Excellence in Quaternary Science and Global Change Xi'an 710061 China

## Abstract

A LuxI/R‐like quorum sensing (QS) system (AfeI/R) has been reported in the acidophilic and chemoautotrophic *Acidithiobacillus* spp. However, the function of AfeI/R remains unclear because of the difficulties in the genetic manipulation of these bacteria. Here, we constructed different *afeI* mutants of the sulfur‐ and iron‐oxidizer *A*. *ferrooxidans*, identified the N‐acyl homoserine lactones (acyl‐HSLs) synthesized by AfeI, and determined the regulatory effects of AfeI/R on genes expression, extracellular polymeric substance synthesis, energy metabolism, cell growth and population density of *A*. *ferrooxidans* in different energy substrates. Acyl‐HSLs‐mediated distinct regulation strategies were employed to influence bacterial metabolism and cell growth of *A*. *ferrooxidans* cultivated in either sulfur or ferrous iron. Based on these findings, an energy‐substrate‐dependent regulation mode of AfeI/R in *A*. *ferrooxidans* was illuminated that AfeI/R could produce different types of acyl‐HSLs and employ specific acyl‐HSLs to regulate specific genes in response to different energy substrates. The discovery of the AfeI/R‐mediated substrate‐dependent regulatory mode expands our knowledge on the function of QS system in the chemoautotrophic sulfur‐ and ferrous iron‐oxidizing bacteria, and provides new insights in understanding energy metabolism modulation, population control, bacteria‐driven bioleaching process, and the coevolution between the acidophiles and their acidic habitats.

## Introduction

Acidophiles, a class of important extremophiles and geo‐microbes, are widely distributed in the hot springs and acid mines. In these natural habitats, the chemoautotrophic acidophiles participate in the global element cycles of sulfur and iron via the oxidation of reduced inorganic sulfur compounds (RISCs) to sulfate and the conversions between ferrous and ferric ions (Menzel *et al*., 2015; Quatrini and Johnson, 2016). The acidophile‐driven bioleaching process has given rise to a worldwide problem of water and solid contaminations in natural and man‐made mine environments (Chen *et al*., 2014). On the other hand, this process has been advantageously utilized in the biomining industry for the recovery of valuable metals from sulfide ores, such as copper or gold (Rawlings, 1998; Chen *et al*., 2014). Thus, the researches on the bioleaching microbes and their metabolism and regulation mechanisms are of significance for the treatments of acid mine contaminations and the development of high‐efficient biological metallurgy technology. *Acidithiobacillus* spp., a group of acidophilic chemolithoautotrophic Gram‐negative bacteria, are prevalent in the sulfur‐ and ferrous iron‐ contained acidic ecosystems, and are the predominant player in acidophile communities structures in acid mine drainages (AMD) and terrestrial hot springs (Rawlings, 1998; Liljeqvist *et al*., 2015; Quatrini and Johnson, 2018). They are the most active and wide‐used bioleaching bacteria in the biomining industry (Olson *et al*., 2003; Rohwerder *et al*., 2003). All *Acidithiobacillus* strains are capable of oxidizing various RISCs for autotrophic growth, and some of them can use ferrous iron as an energy substrate (Bosecker, 1997; Rohwerder *et al*., 2003). Seven species have been identified in the *Acidithiobacillus* genus, including four sulfur‐ and ferrous iron‐oxidizing species (*A*. *ferrooxidans*, *A*. *ferridurans*, *A*. *ferriphilus* and *A*. *ferrivorans*), and three sulfur‐oxidizing‐only species (*A*. *thiooxidans*, *A*. *caldus* and *A*. *albertensis*).

*Acidithiobacillus*. *ferrooxidans* has become an important model bacterium for the researches of the acidophilic bacteria on physiological biochemistry, molecular biology, microbial mineralogy and so on (Sugio *et al*., 1987; Rawlings, 2002). It can gain energy by the oxidation of ferrous iron and reduced sulfur compounds at the aerobic condition, and can also obtain energy via anaerobic metabolisms including the oxidation of sulfur and hydrogen by using ferric iron as an electron acceptor and the oxidation of hydrogen by using sulfur as electron acceptor (Ohmura *et al*., 2002). For the variety of RISCs, the sulfur metabolism is achieved by different kinds of enzymes located in different cellular compartments in *A*. *ferrooxidans*, such as thiosulfate dehydrogenase, thiosulfate quinone oxidoreductase, tetrathionate hydrolase (TetH) in periplasmic space; persulfide dioxygenase (formerly named as sulfur dioxygenase, SDO), HDR, Hdr‐like complex in the cytoplasm; and sulfide:quinone oxidoreductase (SQR) located in the inner membrane (Ng *et al*., 2000; Sugio *et al*., 2009; Wang *et al*., 2019). Ferrous iron oxidation in *A*. *ferrooxidans* involves the *petI* and *rus* operons, two transcriptional units that mediate downhill and uphill electron pathways to generate ATP and NADH respectively (Quatrini *et al*., 2009). Therefore, *A*. *ferrooxidans* exhibits distinct physiological characteristics and gene expression profiles depending on the availability of these two energy substrates.

Quorum‐sensing (QS) is a cell‐to‐cell communication mechanism that enables bacteria to control gene expression in response to changes in cell density (Parsek and Greenberg, 2000; Wackett, 2008). QS regulation depends on the production, release, accumulation and detection of signaling autoinducers, and this process is generally mediated by the autoinducer synthase and cognate autoinducer receptor (An *et al*., 2006; Schaefer *et al*., 2008). QS has been widely identified in Gram‐negative and Gram‐positive bacteria and is fundamental for cell‐to‐cell communication (Juhas *et al*., 2005; Kai and Bassler, 2016). Hundreds of traits can be regulated via QS in both pathogenic and environmental bacteria, such as EPS synthesis, biofilm formation, cell colonization, bioluminescence and the secretion of virulence factors (Goo *et al*., 2015; Ben‐Yaakov and Salomon, 2019). QS can also regulate metabolic processes in some bacteria, such as sugar and phosphate metabolism, as well as secondary metabolites (Goo *et al*., 2015; Certner and Vollmer, 2018; Ha *et al*., 2018). Moreover, QS has been also observed in many extremophiles; however, such studies are limited by the difficulty of genetic manipulation in these bacteria (Inaba *et al*., 2018).

A LuxI/R‐like QS system (AfeI/R), encoded by the *afeI‐orf3‐afeR* operon, was discovered in *A*. *ferrooxidans* (Farah *et al*., 2005; Rivas *et al*., 2005). Similar to the prototypical LuxI/R‐like system in many Gram‐negative bacteria, the QS system in *A*. *ferrooxidans* consists of a LuxI‐type autoinducer synthase (AfeI) and a LuxR‐type receptor (AfeR) that mediates the production of N‐acyl homoserine lactones (acyl‐HSLs) and controls genes expression by binding signaling molecules respectively (An *et al*., 2006; Schaefer *et al*., 2008). Additionally, another potential acyl‐HSL synthetase (Act) in *A*. *ferrooxidans* was discovered in an operon. This operon encompasses four co‐transcribed genes (*glyQ*, *glysS*, *gph* and *act*), which encode for the α and β subunits of glycine tRNA synthetase, a phosphatase and an acyltransferase respectively (Rivas *et al*., 2007). Given that Act has only been previously confirmed to produce C_14_‐HSL in *Escherichia coli* in the absence of a corresponding signal molecule receptor gene in the operon, the role of the Act‐like QS system in *A*. *ferrooxidans* remains largely unclear (Rivas *et al*., 2007; Gonzalez *et al*., 2013).

Nine acyl‐HSLs have been identified from *A*. *ferrooxidans* cultures, including C_12_‐HSL, C_14_‐HSL, 3‐OH‐C_8_‐HSL, 3‐OH‐C_10_‐HSL, 3‐OH‐C_12_‐HSL, 3‐OH‐C_14_‐HSL, 3‐OH‐C_16_‐HSL, 3‐O‐C_12_‐HSL and 3‐O‐C_14_‐HSL (Farah *et al*., 2005). The addition of exogenous acyl‐HSLs led to notable phenotypic changes. For instance, the addition of C_12_/C_14_‐HSLs mixtures or acyl‐HSL analogs promoted biofilm formation on the surfaces of elemental sulfur and pyrite (Gonzalez *et al*., 2013; Bellenberg *et al*., 2014). Moreover, the addition of a C_14_‐HSLs mixture also improved *A*. *ferrooxidans* electroactivity on an inert carbon electrode (Chabert *et al*., 2017). Furthermore, more than 100 genes were differentially expressed in *A*. *ferrooxidans* exposed to a tetrazolic acyl‐HSL analog (tetrazole 9c), of which 60 were involved in biofilm formation (Mamani *et al*., 2016). Exposure to a synthetic QS blocker also revealed that AfeI/R mediates Cu^2+^ resistance in *A*. *ferrooxidans* (Wenbin *et al*., 2011). In addition, the overexpression of *afeI/R* operon suggested the important roles of Afe/R in the growth of *A*. *ferrooxidans* in S^0^‐enriched media and in improving the bioleaching efficiency of *A*. *ferrooxidans* to ores (Gao *et al*., 2020). However, although some functions of AfeI/R have been identified via the addition assays of exogenous signal molecules, the understanding of the roles of AfeI/R in *A*. *ferrooxidans* has not been fully achieved. For example, the acyl‐HSLs synthesized by AfeI were not determined due to the interference of the Act system. Meanwhile, the roles of AfeI‐produced acyl‐HSLs were also unclear.

The question that whether AfeI/R has a regulatory function in Fe^2+^‐cultivating *A*. *ferrooxidans* remains to be answered. Unlike elemental sulfur, ferrous iron exists as an ion in bacterial cultures (Quatrini *et al*., 2009). The utilization of elemental sulfur by *A*. *ferrooxidans* requires EPS‐mediated attachment, while ferrous iron metabolism employs very different pathways (Gehrke *et al*., 1998; Harneit *et al*., 2006; Quatrini *et al*., 2009). Notably, a lower transcriptional level of the *afe*I/R operon has been observed in Fe^2+^‐enriched media compared with S^0^‐enriched media (Farah *et al*., 2005). However, the role of AfeI/R in *A*. *ferrooxidans* when Fe^2+^ is used as the energy source is not clear.

Mutagenesis of the QS genes has become a powerful and effective approach to study the biological functions and regulation mechanisms of QS in many bacteria. In this study, we explored the distribution of AfeI/R‐like QS system in *Acidithiobacillus* and other acidophiles. Several mutants of the acyl‐HSLs synthetase genes were successfully constructed and used to study the effect of gene knockout and overexpression on acyl‐HSLs synthesize, energy metabolism, cell growth, EPS secretion and gene transcript profile in *A*. *ferrooxidans* cultivated with different energy substrates. Moreover, the acyl‐HSLs produced by AfeI were identified, and two key acyl‐HSLs were found to influence *A*. *ferrooxidans* growth. Therefore, our results revealed that AfeI/R‐mediated regulation effects in *A*. *ferrooxidans* were versatile and substrate‐dependent. The results in this study provide new insights in understanding the QS‐mediated regulation in acidophilic sulfur‐oxidizing and/or ferrous iron‐oxidizing bacteria.

## Results

### Distribution of AfeI/R‐like QS system in *Acidithiobacillus* and other acidophiles

**Fig 1:**
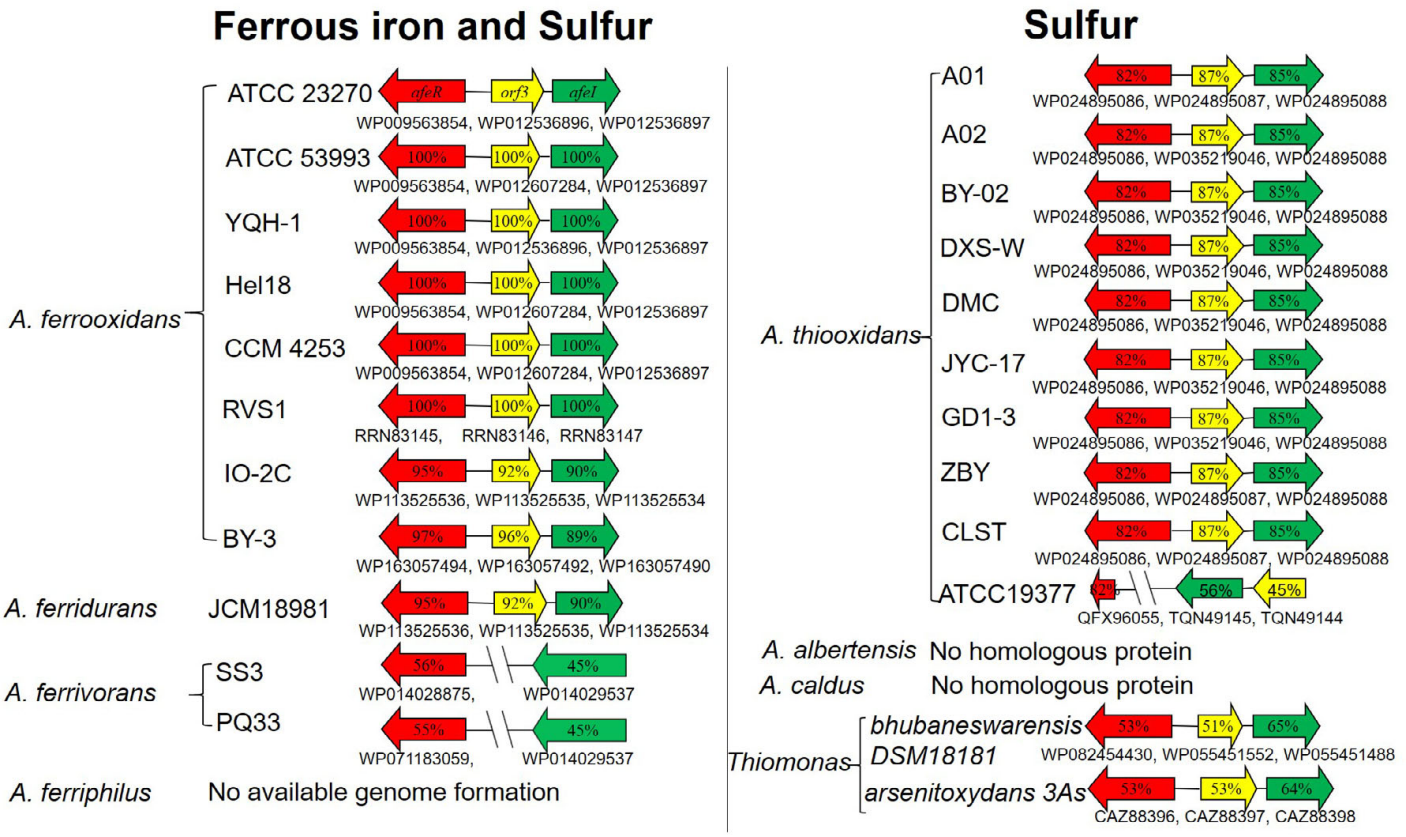
Distribution of AfeI/R‐like QS system in *Acidithiobacillus* and other acidophiles. [Color figure can be viewed at wileyonlinelibrary.com]

The protein sequences of AfeI, AfeR and Orf3 from *A*. *ferrooxidans* ATCC 23270 were used to explore the homologous proteins in *Acidithiobacillus* and other acidophiles based on the reported acidophilic species and their published genome sequences on the NCBI database (Quatrini and Johnson, 2016). As shown in Fig. 1, AfeI/R‐like QS system could be identified from *A*. *ferrooxidans*, *A*. *ferridurans*, *A*. *ferrivorans* and *A*. *thiooxidans* in the genus of *Acidithiobacillus*. AfeI/R system was found in all the sulfur‐ and ferrous iron‐oxidizing species except *A*. *ferriphilus* that did not have the published genomic information, while only *A*. *thiooxidans* possesses the QS system in the three reported sulfur‐oxidizing‐only species of *Acidithiobacillus*. The result indicated the distribution of AfeI/R in the sulfur‐ and ferrous iron‐oxidizing species is more pervasive than that in sulfur‐oxidizing‐only species of *Acidithiobacillus*. AfeI/R‐like QS system also found in the genus of *Thiomonas* that is a group of chemoautotrophic sulfur‐oxidizing‐only bacteria. AfeI/R system showed some variations at the gene arrangement and protein sequence in *Acidithiobacillus*. The *afeR‐orf3‐afeI* operon could be identified from the species of *A*. *ferrooxidans*, *A*. *ferridurans* and *A*. *thiooxidans*, while *A*. *ferrivorans* only possesses two separated genes *afeI* and *afeR* encoding the proteins with low identities to that from *A*. *ferrooxidans* ATCC 23270. Although almost all of the strains in *A*. *thiooxidans* have the conserved *afeI/R* operon, *afeR* gene and the *afeI‐orf3* are separated on the genome of *A*. *thiooxidans* ATCC 19377. A truncated AfeR and the low‐identity AfeI and orf3 are found in *A*. *thiooxidans* ATCC 19377, in contrast with those in other *A*. *thiooxidans* strains. Therefore, the gene arrangement and protein sequence of AfeI/R system in *Acidithiobacillus* would be variant in different species or strains.

### Generation of *A*. *ferrooxidans* *afeI* mutants

**Fig 2:**
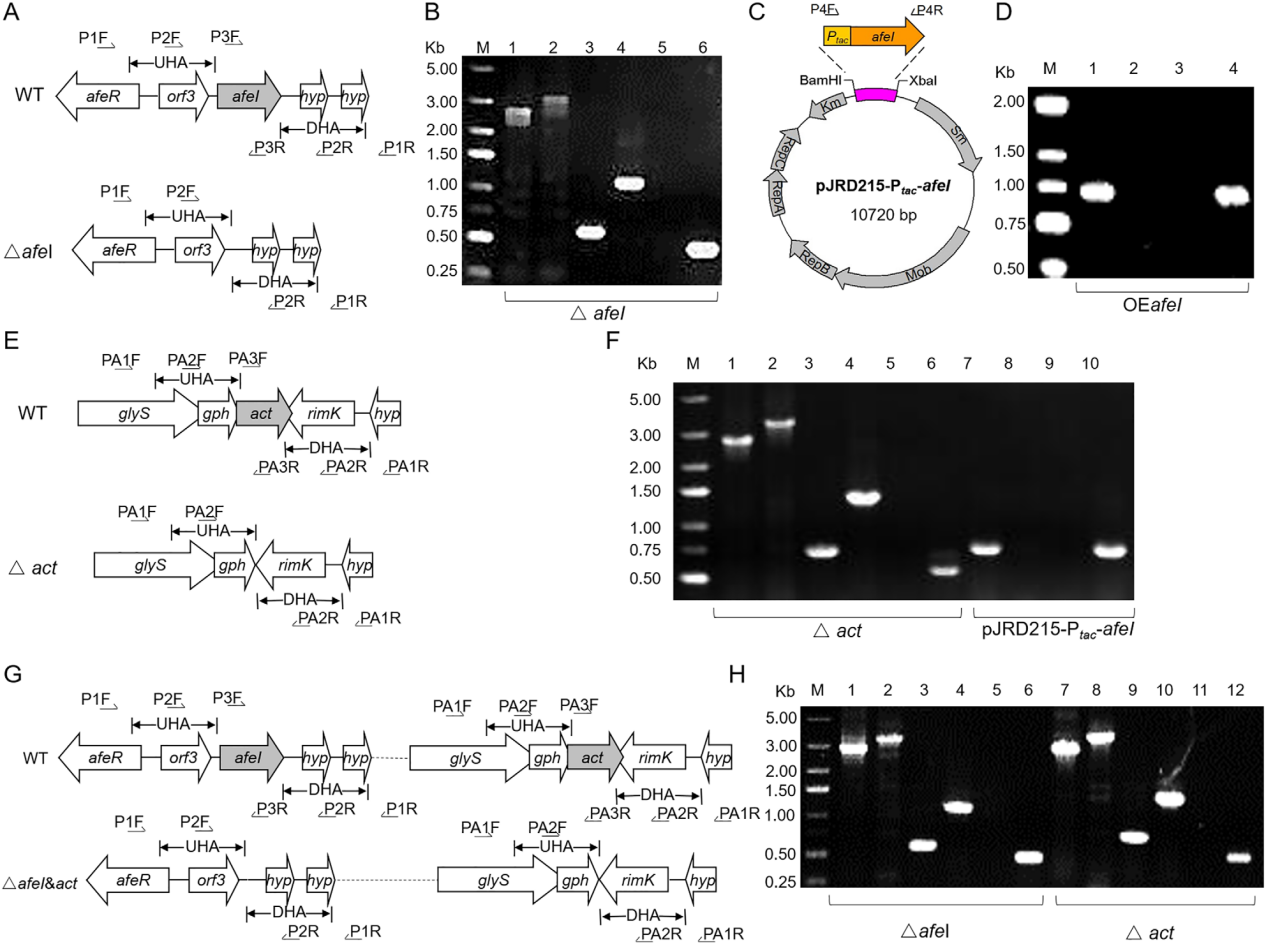
Verification of different types of engineered bacteria during the generation of the *afeI* deletion and overexpression strains. A. Diagram of the *afeI* gene cluster and verification primers. UHA and DHA represent upstream and downstream homologous arms respectively. B. Electrophoretic analysis of PCR products to verify Δ*afeI*. Lanes 1, 3 and 5, PCR products from Δ*afeI* using the primer pair P1F/R, P2F/R and P3F/R respectively; lanes 2, 4 and 6, PCR products from wild type using the primer pair P1F/R, P2F/R and P3F/R respectively. C. Illustration of the *afeI* expression vector pJRD215‐P_*tac*_‐*afeI* and verification primers. D. Electrophoretic analysis of PCR products to verify the *afeI*‐overexpression strains. Lanes 1, 2, 3 and 4, PCR products from the *afeI* overexpression strain, the wild‐type strain, pJRD215 and pJRD215‐P_*tac*_‐*afeI* respectively, using the primer pair P4F/R. E. Diagram of the *act* gene cluster and verification primers. F. Electrophoretic analysis of PCR products to verify Δ*act(afeI)*. Lanes 1, 3 and 5, PCR products from Δ*act(afeI)* using the primer pair PA1F/R, PA2F/R and PA3F/R respectively; lanes 2, 4 and 6, PCR products from wild type using the primer pair PA1F/R, PA2F/R and PA3F/R respectively; lanes 7, 8, 9 and 10, PCR products from the Δ*act(afeI)* strain, the wild‐type strain, pJRD215 and pJRD215‐P_*tac*_‐*afeI* respectively, using the primer pair P4F/R. G. Diagram of the *ΔafeI&act* gene cluster and verification primers. H. Electrophoretic analysis of PCR products to verify *ΔafeI&act*. Lanes 1, 3, 5, 7, 9 and 11, PCR products from *ΔafeI&act* using the primer pair P1F/R, P2F/R, P3F/R, PA1F/R, PA2F/R and PA3F/R respectively; lanes 2, 4, 6, 8, 10 and 12, PCR products from wild type using the primer pair P1F/R, P2F/R, P3F/R, PA1F/R, PA2F/R and PA3F/R respectively. [Color figure can be viewed at wileyonlinelibrary.com]

To characterize the biological function of the AfeI/R QS system, the acyl‐HSL synthase gene (*afeI*, AFE_1999) was targeted to generate *afeI* deletion and overexpression mutant strains. The *afeI* knockout strains were screened and identified by PCR using different primer sets (Fig. 2A and B). The Δ*afeI* strain was a markerless in‐frame AfeI mutation with a 552‐bp deletion from the start site (ATG) to the stop codon (TAA). The *afeI* expression plasmid was constructed using a *tac* promoter to initiate gene transcription, as well as the autonomously replicating plasmid pJRD215 as the backbone of the expression plasmid (Fig. 2C). The constructed *afeI* expression plasmid and the backbone plasmid were respectively conjugated into wild type *A*. *ferrooxidans* strain WT, generating the *afeI* overexpression strain OE*afeI* and the wild‐type control strain WT(pJRD215) (Fig. 2D).

To identify the molecules synthesized by AfeI, a 639‐bp sequence of the acyltransferase gene (*act*) was deleted from strains of WT and Δ*afeI*, generating Δ*act* and Δ*afeI&act* respectively (Fig. 2E and G). Furthermore, the *afeI* expression plasmid (pJRD215‐P_*tac*_‐*afeI*) and the empty plasmid (pJRD215) were conjugated into Δ*act* and Δ*afeI&act*, resulting in the *afeI*‐expression‐only strain Δ*act*(*afeI*) and the *afeI* and *act* deletion strain Δ*afeI&act*(215) respectively.

### Substrate‐dependent regulatory effects of the AfeI/R on *A*. *ferrooxidans* growth and energy metabolism

**Fig 3:**
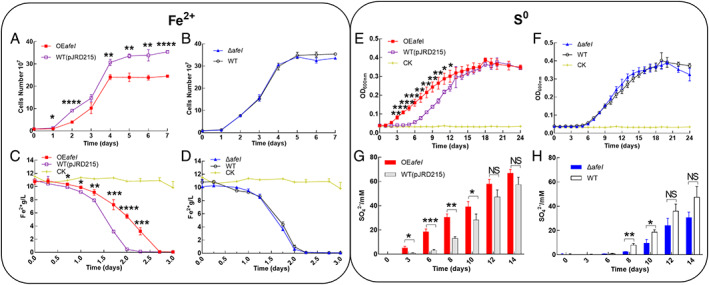
Analyses of the growth and metabolism of engineered *A*. *ferrooxidans* strains. Growth (A, B) and ferrous oxidation (C, D) of the *afeI* overexpression (OE*afeI*) and knockout (Δ*afeI*) strains in Fe^2+^‐enriched media. Growth (E, F) and sulfate production (G, H) curves for the *afeI* overexpression (OE*afeI*) and knockout (Δ*afeI*) strains S^0^‐enriched media. CK indicates control. NS indicates no significant difference. [Color figure can be viewed at wileyonlinelibrary.com]

When cells were grown in Fe^2+^‐enriched media, the cell growth rate, maximum cell density and ferrous iron oxidation rate of the *afeI* overexpression strain were dramatically decreased, and the maximum cell density of the *afeI* overexpression strain reached only approximately 70% of that of the control strain (Fig. 3A and C). In contrast, almost no difference was observed between the *afeI* knockout and the WT strain in terms of cell growth and ferrous iron oxidation (Fig. 3B and D). These results indicate that overexpression of *afeI* could inhibit *A*. *ferrooxidans* ferrous iron oxidation and cell population in Fe^2+^‐enriched media.

When elemental sulfur was used as an energy substrate, overexpression of *afeI* significantly increased the cell density in the lag and exponential growth phases of the *afeI* overexpression strain compared with the wild‐type control strain WT (pJRD215) (Fig. 3E), and simultaneously increased sulfate production on days 3, 6, 8 and 10 (Fig. 3G). However, the enhanced growth caused by *afeI* overexpression decreased and ultimately disappeared when the cells entered the late exponential and stationary growth phases (Fig. 3E and G). Deletion of *afeI* did not distinctly affect cell density, although a slight decrease in sulfate production was observed on days 8 and 10 for the Δ*afeI* mutant (Fig. 3F and H). These results indicated that a high level of AfeI expression could enhance sulfur metabolism and cell growth in *A*. *ferrooxidans*, and this AfeI/R‐mediated regulation was dependent on bacterial growth stage in the S^0^‐enriched media.

### Substrate‐dependent influences of AfeI/R on EPS synthesis in *A*. *ferrooxidans*

**Fig 4:**
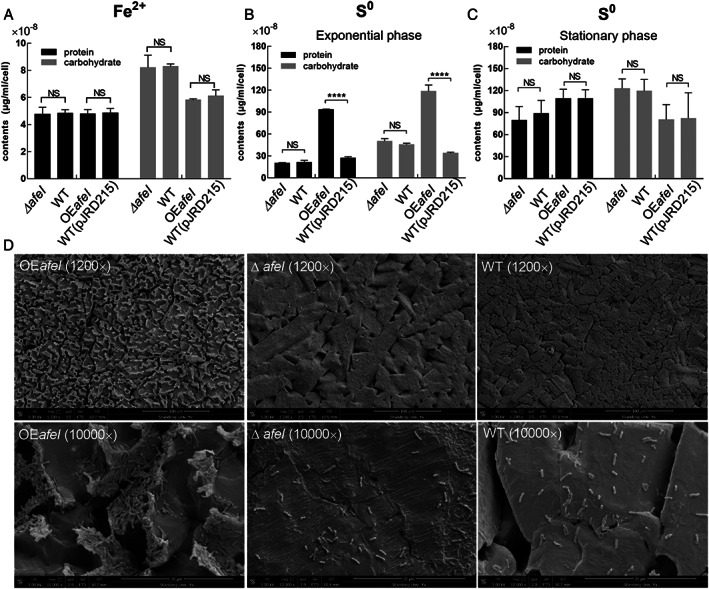
Analysis of EPS synthesis and cell attachment. The protein and carbohydrate levels of EPS in Fe^2+^‐enriched media (A) and S^0^‐enriched media (B and C). Observation of cell attachment on sulfur coupons by SEM (D). OE*afeI* indicates *afeI* overexpression strains.

In Fe^2+^‐enriched media, the main EPS components (proteins and carbohydrates) of *afeI* knockout and overexpression strains had no significant difference compared with that of the control strains (Fig. 4A). The results indicated that the regulatory effect of AfeI/R on *A*. *ferrooxidans* EPS synthesis did not occur in the Fe^2+^‐enriched media.

In S^0^‐enriched media, the levels of the EPS components of the *afeI* overexpression strain were more than threefold higher than those of the control strain in the exponential growth phase (Fig. 4B), whereas the difference disappeared in the stationary phase (Fig. 4C). The deletion of *afeI* did not result in a significant change in EPS protein and carbohydrate content in S^0^‐enriched media (Fig. 4B and C). Scanning electron microscopy (SEM) results showed that the surfaces of sulfur coupons cultivated with the *afeI* overexpression strain were uneven, bumpy and gully‐like, and the cells tended to aggregate and form biofilms (Fig. 4D). In contrast, the results for the Δ*afeI* and WT strains were considerably different, with smooth‐surface sulfur coupons and scattered cells (Fig. 4D).

These results suggested that the regulation of AfeI/R on the EPS synthesize was dependent on the energy substrates.

### Substrate‐dependent regulatory role of acyl‐HSLs synthesized by AfeI on the growth of *A*. *ferrooxidans*

**Fig 5:**
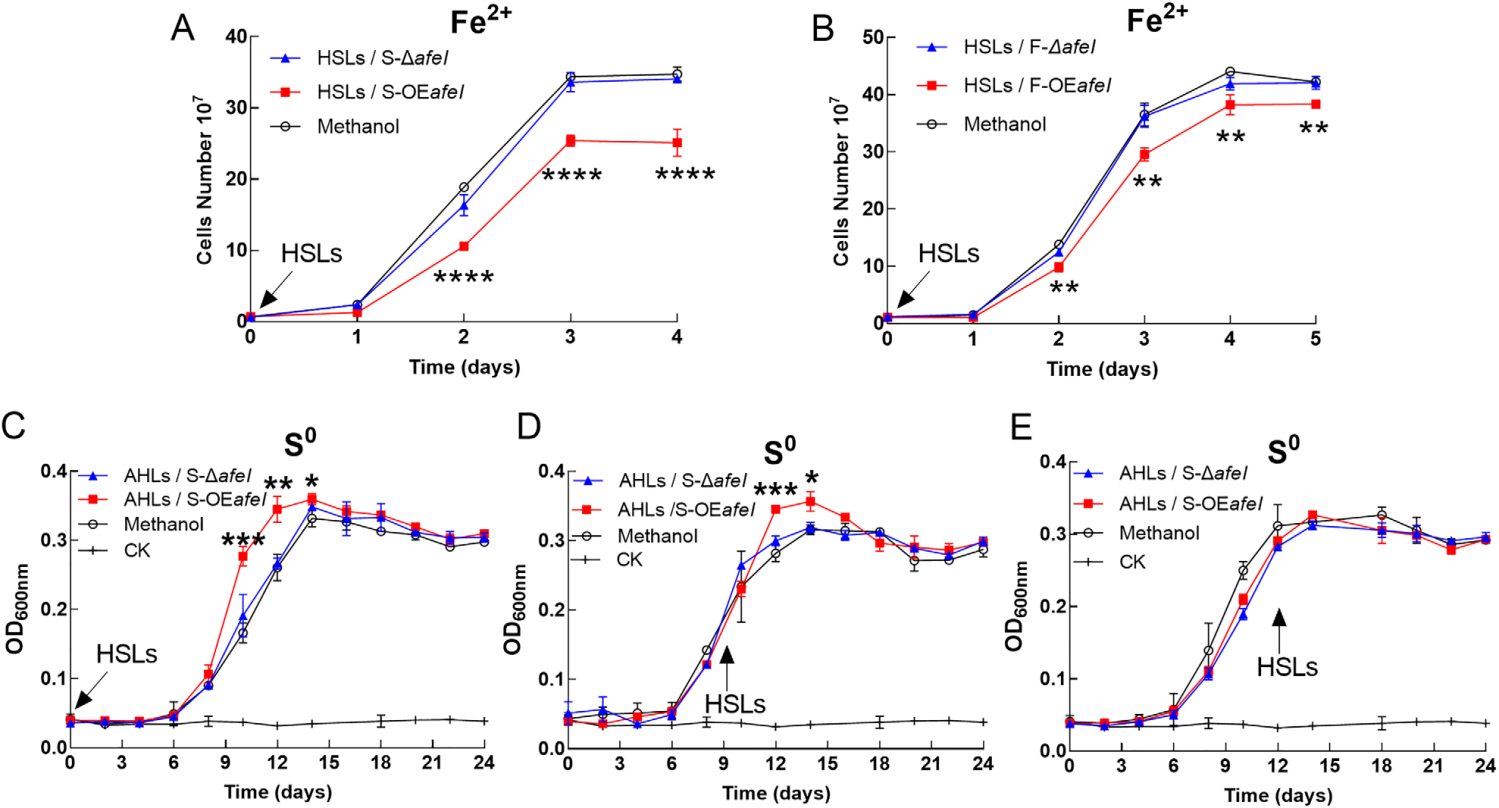
Growth of the *A*. *ferrooxidans* wild‐type strain supplemented with the extracted acyl‐HSLs. A, B. The addition of acyl‐HSLs into *A*. *ferrooxidans* cultures grown in Fe^2+^‐enriched media at the beginning of cultivation; D–F, addition of acyl‐HSLs into *A*. *ferrooxidans* cultures grown in S^0^‐enriched media at different cultivation stages. HSLs/S‐OE*afeI* and HSLs/S‐Δ*afeI* indicate acyl‐HSLs extracted from culture broths of the *afeI* overexpression and deletion strains respectively, of *A*. *ferrooxidans* grown in S^0^‐enriched media; HSLs/F‐OE*afeI* and HSLs/F‐Δ*afeI* indicate acyl‐HSLs extracted from culture broths of the *afeI* overexpression and deletion strains respectively, of *A*. *ferrooxidans* grown in Fe^2+^‐enriched media. CK indicates the blank control. [Color figure can be viewed at wileyonlinelibrary.com]

The significant influence of *afeI* overexpression on *A*. *ferrooxidans* growth in Fe^2+^‐ or S^0^‐enriched media implied that the acyl‐HSLs synthesized by AfeI could influence cell growth. To further validate this speculation, add‐back assays with *A*. *ferrooxidans* WT strain were carried out using acyl‐HSLs extracted from the culture broth of the *afeI* overexpression or deletion strains. When Fe^2+^ was used as the sole energy substrate, the addition of acyl‐HSLs extracted from S^0^‐ or Fe^2+^‐enriched *afeI* overexpression strain cultures suppressed cell growth and final bacterial population (Fig. 5A and B).

Cell growth was enhanced in *A*. *ferrooxidans* cultivated in S^0^‐enriched media upon the exogenous addition of acyl‐HSLs from S^0^‐enriched *afeI* overexpression strain cultures at the initial or mid‐growth phase (Fig. 5C and D). However, no growth advantage occurred when the acyl‐HSLs were added in the stationary growth phase (Fig. 5E). No apparent change in cell growth was observed upon the addition of extracts from Δ*afeI* cultures (Fig. 5).

Thus, the regulatory effects of the extracted acyl‐HSLs were consistent with the effects obtained by overexpressing *afeI*, indicating the influence of the AfeI‐synthesized acyl‐HSLs on cell growth of *A*. *ferrooxidans* and showing the different growth effects depending on the different energy substrates.

### Identification of AfeI‐synthesizing acyl‐HSLs in different energy substrates

To avoid interference from another acyl‐HSLs synthetase (Act) in *A*. *ferrooxidans* (Rivas *et al*., 2007), the *afeI*‐expression‐only strain Δ*act*(*afeI*) and the control strain Δ*afeI&act*(215) were constructed to identify the AfeI‐synthetized acyl‐HSLs by LC‐MS‐MS (Table 1 and Fig. S1). In S^0^‐enriched media, five types of acyl‐HSLs were verified, with [MS+H] ^+^ values of 284.22, 312.25, 272.18, 300.21 and 328.24, which were similar to the [MS+H] ^+^ values of C_12_, C_14_, 3‐OH‐C_10_, 3‐OH‐C_12_ and 3‐OH‐C_14_ respectively. In addition, these five acyl‐HSLs exhibited the characteristic protonation of homoserine lactone (*m/z* 102.05) (Morin *et al*., 2003), and the MS_2_ spectra of these five acyl‐HSLs were the same as those of the standard compounds. Because 3‐OH‐C_16_ is not commercially available, it was identified using a previously reported method (Morin *et al*., 2003; Farah *et al*., 2005). The [M+H]^+^ value of 356.2801 observed in the extracts was almost identical to the theoretical [M+H]^+^ number of 3‐OH‐C_16_ (356.2790; Fig. S1F and I). And the LC‐MS‐MS experiments detected a value of *m*/*z* 102.0541 in the MS_2_ results (356.2801), which is characteristic of acyl‐HSLs protonation. Thus, we concluded that *afeI* can synthesize six types of acyl‐HSLs (C_12_, C_14_, 3‐OH‐C_10_, 3‐OH‐C_12_, 3‐OH‐C_14_ and 3‐OH‐C_16_) in *A*. *ferrooxidans* cells grown in S^0^‐enriched media. Furthermore, the relative content of these six acyl‐HSLs in the extracts was detected via the peak area normalization method (Ni *et al*., 2019); the acyl‐HSL concentrations were found to occur in the following order: 3‐OH‐C_14_ > 3‐OH‐C_12_ > 3‐OH‐C_16_ > C_12_ > C_14_ > 3‐OH‐C_10_. In Fe^2+^‐enriched media, three acyl‐HSLs were identified with the following relative concentration order: 3‐OH‐C_14_ > 3‐OH‐C_12_ > 3‐OH‐C_16_. Thus, different signal molecules were synthesized by *afeI* depending on the presence of different energy substrates, among which 3‐OH‐C_14_ was the most abundant and may play important roles in growth and metabolism regulation. Moreover, the relative quantification results showed that the contents of acyl‐HSLs detected in *afeI* overexpression strain cultures were 2.97 and 2.47 times higher than those of the control strain in S^0^‐ and Fe^2+^‐enriched media respectively.

**Table 1:** Identification of the acyl‐HSLs in S^0‐^ or Fe^2+^‐containing medium by LC‐MS/MS.

Acyl‐HSLs	Chemical formula	[M+H]^+^ ion detected (*m*/*z*)	Structural formula	S^0^	Fe^2+^
3‐OH‐C_14_	C_18_H_33_O_4_N	328.2400	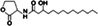	******	***
3‐OH‐C_12_	C_16_H_29_O_4_N	300.2100	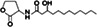	*****	**
3‐OH‐C_16_	C_20_H_37_O_4_N	356.2790	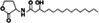	****	*
C_12_	C_16_H_29_O_3_N	284.2220	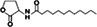	***	ND
C_14_	C_18_H_33_O_3_N	312.2530	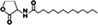	**	ND
3‐OH‐C_10_	C_14_H_25_O_4_N	272.1850	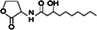	*	ND

Asterisk indicates relative concentration in the extracts; ND indicates not detected.

### Identification of functional acyl‐HSLs involved in *A*. *ferrooxidans* growth modulation

**Fig 6:**
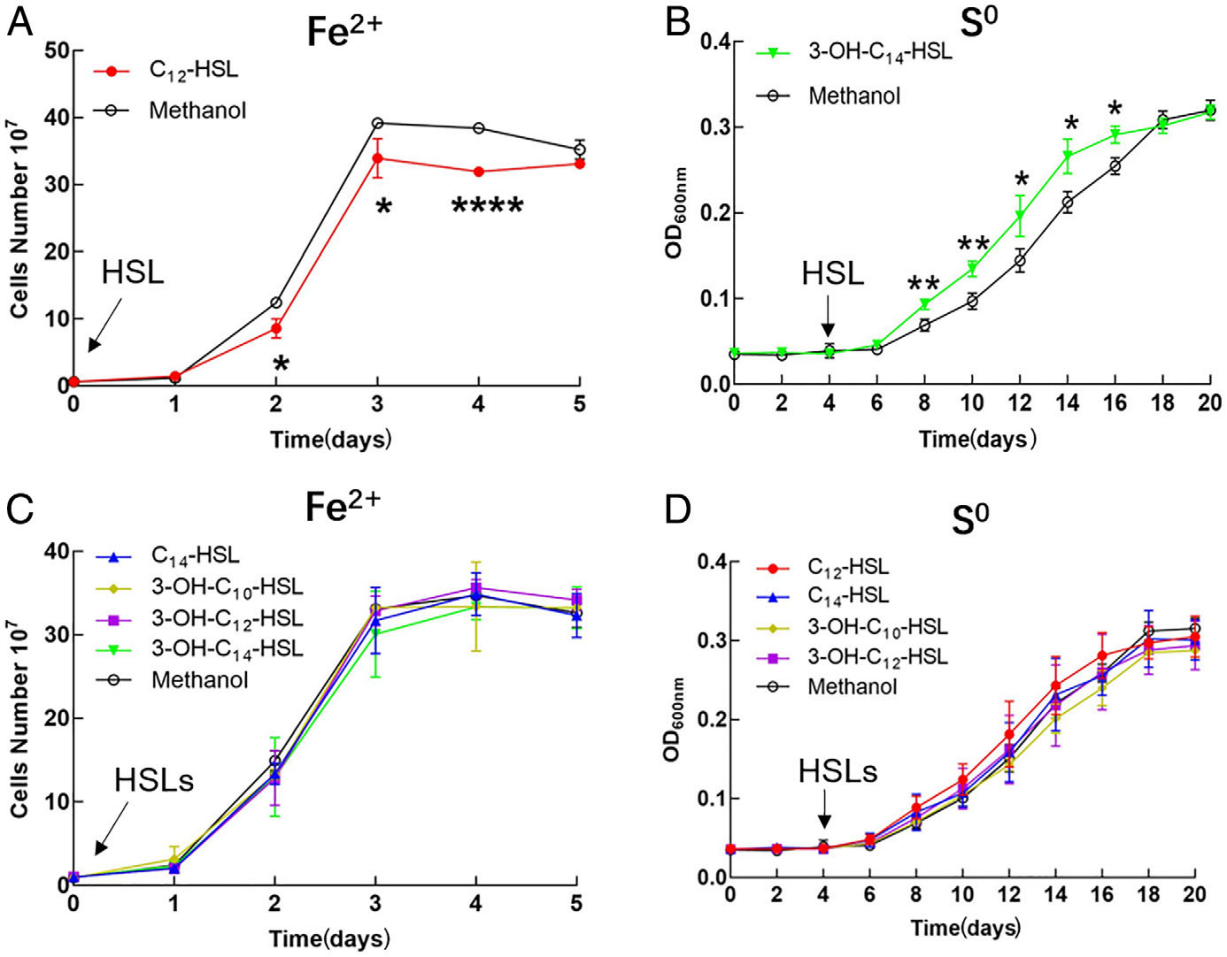
Growth curves of the *A*. *ferrooxidans* wild‐type strain with the addition of different kinds of standard acyl‐HSLs products. HSLs indicate N‐acyl homoserine lactones. [Color figure can be viewed at wileyonlinelibrary.com]

To identify the key acyl‐HSLs involved in *A*. *ferrooxidans* growth regulation, five acyl‐HSL standards, including C_12_, C_14_, 3‐OH‐C_10_, 3‐OH‐C_12_ and 3‐OH‐C_14_ were purchased to perform add‐back assays. C_12_‐HSL inhibited *A*. *ferrooxidans* growth in Fe^2+^‐enriched media (Fig. 6A), while other acyl‐HSLs had no obvious effect (Fig. 6C). Moreover, the addition of 3‐OH‐C_14_‐HSL on the 4th day stimulated cell growth at the log growth phase (Fig. 6B) and promoted EPS synthesis in *A*. *ferrooxidans* grown in S^0^‐enriched media (Fig. S2). However, adding other acyl‐HSLs to S^0^‐enriched *A*. *ferrooxidans* media did not have any statistically significant effect on cell growth (Fig. 6D). Therefore, two key signal molecules with the function of influencing *A*. *ferrooxidans* growth were discovered, including the regulation of C_12_‐HSL on *A*. *ferrooxidans* in Fe^2+^‐enriched media and the modulation of 3‐OH‐C_14_‐HSL on *A*. *ferrooxidans* in S^0^‐enriched media.

### Metabolic pathways regulated by AfeI/R in different energy substrate

The differentially expressed genes (DEGs) were detected by RNA‐seq (Table S1), and the DEGs of interest were verified by Real‐time quantitative PCR (RT‐qPCR).

**Fig 7:**
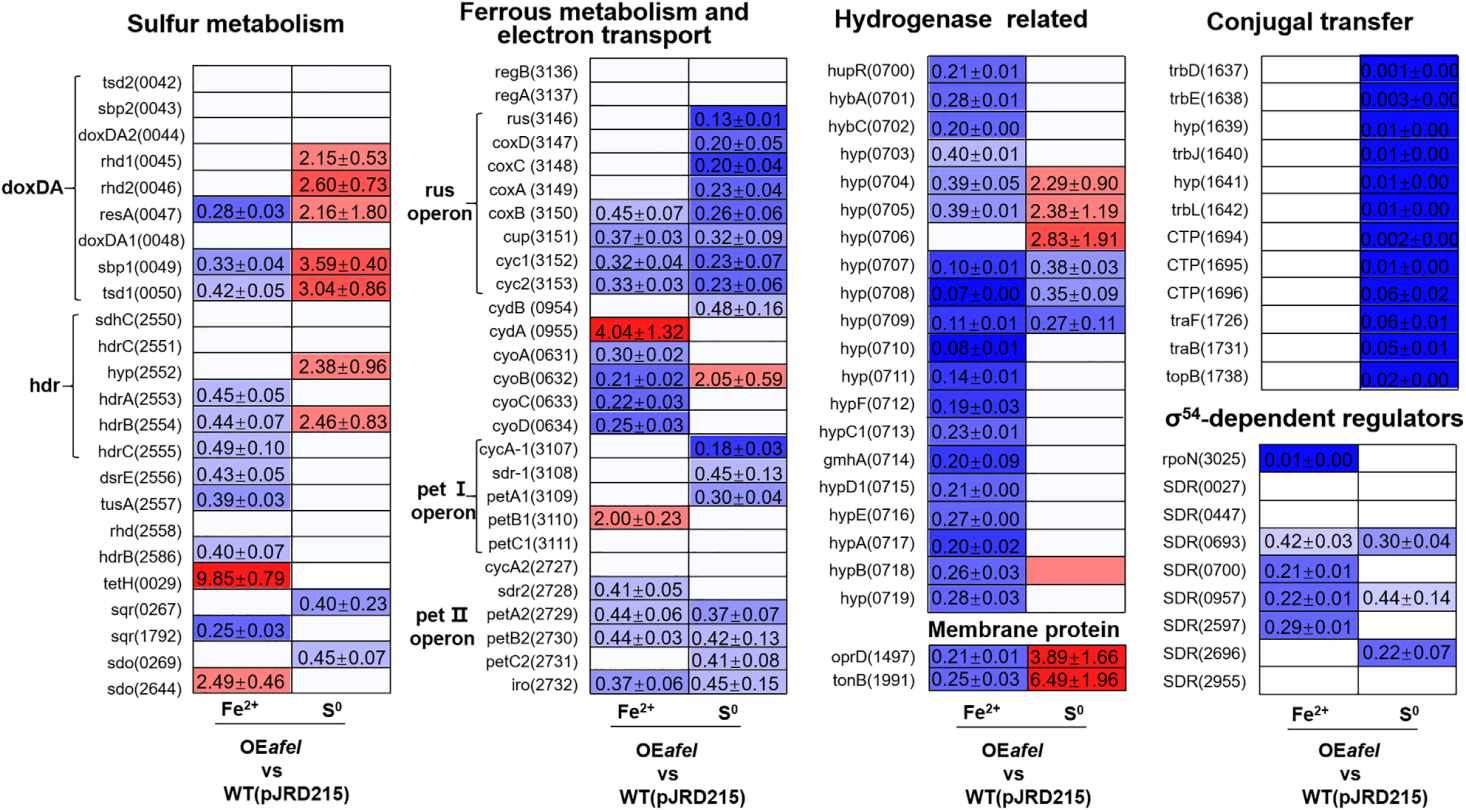
Effect of the AfeI/R QS system on the transcriptional profiles of *A*. *ferrooxidans*. This is the valid mean value of fold changes (FC) determined by RT‐qPCR. S‐OE*afeI* and Fe‐OE*afeI* represent the *afeI* overexpression in S^0^‐ and Fe^2+^‐enriched media respectively. FC ≥ 2, *P* ≤ 0.05, upregulated; FC ≤ 0.5, *P* ≤ 0.05, downregulated; 0.5 ≤ FC ≤ 2, *P* ≥ 0.05, no change (data are not shown in the figure). [Color figure can be viewed at wileyonlinelibrary.com]

Genes associated with energy metabolism were differentially expressed in the *afeI* overexpression strains in different energy substrates. In Fe^2+^‐enriched media, overexpression of *afeI* led to downregulation of genes in the *rus*, *cyo*, *pet*, *doxDA*, and *hdr* operons as well as the *dsrE*, *tusA* and *sqr* genes (AFE_1792), and upregulation of *petB1* in the *petI* operon as well as *cydA*, *tetH* and *sdo* (AFE_2644), suggesting inhibitory effects on ferrous iron‐ and sulfur‐oxidizing pathways (Fig. 7). Notably, a hydrogenase gene cluster (AFE_0700‐AFE_0719, AFE_0700 encodes a sigma54‐dependent regulator (SDR), Fig. S3) exhibited significant downregulation in this condition (Fig. 7), implying the significance of AfeI/R mediated hydrogen metabolism on the growth of *A*. *ferrooxidans* in Fe^2+^‐media. When S^0^ was used as the energy substrate, overexpression of *afeI* resulted in the obvious upregulation of genes in the *doxDA*, and *hdr* clusters, and downregulation of the *sqr* (AFE_0267) and *sdo* (AFE_0269) genes (Fig. 7). The high expression levels of the majority of sulfur‐oxidizing genes indicated the sulfur‐oxidizing system was stimulated by overexpression of *afeI* in S^0^‐culture. Simultaneously, the iron‐oxidizing system, *rus* operon and *pet* operon, was significantly inhibited in the S^0^‐cultivated *afeI* overexpression strain (Fig. 7). Therefore, AfeI/R could effectively modulate the metabolisms of sulfur, iron and hydrogen to control cell growth and population size of *A*. *ferrooxidans* in different energy‐substrates.

The transcription of eight SDRs was clearly influenced by the *afeI* (Fig. 7). Upon growth on Fe^2+^‐containing media, there were four SDR genes (AFE_0693, 0700, 0957 and 2597) as well as the sigma 54 gene (AFE_3025) downregulated in the *afeI* overexpression strain (Fig. 7). Upon growth on S^0^‐containing media, there were three SDR genes (AFE_0693, 0957 and 2696) downregulated in the *afeI* overexpression strain. Thus, the differential expression of SDR genes indicated the strong and extensive impacts of the AfeI/R QS system on the sigma54‐regulated pathways in different energy‐substrates.

Genes associated with membrane permeability were differentially expressed in the *afeI* overexpression *A*. *ferrooxidans* strains (Fig. 7). The *oprD* (AFE_1497) and *tonB* (AFE_1991) genes involved in EPS transport (Abbas *et al*., 2007; Zhang *et al*., 2018) were markedly downregulated in the *afeI* overexpression strain grown on Fe^2+^‐media, while both of these genes were upregulated in the *afeI* overexpression strain grown on S^0^‐media (Fig. 7). Conjugal transfer related genes were markedly downregulated in the *afeI* overexpression strain grown on S^0^‐media, while both of these genes were no changed in the *afeI* overexpression strain grown on Fe^2+^‐media (Fig. 7)

The transcription levels caused by *afeI* overexpression were different under different energy substrates. This indicated that the versatile regulation of AfeI/R QS system in *A*. *ferrooxidans* was dependent on energy substrates.

## Discussion

In this study, we revealed that AfeI/R‐like QS system could not only function in the S^0^‐cultivating process through the use of key acyl‐HSLs but also play an important regulatory role in bacterial ferrous iron oxidation, cell growth and quorum size in Fe^2+^‐enriched media. Sulfur and ferrous iron are the two crucial energy substrates for acidophiles, which could affect population development and community formation in the natural habitats of AMD sites and terrestrial hot springs and in the industrial bioleaching processes (Rawlings, 2002; Rohwerder *et al*., 2003). The prevalence of AfeI/R system in the sulfur‐ and ferrous iron‐oxidizing species of *Acidithiobacillus* (Fig. 1), together with the inhibiting effect caused by the overexpression of *afeI* or the addition of exogenous acyl‐HSLs (Figs 3A, C, 5A, B and 6A), implied that the AfeI/R system in *Acidithiobacillus* have evolved the regulatory capacity on bacterial ferrous metabolism. To our knowledge, this is the first report that QS system is involved in the regulation of bacterial ferrous metabolism, cell growth and population density in Fe^2+^ cultivation. Moreover, our results manifested that the key acyl‐HSL‐inducible EPS synthesis could influence the sulfur oxidation and cell growth of *A*. *ferrooxidans* in the S^0^‐enriched media (Figs 3E, G and 4B, D, Fig. S2).

**Fig 8:**
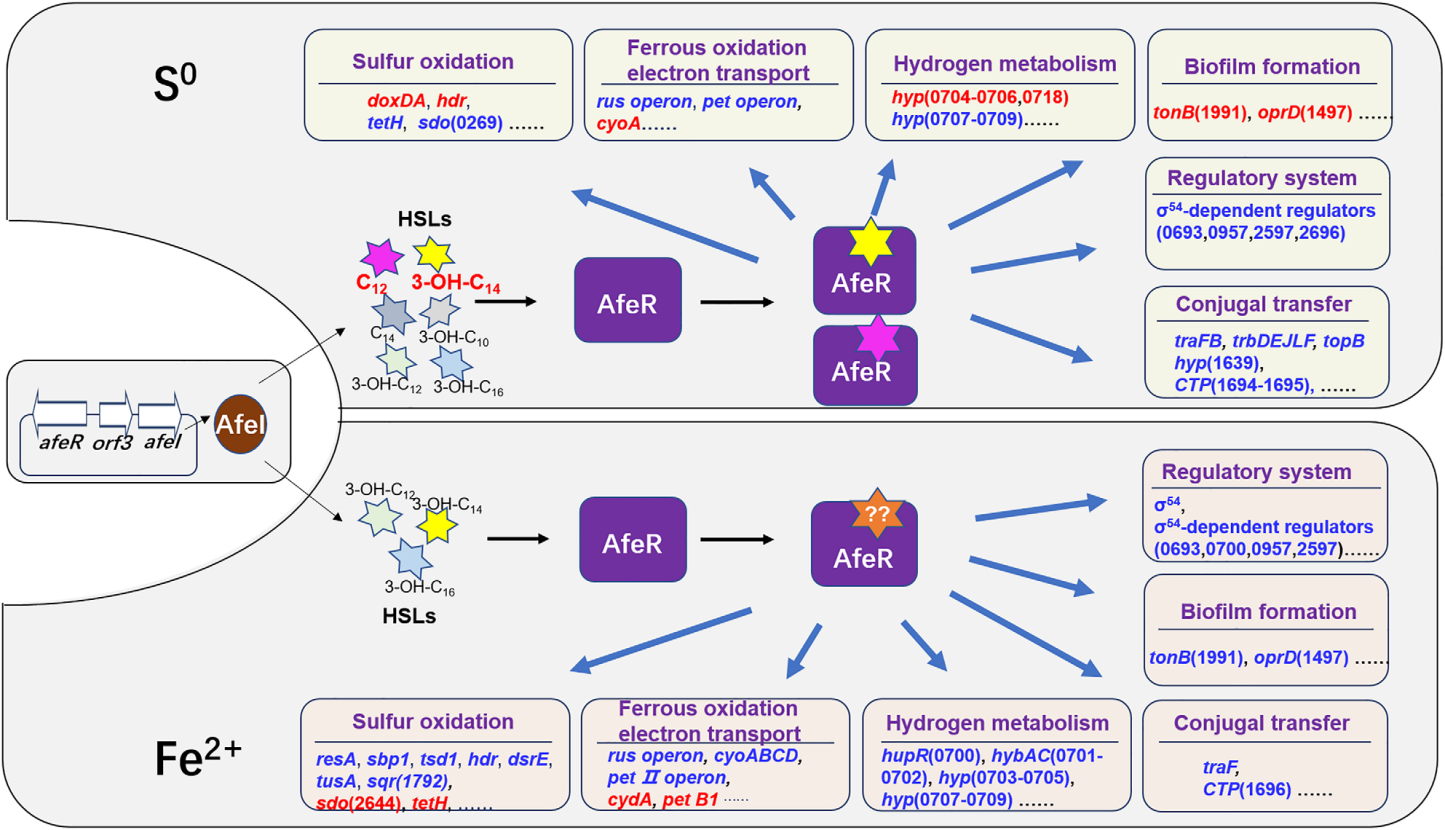
Model of the substrate‐dependent AfeI/R‐regulated network in *A*. *ferrooxidans*. CTP indicates conjugal transfer protein; hyp indicates hypothetical protein. [Color figure can be viewed at wileyonlinelibrary.com]

An AfeI/R‐mediated energy‐substrate‐dependent regulation model in *A*. *ferrooxidans* was proposed on the basis of the extensive influences of energy substrates on the synthesis of acyl‐HSLs, the regulatory effects of signal molecules and the AfeI/R‐regulated pathways/systems (Fig. 8). According to the energy substrates, AfeI could synthesize different kinds of acyl‐HSLs, and some of the signal molecules could function as the ‘stimulator’ or ‘inhibitor’ with the prerequisite of specific energy substrate to regulate the expression of genes involved in metabolic pathways and regulatory systems. Thus, the AfeI/R‐mediated versatile regulation could offer varied strategies for *A*. *ferrooxidans* to modulate its genes expression and phenotypes in sulfur‐ and ferrous iron‐contained extremely acidic environments.

The regulatory effects of AfeI/R on sulfur metabolism and cell growth of *A*. *ferrooxidans* in S^0^‐enriched media could be attributed to its regulation of EPS synthesis. EPS can enhance the adhesion of cells to solid energy substrates, and provide an active reaction space between the cells and the surface of the substrates (Gehrke *et al*., 1998; Harneit *et al*., 2006). Overexpression of *afeI* stimulated the EPS synthesis (Fig. 4B), which in turn enhanced the attachment and bioerosion of cells on elemental sulfur (Fig. 4D). This process could accelerate the activation and oxidation of extracellular elemental sulfur (Gehrke *et al*., 1998; Harneit *et al*., 2006), resulting in the increase of sulfur‐oxidizing capacity (upregulation of sulfur‐oxidizing genes in Fig. 7). Thus, we confirmed that the AfeI/R‐mediated regulation on EPS synthesis could influence the sulfur metabolism and cell growth of *A*. *ferrooxidans* in S^0^‐enriched media. The add‐back assays suggested that 3‐OH‐C_14_‐HSL could function as a ‘stimulator’ to regulate EPS synthesis and cell growth of *A*. *ferrooxidans* in S^0^‐enriched media (Fig. 6B and Fig. S2). Therefore, the 3‐OH‐C_14_‐HSL‐inducible EPS synthesis could be the regulatory strategy of AfeI/R for *A*. *ferrooxidans* to modulate its sulfur metabolism and cell growth in S^0^‐enriched media. The increase in the levels of signal molecules upon overexpression of *afeI* or addition of exogenous acyl‐HSLs could promote sulfur metabolism and cell growth in favorable growth environments (lag and log phases) but did not change the final population density in the S^0^‐enriched media (Figs 3E, G, 5C–E and 6B). Thus, the role of AfeI/R could be defined as an ‘accelerator’ for the development of the population, but not a ‘quorum maker’ for *A*. *ferrooxidans* in S^0^‐enriched media.

The strong inhibitory effect of either overexpression of *afeI* or addition of acyl‐HSLs on the ferrous iron oxidation, cell growth and quorum size of *A*. *ferrooxidans* in Fe^2+^‐enriched media (Figs 3A, C, 5A, B and 6A) suggested that AfeI/R could be considered as an efficient ‘inhibitor’ for *A*. *ferrooxidans* cultivated in Fe^2+^‐enriched media. Neither overexpression nor deletion of *afeI* was observed to have an effect on EPS synthesis for cells grown in Fe^2+^‐enriched media (Fig. 4A). These results provide two evidence: first, AfeI/R could not regulate the synthesis of EPS in the Fe^2+^‐enriched media; second, the QS‐mediated regulation was not caused by EPS in this condition. The obvious downregulation of genes encoding electron transporter and respiratory chain (*rus*, *pet* and *cyo* operon) may be the reason for the decrease of ferrous oxidation capacity and cell density of the *afeI* overexpression stain in Fe^2+^‐enriched media (Figs 3A, C and 7). The striking downregulation of a hydrogenase gene cluster in *afeI* overexpression strain provides another clue for understanding the AfeI/R‐mediated regulation in Fe^2+^‐media (Fig. 7 and Fig. S3). The HupR‐containing hydrogenase genes cluster was suggested to catalyze the conversion of dihydrogen to protons and electrons in *A*. *ferrooxidans* (Schröder *et al*., 2007; Kalms *et al*., 2018). The significant downregulation of the hydrogenase cluster probably reduced the generation of intracellular protons, which likely altered the pH homeostasis that is important for ATP biosynthesis (Lubitz *et al*., 2014; Hansen and Perner, 2016; Kalms *et al*., 2018). Thus, AfeI/R may participate in the regulation of hydrogen metabolism, which could be another reason for the decrease of cell growth and population density of the *afeI* overexpression stain in Fe^2+^‐enriched media.

The energy substrates could influence both the types of acyl‐HSLs produced by AfeI and the regulatory function of these acyl‐HSLs. Due to the presence of other potential acyl‐HSL‐synthase genes (*act*) in *A*. *ferrooxidans*, the specific acyl‐HSLs produced by AfeI remained unclear. Therefore, we constructed the *afeI*‐expression‐only strain Δ*act*(*afeI*) and the *afeI* and *act* double knockout strain Δ*afeI&act*(215) to determine which acyl‐HSLs were generated by AfeI. Our results demonstrated that acyl‐HSLs with C‐3 hydrogen and hydroxyl substituents were synthesized by AfeI in S^0^‐enriched media, whereas only 3‐hydroxyl‐HSLs were found in the Fe^2+^‐enriched media (Table 1). Interestingly, previously reported acyl‐HSLs in *A*. *ferrooxidans* (i.e., 3‐oxo‐HSLs and 3‐hydroxy‐C_8_‐HSL) were not detected in this study (Farah *et al*., 2005). Synthesis of acyl‐HSLs requires S‐adenosylmethionine substrates and an acylated acyl carrier protein (acyl‐ACP) from the fatty acid synthesis pathway, and growth conditions could influence the availability of acyl‐ACP substrates (Parsek and Greenberg, 2000; Teplitski *et al*., 2003). Thus, the differences between the acyl‐HSLs observed herein and in previous reports could be due to the influence of other potential QS systems (Act), as well as differences in cultivation methods and environments. Abundant 3‐OH‐C_14_‐HSL was detected in both Fe^2+^‐ and S^0^‐enriched media (Table 1), but this compound was functional only in the S^0^‐enriched media (Fig. 6B and C). Although C_12_‐HSL showed an inhibitory effect on the growth of *A*. *ferrooxidans* in Fe^2+^‐enriched media (Fig. 6A), it was detected only in the S^0^‐media but not in Fe^2+^‐media (Table 1). Due to the lack of a 3‐OH‐C_16_ standard, an add‐back assay for this acyl‐HSL was not performed in this study. The role of 3‐OH‐C_16_‐HSL and other unidentified acyl‐HSLs produced by AfeI in the regulation of *A*. *ferrooxidans* in Fe^2+^‐enriched media remains an open question for future studies. These results indicated that different signal molecules were used by AfeI/R to modulate specific regulation pathways in different substrates. Therefore, the substrate‐dependent synthesis and regulation of the acyl‐HSLs is a key characteristic of the AfeI/R QS system, which probably allow *A*. *ferrooxidans* to effectively cope with the different energy substrates in the growth environments.

The significant changes in the transcriptomes of *afeI* knockout and overexpression strains both in S^0^‐ and Fe^2+^‐enriched media (Fig. 7 and Table S1) suggested that AfeI/R is an important means for *A*. *ferrooxidans* regulating its genes transcription in different energy substrates. In the QS‐mediated gene regulation system, the receptor reacts to a signal molecule and then binds to the *lux‐box* sequence to control genes transcription (An *et al*., 2006; Schaefer *et al*., 2008). The *lux‐box* sequences in *A*. *ferrooxidans* were predicted via the bioinformatic approach (Banderas and Guiliani, 2013), and the *lux‐box* region upstream of the *afeI* gene was determined via gel mobility shift assays (Mamani *et al*., 2016). Based on these results, DEGs containing *lux‐box* sequences were found in this study, including sulfur metabolism gene (AFE_0269), *pet* operon (AFE_3107‐3111), sigma‐54‐dependent transcriptional regulator gene (AFE_0693 and 0957) and conjugal transfer gene (AFE_1694). These results implied the direct regulation of AfeR on these genes, highlighting the control of the AfeI/R QS system on these pathways in *A*. *ferrooxidans*.

The AfeI/R‐mediated substrate‐dependent versatile regulation could be a noteworthy characteristic of QS regulation in these chemoautotrophic sulfur‐ and ferrous iron‐oxidizing bacteria, differentiating to other reported LuxI/R‐like QS regulation in bacteria. AfeR, for its important roles in discriminating different acyl‐HSLs and modulating genes transcription, maybe a key factor for the formation of the AfeI/R‐mediated versatile regulation in different energy‐substrates. The homology model suggested that AfeR has a receptor domain that binds to signal molecules and a regulatory domain that interacts with DNA to regulate gene transcription (Zhang *et al.,* 2002; Soulère *et al*., 2008). The receptor domain of the LuxR family has evolved differently to suit its hosts (Bottomley *et al*., 2007). Alignment of AfeR and LuxR family protein sequences revealed that the acyl‐HSLs receptor domain has highly conserved key amino acid residues (Tyr58, Trp63, Asp75, Trp90, Ala105 and Gly113), as well as some amino acid residues that are less conservative than other strains (amino acids colored in cyan in Fig. S4). Therefore, the receptor domain of the AfeR may evolve some unique features, which leads to the different recognition ability of AfeR on acyl‐HSLs in different energy substrates. In addition, it was reported that the conformations of an acyl‐HSL are diverse, such as the linear and curved‐shape alkyl chain (Soulère *et al*., 2008). Acyl‐HSLs with different conformations showed different affinities to the AfeR receptor (Soulère *et al*., 2008). Besides, it has been reported that the pattern of acyl‐HSLs produced by a single strain depends largely on the media (Teplitski *et al*., 2003). Therefore, it is speculated that different energy substrates may affect the conformations of the acyl‐HSLs, which in turn affects the binding of the acyl‐HSLs to the AfeR receptor, and ultimately leads to the differences in signal recognition and gene regulation of AfeI/R system in different energy substrates. Therefore, the structural differences between AfeR and other LuxR family proteins as well as the change of the conformations of signal molecules in different substrates could contribute to the formation of AfeI/R‐mediated substrate‐dependent versatile regulation in the sulfur‐ and ferrous iron‐oxidizing bacteria.

In summary, AfeI/R have evolved distinct regulatory strategies specific to the energy substrates, and the AfeI/R‐mediated substrate‐dependent regulation could be an important mechanism employed by these sulfur‐ and ferrous iron‐oxidizing bacteria to maintain the balance between their energy metabolisms and population development in the sulfur‐ and ferrous iron‐containing extremely acidic environments. This study would be a basis for further studies on the ecological functions of AfeI/R‐like QS systems in the natural habitats and provide new insights in the synthetic biological research of the chemoautotrophic bacteria.

## Experimental procedures

### Bacteria and growth conditions

The bacteria and plasmids used in this study are listed in Table 2. *Escherichia coli* was cultivated at 37 °C in LB media (Sambrook *et al*., 1982). The *A*. *ferrooxidans* ATCC 23270 strain was grown in 9K inorganic salt media with Fe^2+^ (10 g/L) or S^0^ (0.8% w/v) as energy sources, and the pH was adjusted to 2.0 using H_2_SO_4_. Starkey‐Na_2_S_2_O_3_ agar media was used for *A*. *ferrooxidans* plate cultures (Wang *et al*., 2012). Cell growth in the 9K‐S^0^ and 9K‐Fe^2+^ media was monitored via OD_600 nm_ measurements and the microscopic counting method respectively.

**Table 2:** Bacteria and plasmids used in this study.

Strain and plasmids	Description	Source
**Strain**		
*Acidithiobacillus ferrooxidans* ATCC 23270	Type strain	ATCC
WT	Wild type	ATCC
WT (pJRD215)	Wild type including the plasmid of pJRD215	This study
Δ*afeI*	*afeI* gene deletion	This study
OE*afeI*	overexpress *afeI* gene, including the plasmid of pJRD215‐P_*tac*_‐*afeI*	This study
Δ*act* (*afeI*)	*act* gene deletion and *afeI* gene overexpress	This study
Δ*afeI*&*act*	Both *afeI* and *act* genes deletion	This study
*Escherichia coli*		
DH5α	F^‐^φ80d*lac*ZΔM15Δ*(lacZYA‐argF)*U169 *end A1 recA1 hsdR17(rk* ^*‐*^,*mk* ^*+*^ *) supE44λ‐thi‐1 gyr96 rela1 phoA*	TransGen Biotech
S17‐1*λpir*	Tp^r^ Sm^r^ *recAthi pro r*_*k*_^*‐*^ *m*_*k*_^*‐*^RP4:2‐Tc:MuKmTn7λpir	Bilecen and Yildiz (2009)
SM10	Km^r^ *thi‐1 thr leu tonA lacY supE recA*RP4‐2‐Tc::Mu	Simon *et al*. (1983)
**Plasmids**		
pSDUDI	Suicide plasmid; Ap^r^ Km^r^ oiTRP4 multi‐cloning sites	Wang *et al*. (2016)
pSDUDI::afeI(UHA + DHA)	Suicide plasmid for Δ*afeI* construction	This study
pSDUDI::act(UHA + DHA)	Suicide plasmid for Δ*act* construction	This study
pMSD1‐I‐SecI	pMSD containing the *I‐SecI* gene	Wang *et al*. (2012)
pJRD215	Sm^r^ Km^r^ IncQ Mob^+^	Davison *et al*. (1987)
pJRD215‐P_*tac*_‐*afeI*	Sm^r^ Km^r^ IncQ Mob^+^ P_*tac*_ *afeI* gene	This study

### Mutant strain construction

The sequences for all primers used in this section are listed in Table S2. The markerless deletion of the *afeI* gene (AFE_1999) in the *A*. *ferrooxidans* ATCC 23270 was performed as described previously (Wang *et al*., 2012). Two homologous arms were first amplified using the IUPF/R and IDWF/R primer pairs and ligated to the pSDUDI plasmid. The generated suicide pSDUDI‐HomafeI plasmid was then transferred into *A*. *ferrooxidans* via conjugation (Peng *et al*., 1994). Single‐crossover recombinant strains were then selected. Then, the pMSD1‐I‐SecI plasmid was conjugated into the single‐crossover recombinants, resulting in a second homologous recombination to generate the gene knockout and wild‐type (WT) reversion. The Δ*afeI* was then identified via PCR using three primer pairs: P1F/P1R, P2F/P2R and P3F/P3R; the purified P1F/P1R‐amplified PCR fragments were sequenced to confirm the mutation.

The *afeI* gene and the *tac* promoter were amplified via PCR using the PtacF/PtacR and PIF/PIR primer pairs respectively. The two fragments were digested and ligated into the pJRD215 plasmid. The pJRD215‐P_*tac*_‐*afeI* plasmid was conjugated into the *A*. *ferrooxidans* ATCC 23270 strain to construct the *afeI* overexpression strain. PCR amplification using plasmid‐specific P4F/R primers was performed to confirm the overexpression strain.

An *act* gene‐specific suicide plasmid was produced with the ACTUPF/R, ACTDWF/R primer pairs. The generated plasmid was then conjugated into WT and Δ*afeI*. Δ*act* was identified using primers PA1F/R, PA2F/R and PA3F/R. Δ*afeI*&*act* was identified using primers P1F/R, P2F/R, P3F/R, PA1F/R, PA2F/R and PA3F/R. Then, the pJRD215‐P_*tac*_‐*afeI* and pJRD215 plasmids were conjugatively transferred into Δ*act* and Δ*afeI*&*act* generating strains Δ*act*(*afeI*) and Δ*afeI*&*act* (pJRD215) respectively.

### Determination of Fe^2+^ and SO_4_^2^ ^‐^ concentrations in culture media

The concentration of Fe^2+^ in the liquid media was determined via the o‐phenanthroline method as described previously (Herrera *et al*., 1989), whereas the concentration of SO_4_^2‐^ was measured via ion chromatography (ICS‐1100AR, DIONEX, USA) (Miura and Kawaoi, 2000).

### EPS extraction and analysis

EPS extraction was performed as described previously (More *et al*., 2014; Xiao *et al*., 2017). Cells were collected by centrifugation and adjusted to their final concentration (OD_600 nm_ = 1). Then, 1 ml of these cell suspensions were centrifuged at 12 000*g* for 1 min at 4 °C. The cells were then resuspended in 4 ml of TNE buffer (10 mM Tris, 100 mM NaCl, 5 mM EDTA, pH = 7.5) and centrifuged at 12 000*g* for 10 min. The pellet was then resuspended in 4 ml TNE + SDS (0.1%). After a 5‐min reaction period at room temperature, the samples were centrifuged at 12 000*g* for 10 min to obtain EPS extracts. Afterward, the extracts were washed three times with TNE buffer and eluted in 50 mM Tris (pH 7.5). The total carbohydrate content in the EPS extracts was determined using the anthrone‐sulfuric acid method (Ding *et al*., 2019). The protein concentration in the EPS extracts was measured using the Modified Bradford Protein Assay Kit (Sangon Biotech). The experiments were performed three times and each sample was set three biological replications. Statistical analysis was conducted via Student's *t*‐test using the GraphPad Prism software (version 7.0; GraphPad).

### Sulfur coupon preparation and SEM

Sulfur coupons were prepared by melting sulfur powder and then pouring the liquid sulfur on a glass coverslip to cool and solidify (Bellenberg *et al*., 2014). The cell solutions were added to the sulfur‐coupon‐contained medium, and the cell density was adjusted to OD_600_ = 0.1. After 8 days of cultivation, the sulfur coupons were taken out, fixed with 2.5% glutaraldehyde, dehydrated in a series of graded ethanol solutions and critical point‐dried. After gold sputtering, the sulfur coupons were observed by SEM (Quanta 250 FEG, FEI) (Liu *et al*., 2003).

### Crude acyl‐HSL extraction and identification

The cells were cultivated until they reached the stationary growth phase (OD_600_ = 0.30–0.32), after which 5 L of culture was collected and centrifuged at 12 000*g* for 5 min. The supernatant from the culture broth was extracted twice using an equal volume of HPLC‐grade dichloromethane (Rivas *et al*., 2005; Ruiz *et al*., 2008). The residues were dissolved in 1 mL of HPLC‐grade methanol and stored at −20 °C. Acyl‐HSLs extraction and identification were performed three times. The acyl‐HSL extracts were identified via liquid chromatography with tandem mass spectrometry (LC‐MS‐MS; Altimate3000 (LC) Thermo Fisher, USA; ImpactHD (MS), Bruker, Germany) as described previously (Morin *et al*., 2003). C_12_, C_14_, 3‐OH‐C_10_, 3‐OH‐C_12_ and 3‐OH‐C_14_ standards were purchased from Sigma (USA). Relative acyl‐HSL quantification in the extracts was achieved by calculating the peak area from the LC‐MS‐MS results (Ni *et al*., 2019).

### Add‐back experiments

300 μl and 600 μl of the acyl‐HSLs extracts from the S^0^‐ and Fe^2+^‐enriched media were respectively added to 150 ml of media. The extract add‐back assays in S^0^‐enriched media were performed at 4th, 9th and 12th day. When Fe^2+^ was used as an energy substrate, the extracts were only added at the beginning of cultivation. The concentration used for each acyl‐HSL standard was 10 μM. The acyl‐HSLs were added on the 4th day in S^0^‐enriched media and on the 0th day in Fe^2+^‐enriched media. The experiments were performed three times with three biological replications.

### RNA extraction, real‐time quantitative PCR and RNA sequencing

RNA was extracted from cell samples in the mid‐log growth phase. The RNAprep Pure Cell/Bacteria Kit (Tiangen, China) was used for all RNA extractions according to the manufacturer's instructions. The extracted RNA was then visualized via formaldehyde degeneration electrophoresis (Rivas *et al*., 2005). The A260 value and A260/A280 ratio were measured to determine RNA concentration and purity respectively. Reverse transcription was performed using the PrimeScript™ RT Reagent Kit (TaKaRa, China). RT‐qPCR reactions were performed in a Roche LightCycler480 (Roche, USA) using the SYBR®Premix Ex Taq™ (TaKaRa) enzyme; *alaS* was used as a reference gene (Nieto *et al*., 2009). The 2^‐ΔΔCt^ method was used to analyse relative changes in gene expression (Livak and Schmittgen, 2001). RT‐qPCR primers are listed in Table S2.

RNA sequences were supplied by Novogene, China. The raw data of RNA‐seq were deposited in NCBI with accession numbers SRR9208397, SRR9208398, SRR9208395, SRR9208396, SRR9208401, SRR9208402, SRR9208399 and SRR9208400. The 179 genes were randomly selected to verify the DEGs obtained by RNA‐seq. The original data of DEGs and verification results of RT‐qPCR were listed in supplementary Table S1. The primers used in the RT‐qPCR were listed in Table S2.

### Statistical analysis

All experiments were performed three times with three biological replications. One‐way analysis of variance coupled with Bonferroni's multiple comparison test was used to compare. Statistical analysis was conducted via the Student's *t*‐test. All statistical analyses were performed using the GraphPad Prism software (version 7.0). Statistical significance is indicated with asterisks (**** indicates *P* < 0.0001, *** indicates *P* < 0.001, ** indicates *P* < 0.01 and * indicates *P* < 0.05) in the results section.

## Conflict of Interest

The authors declare that the research was conducted in the absence of any commercial or financial relationships that could be construed as a potential conflict of interest.

## Supporting information


**Appendix S1**: Supporting Information
