# Supplementary material for: The substrate‐dependent regulatory effects of the AfeI/R system in Acidithiobacillus ferrooxidans reveals the novel regulation strategy of quorum sensing in acidophiles
Source: Environ Microbiol. 2020 Aug 2;23(2):757–73. doi: 10.1111/1462-2920.15163 (PMC7984328; doi:10.1111/1462-2920.15163)
Supplement: Supplementary file 1 — Appendix S1: Supporting Information [file EMI-23-757-s001.DOCX]

**Supplement materials**

**
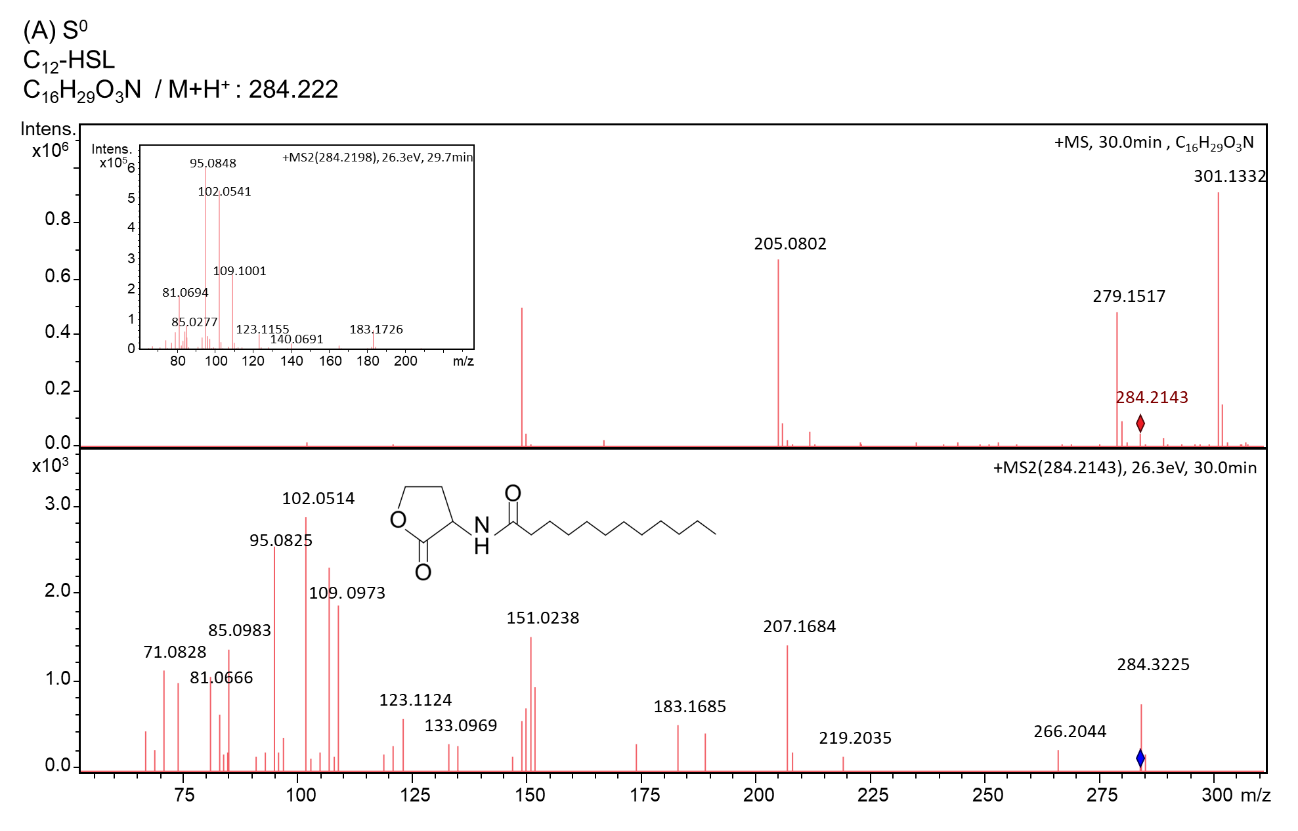
**

**
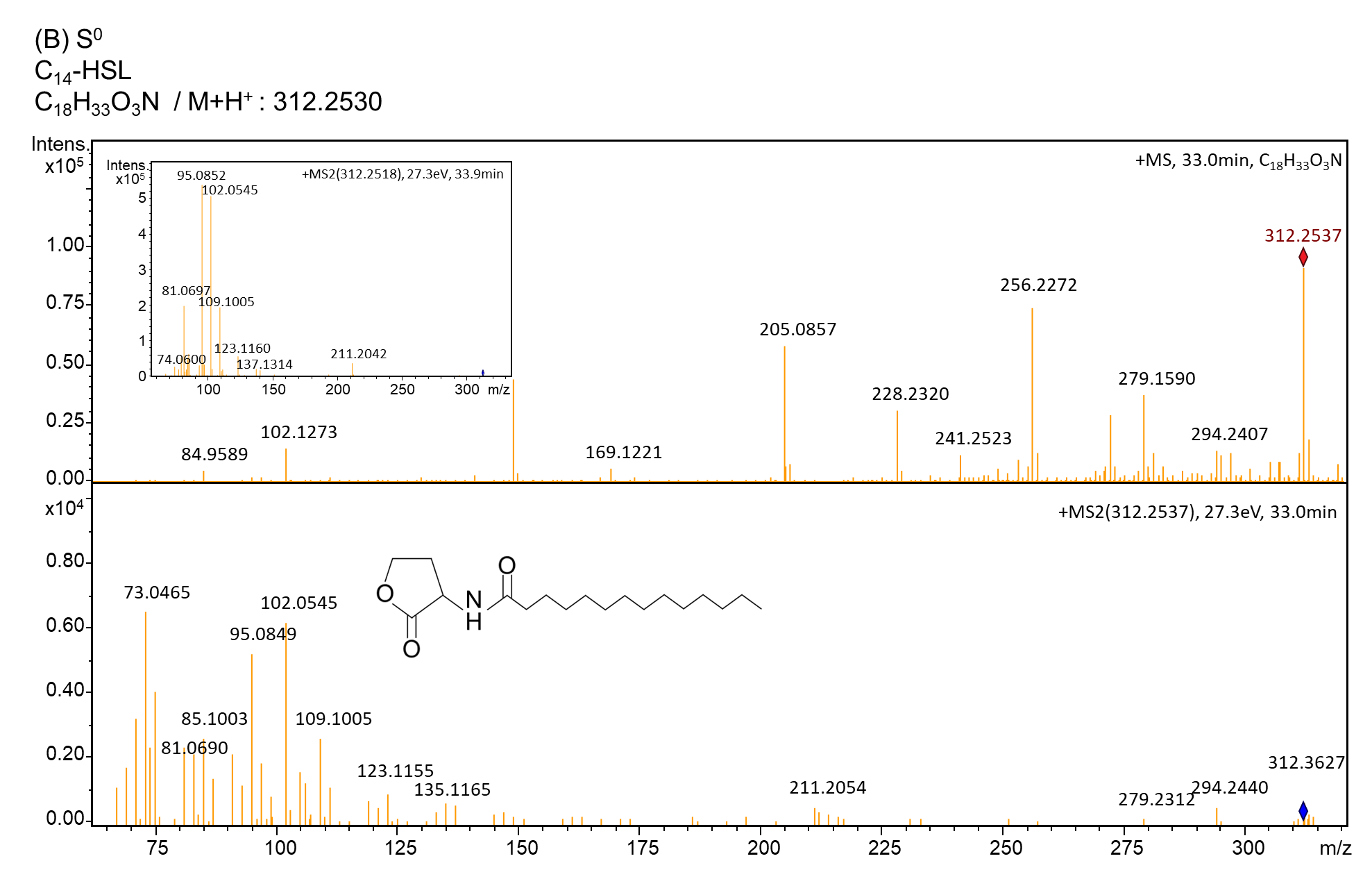
**

**
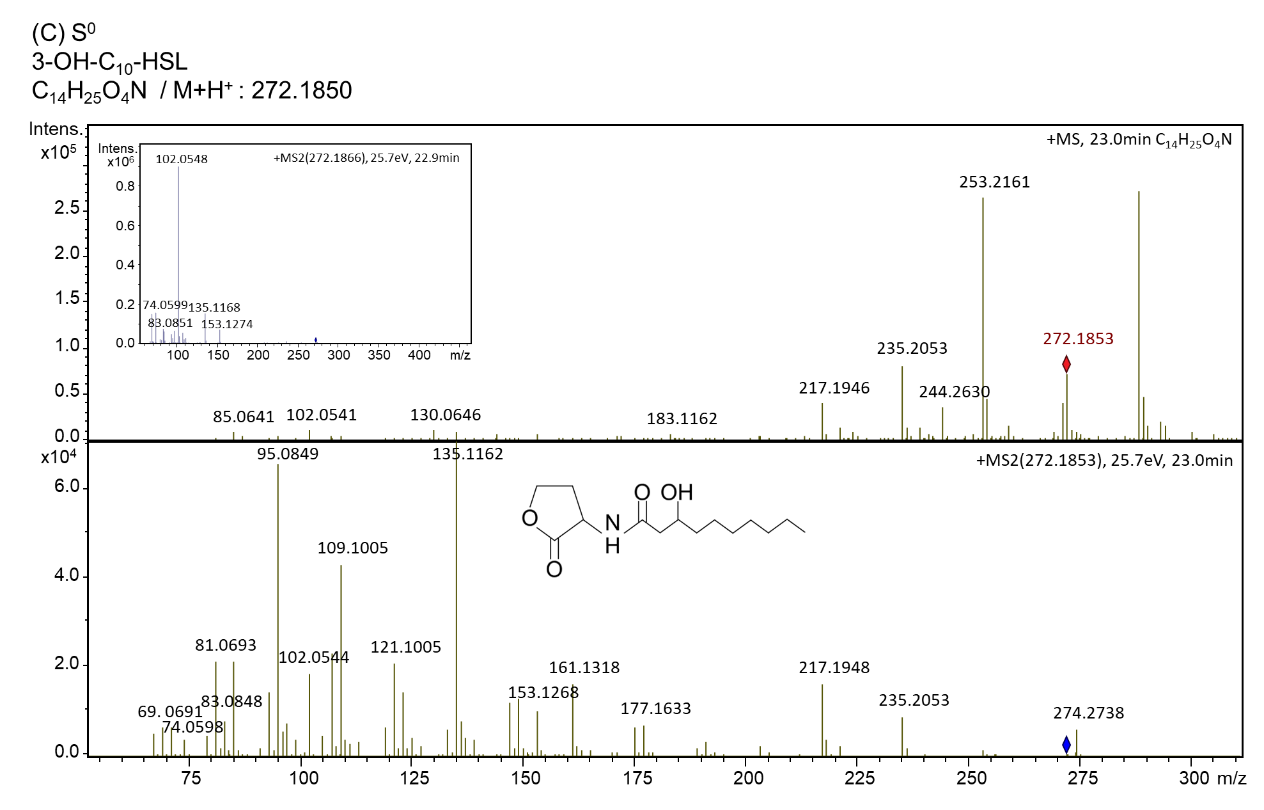
**

**
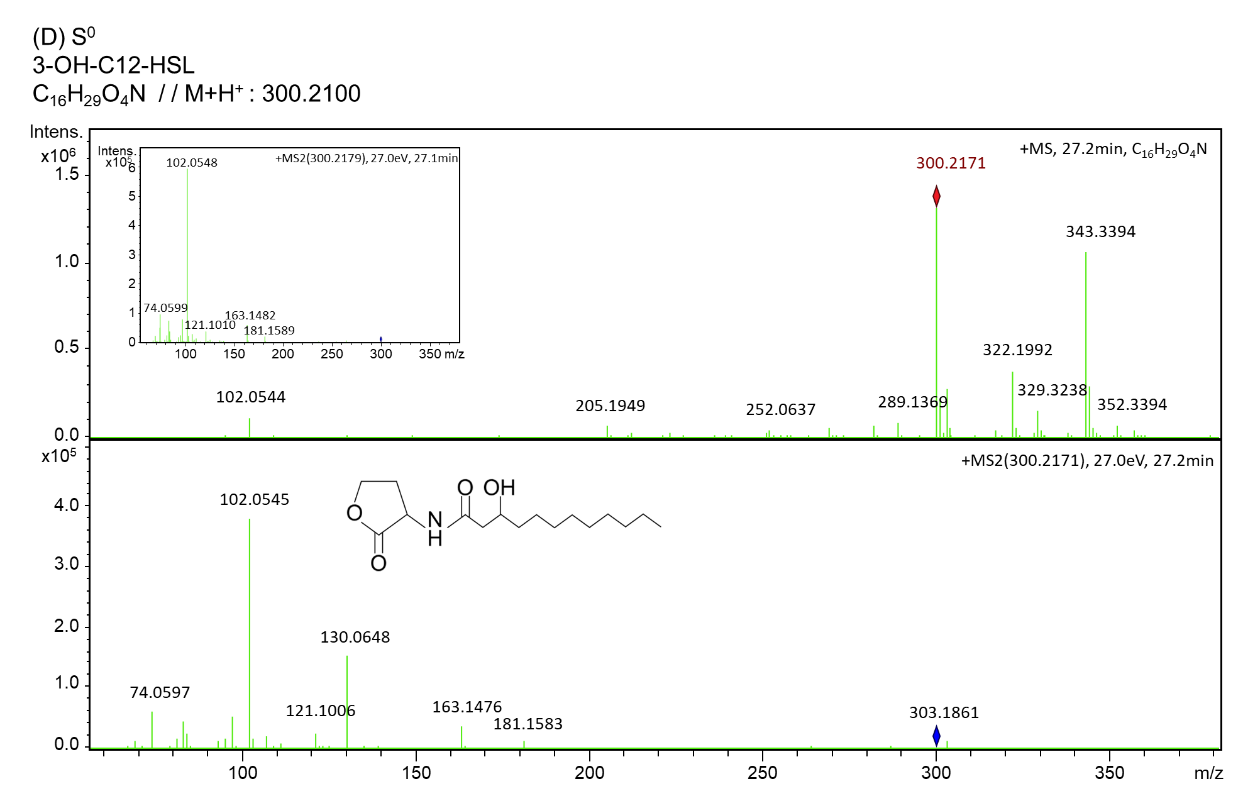
**

**
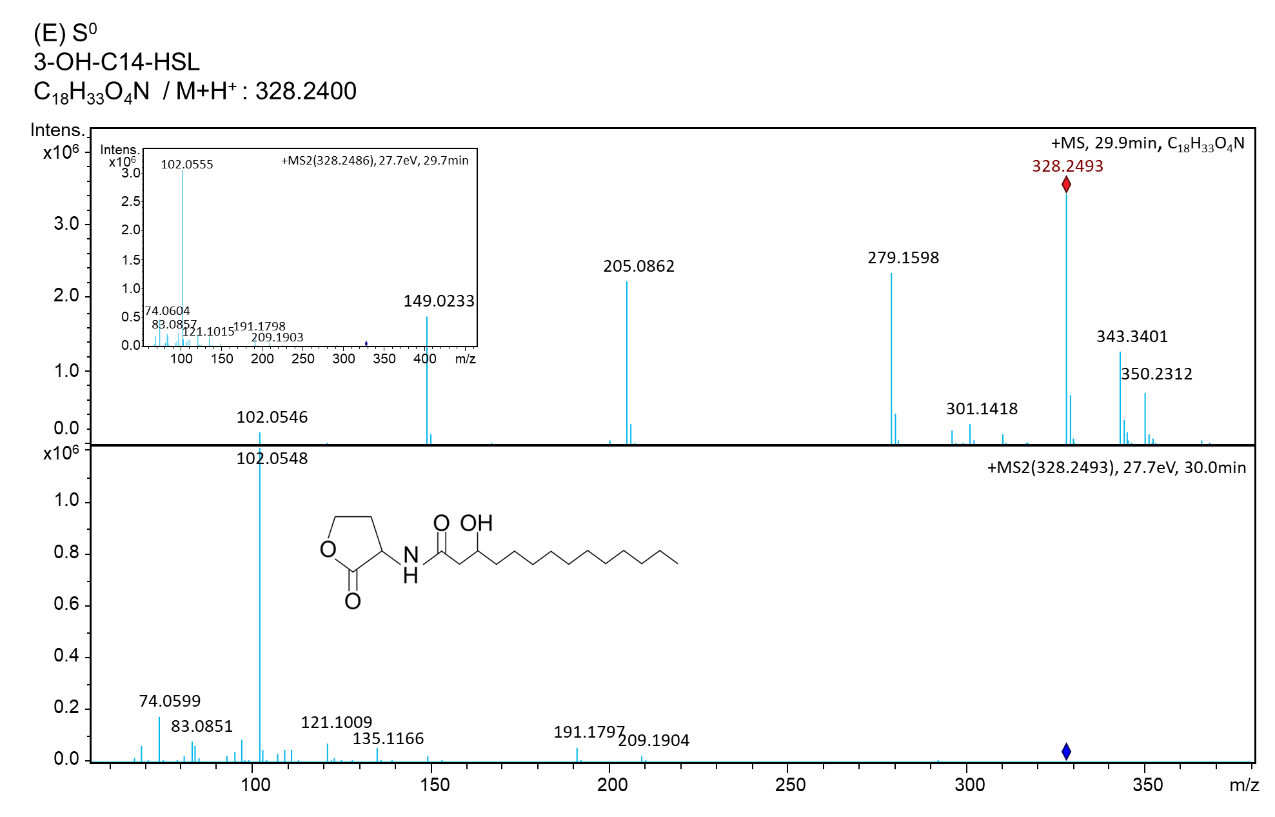
**

**
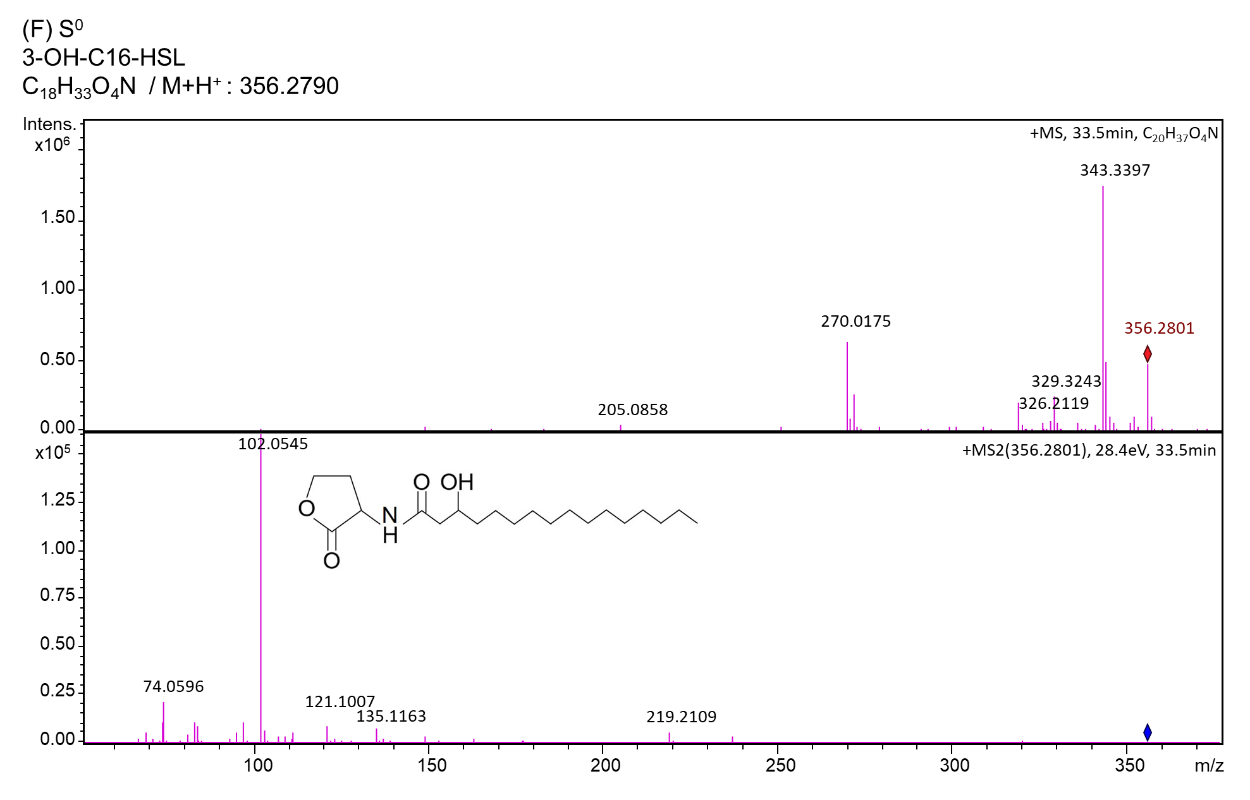
**

**
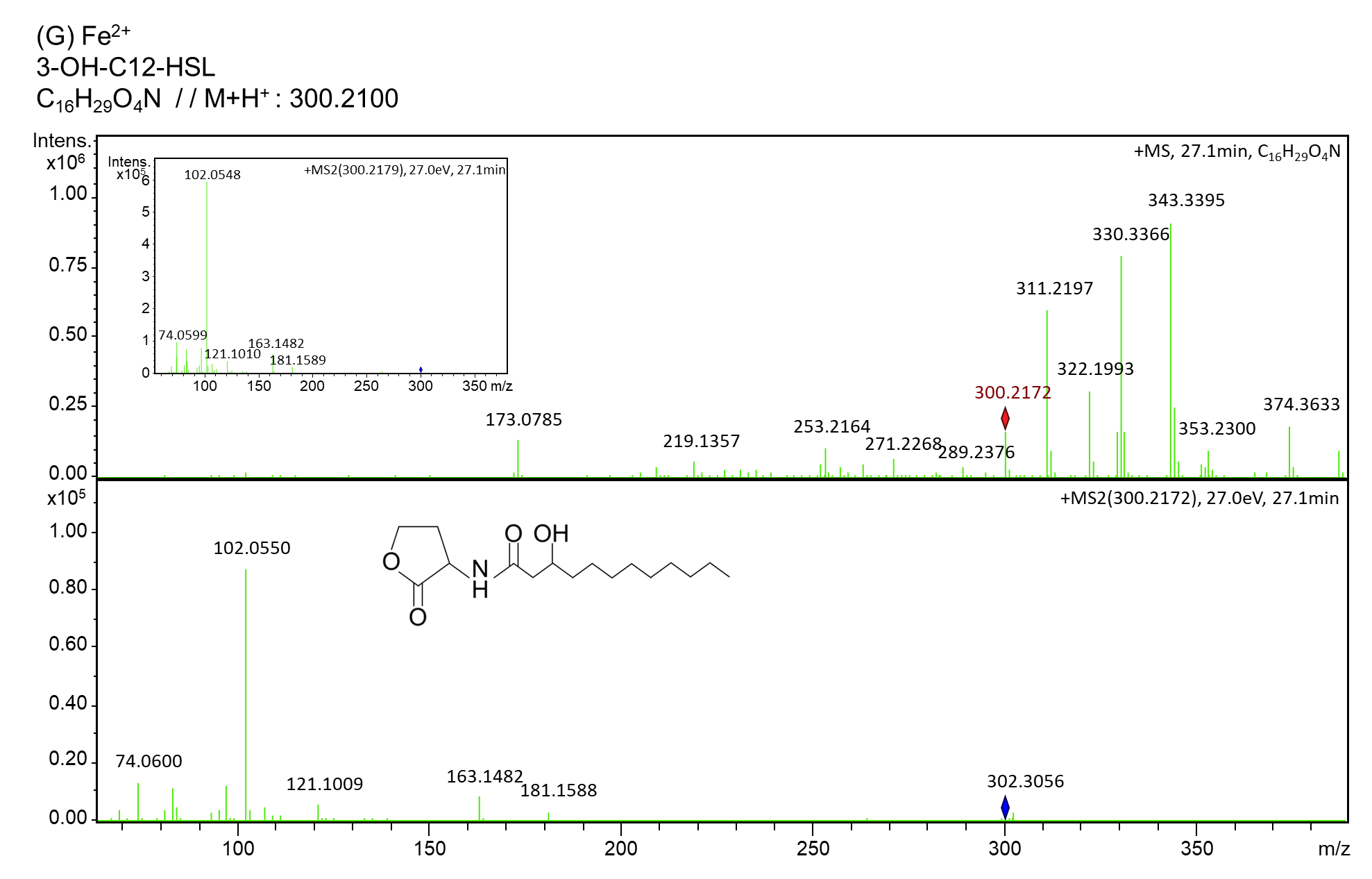
**

**
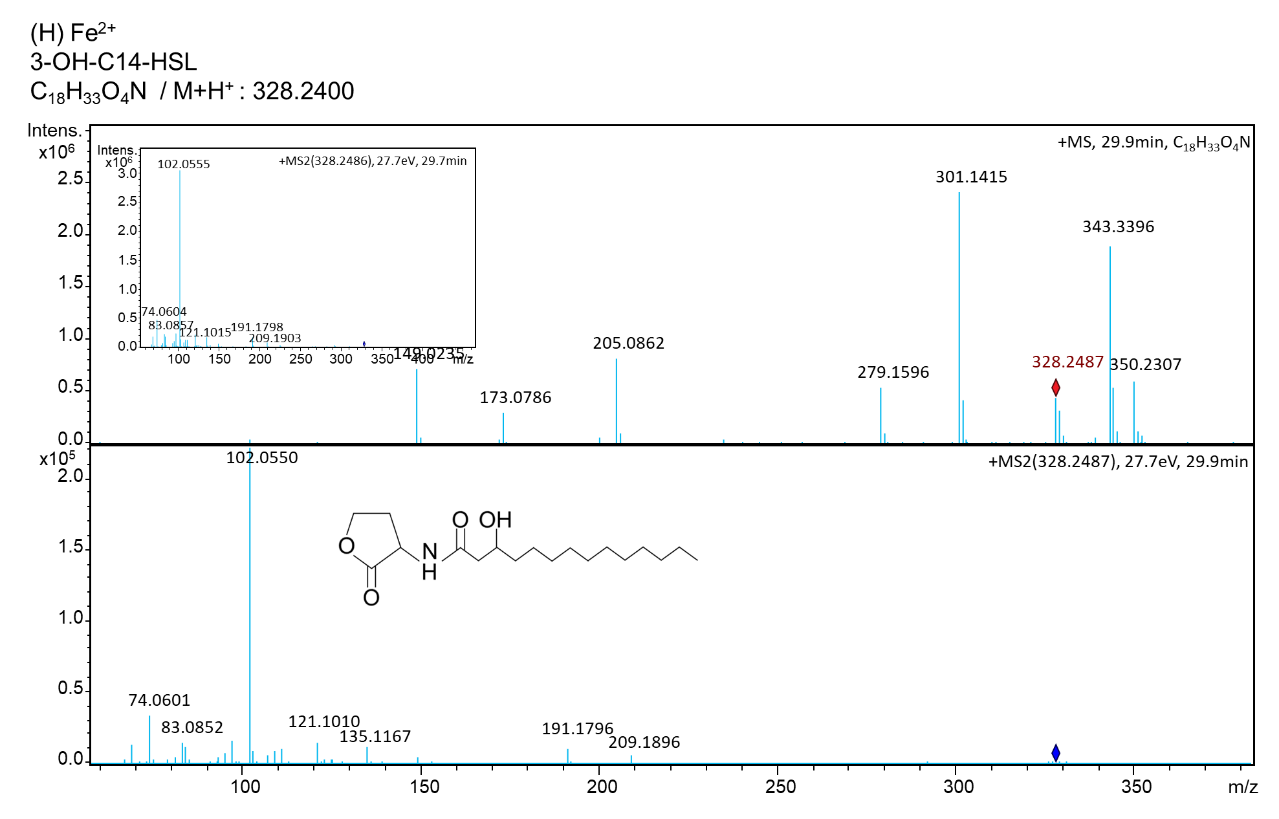
**

**
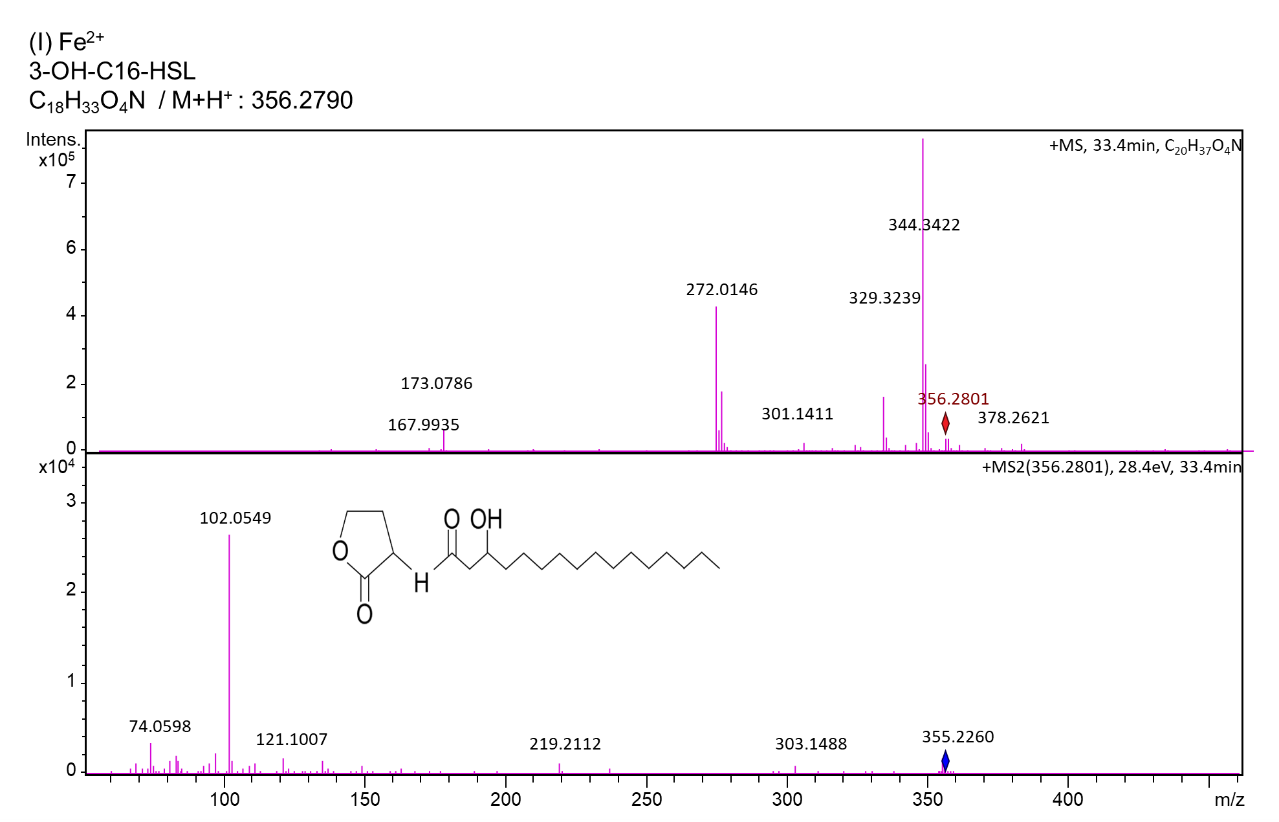
**

**Fig. S1 LC-MS-MS chromatograms and the structures of acyl-HSLs produced by AfeI in S^0-^ or Fe^2+^-enriched media.**

**
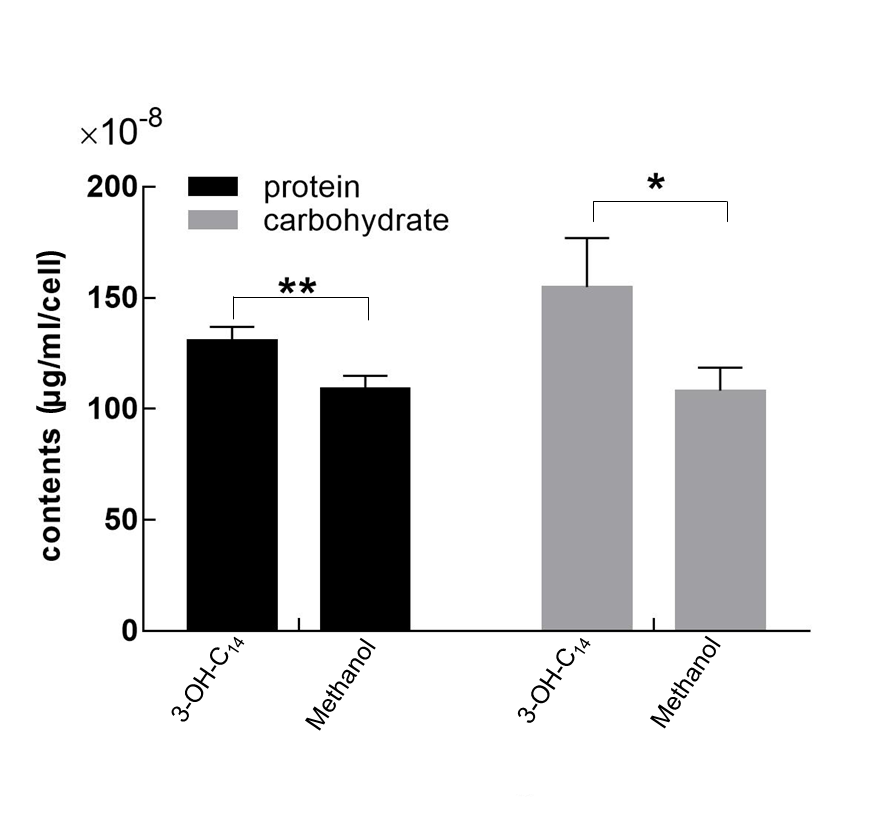
**

**Fig. S2 Analysis of EPS synthesis with the 10 μM 3-OH-C_14_-HSL addition to wide type.**

**
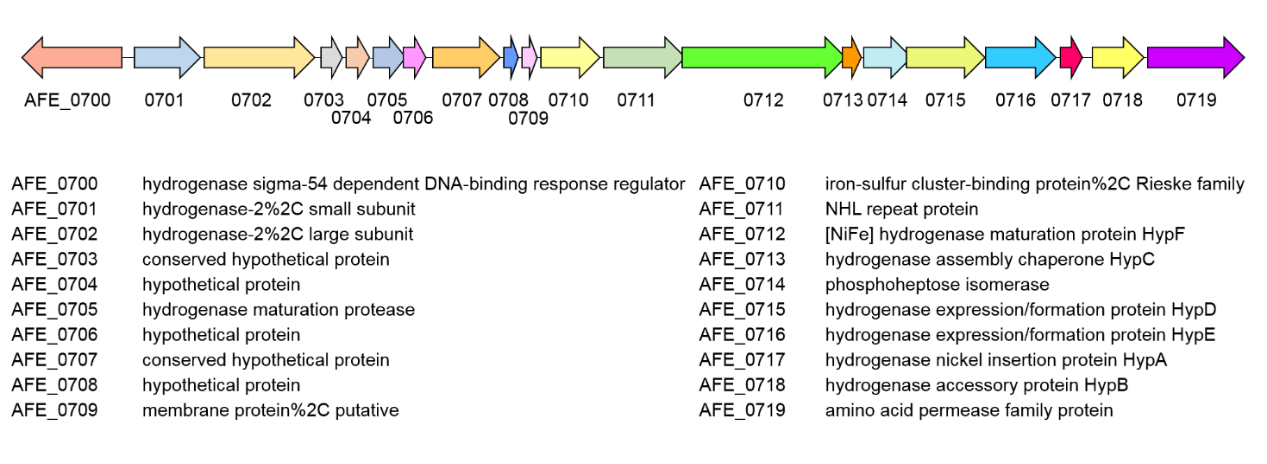
**

**Fig. S3 Diagram of the *hupR* contained hydrogenase gene cluster.**


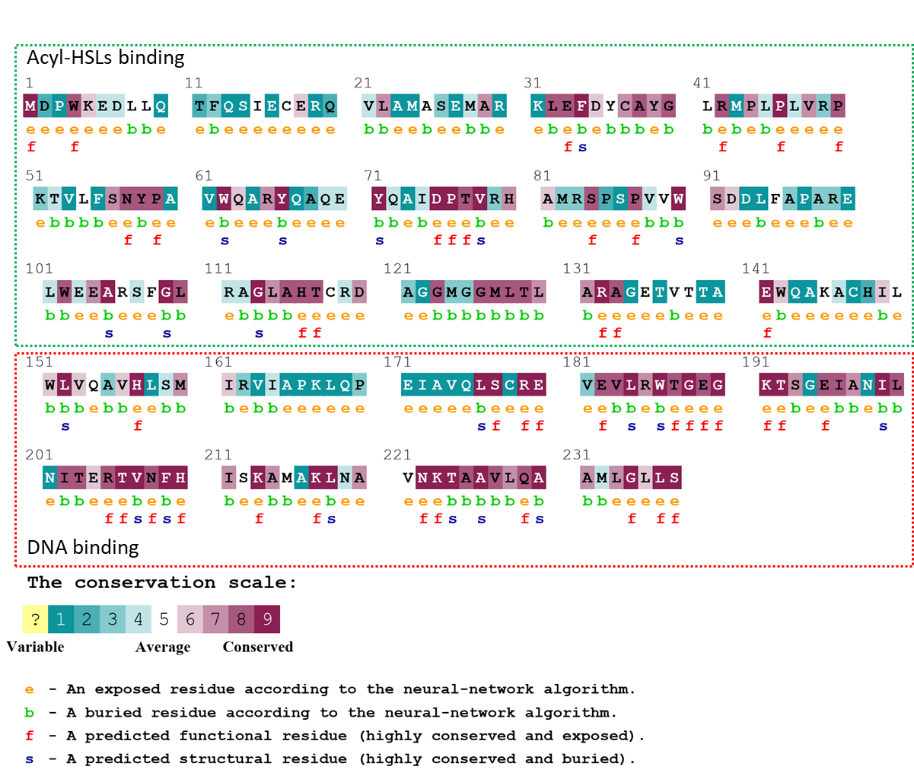


**Fig. S4 Sites conservation analysis of AfeR protein of *A. ferrooxidans*.** The Consurf software (<https://consurf.tau.ac.il/>) was used to analyze the conservation of AfeR protein sequences. The query sequence colored according to the conservation scores. Cyan represents low-conservative amino acids, and brown represents high-conservative amino acids. The sequences in the green dotted frame may recognize and bind signal molecules, and the sequences in the red dotted frame may bind DNA.

**Table S1 List of differentially expressed genes detected by RNA-seq.**

| Gene ID | RNA-seq (Fold change) | RT-qPCR(Fold change) | Description |
| --- | --- | --- | --- |
| Sulfur ---Δ*afeI* vs WT | | | |
| AFE_0017 | 2.04 | 2.40±1.50 | DNA processing protein DprA%2C putative |
| AFE_0049 | 2.28 | 2.14±0.21 | periplasmic solute-binding protein%2C putative |
| AFE_1982 | 2.01 |  | hypothetical protein |
| AFE_1991 | 2.01 | 1.50±0.26 | conserved hypothetical protein |
| AFE_2040 | 3.21 | 13.24±1.07 | TonB-dependent receptor |
| AFE_2268 | 2.07 | 2.95±0.45 | TonB family protein |
| AFE_2270 | 2.15 | 3.50±0.48 | MotA/TolQ/ExbB proton channel family protein |
| AFE_2301 | 2.48 | 1.81±0.04 | TonB family protein |
| AFE_2410 | 3.19 | 72.2±2.51 | acetyltransferase%2C GNAT family |
| AFE_2695 | 2.03 |  | hypothetical protein |
| AFE_2698 | 2.34 |  | hypothetical protein |
| AFE_3162 | 2.15 | 1.56±0.12 | acetyltransferase%2C GNAT family |
| AFE_3166 | 2.17 | 2.97±0.17 | acetyltransferase%2C GNAT family |
| AFE_3178 | 2.96 |  | hypothetical protein |
| AFE_3181 | 2.40 |  | - |
| AFE_3184 | 2.34 |  | - |
| AFE_3185 | 2.63 |  | - |
| AFE_3186 | 2.11 | 3.07±0.37 | Mrr restriction system protein |
| AFE_3187 | 3.54 | 10.72±8.24 | transposase. degenerate |
| AFE_3188 | 2.10 |  | hypothetical protein |
| AFE_3189 | 2.55 |  | conserved domain protein |
| AFE_3192 | 2.07 | 2.93±0.17 | type III restriction-modification system%2C Mod subunit |
| AFE_3194 | 2.01 | 1.88±0.87 | ATP-dependent DNA helicase RecG domain protein |
| AFE_3195 | 2.32 | 2.87±0.11 | Tn5468%2C orf5 |
| AFE_3197 | 2.19 |  | - |
| AFE_3198 | 2.32 |  | - |
| AFE_3234 | 2.00 |  | hypothetical protein |
| AFE_0095 | 0.47 |  | hypothetical protein |
| AFE_0219 | 0.40 |  | hypothetical protein |
| AFE_0267 | 0.48 |  | sulfide quinone reductase%2C putative |
| AFE_0294 | 0.50 |  | hypothetical protein |
| AFE_0302 | 0.44 |  | conserved domain protein |
| AFE_0466 | 0.50 |  | glutamine synthetase%2C type I |
| AFE_0590 | 0.44 |  | cold-shock protein |
| AFE_0707 | 0.31 | 0.42±0.10 | conserved hypothetical protein |
| AFE_0708 | 0.32 | 0.40±0.05 | hypothetical protein |
| AFE_0710 | 0.44 |  | iron-sulfur cluster-binding protein%2C Rieske family |
| AFE_0711 | 0.43 |  | NHL repeat protein |
| AFE_0714 | 0.47 |  | phosphoheptose isomerase |
| AFE_0728 | 0.49 |  | uroporphyrinogen decarboxylase |
| AFE_0729 | 0.45 |  | glutamate synthase%2C small subunit |
| AFE_0810 | 0.46 |  | oxidoreductase%2C iron-sulfur subunit |
| AFE_0858 | 0.45 |  | conserved hypothetical protein |
| AFE_1433 | 0.47 |  | ATP-dependent RNA helicase RhlE |
| AFE_1434 | 0.42 | 0.33±0.07 | DNA-binding response regulator PhoB |
| AFE_1814 | 0.49 |  | alkylhydroperoxidase AhpD domain protein |
| AFE_1830 | 0.33 | 0.45±0.08 | toluene tolerance protein%2C putative |
| AFE_1864 | 0.37 |  | hypothetical protein |
| AFE_1874 | 0.46 |  | adenylate cyclase |
| AFE_1875 | 0.44 |  | ParA-like protein |
| AFE_1884 | 0.22 | 0.25±0.17 | RNA chaperone Hfq |
| AFE_1886 | 0.42 | 0.27±0.02 | hflK protein |
| AFE_1887 | 0.46 |  | hflC protein |
| AFE_1889 | 0.45 |  | adenylosuccinate synthetase |
| AFE_1898 | 0.50 |  | superoxide dismutase |
| AFE_1951 | 0.44 |  | conserved hypothetical protein TIGR00481 |
| AFE_1998 | 0.22 | 0.08±0.03 | hypothetical protein |
| AFE_1999 | 0.00 |  | autoinducer synthesis protein |
| AFE_2062 | 0.46 |  | CBS domain protein |
| AFE_2083 | 0.37 | 0.25±0.05 | glycosyl transferase%2C group 1 |
| AFE_2084 | 0.41 |  | conserved hypothetical protein |
| AFE_2085 | 0.07 |  | conserved hypothetical protein |
| AFE_2086 | 0.06 | 0.13±0.02 | heat shock protein%2C Hsp20 family |
| AFE_2088 | 0.30 | 0.21±0.03 | zinc-binding alcohol dehydrogenase family protein |
| AFE_2089 | 0.47 |  | hypothetical protein |
| AFE_2209 | 0.39 | 0.38±0.07 | transcriptional regulator%2C TetR family |
| AFE_2267 | 0.41 | 0.07±0.01 | phosphoesterase family protein |
| AFE_2324 | 0.43 | 0.35±0.02 | phosphoglucomutase%2C putative |
| AFE_2325 | 0.36 |  | hypothetical protein |
| AFE_2518 | 0.50 |  | hypothetical protein |
| AFE_2653 | 0.43 |  | hypothetical protein |
| AFE_2665 | 0.49 |  | chaperone protein Dnak |
| AFE_2778 | 0.47 |  | hypothetical protein |
| AFE_2834 | 0.29 | 0.26±0.1 | SPFH/Band 7 domain protein |
| AFE_2838 | 0.44 | 0.33±0.11 | glucose-1-phosphate adenylyltransferase |
| AFE_2840 | 0.47 |  | glycosyl hydrolase%2C family 57 |
| AFE_3038 | 0.45 |  | glutaredoxin-related protein |
| Sulfur ---*afeI overexpression vs* WT(pJRD215) | | | |
| AFE_0111 | 2.11 |  | hypothetical protein |
| AFE_0118 | 2.36 |  | hypothetical protein |
| AFE_0146 | 2.47 |  | hypothetical protein |
| AFE_0149 | 2.45 |  | hypothetical protein |
| AFE_0171 | 2.07 | 2.11±0.46 | drug resistance transporter%2Cputative%2C EmrB/QacA |
| AFE_0307 | 2.22 |  | hypothetical protein |
| AFE_0426 | 2.29 |  | conserved domain protein |
| AFE_0494 | 2.45 |  |  |
| AFE_0502 | 2.84 |  | conserved hypothetical protein |
| AFE_0537 | 2.10 |  | PAP2 family protein |
| AFE_0541 | 2.08 |  | hypothetical protein |
| AFE_0581 | 2.24 |  | hypothetical protein |
| AFE_0614 | 2.13 |  | conserved hypothetical protein |
| AFE_0755 | 2.17 |  | hypothetical protein |
| AFE_0763 | 2.40 |  | TonB-dependent receptor%2C putative |
| AFE_0769 | 2.11 |  | biopolymer transport protein%2C ExbD/TolR family |
| AFE_0770 | 2.57 |  | TonB family protein |
| AFE_0771 | 2.04 |  | cytosine/purines/uracil/thiamine/allantoin permease family |
| AFE_0805 | 2.11 |  | auxin-responsive GH3-related protein |
| AFE_0806 | 2.06 |  | penicillin amidase family protein |
| AFE_0807 | 2.65 |  | hypothetical protein |
| AFE_0823 | 2.10 |  | hypothetical protein |
| AFE_0879 | 2.18 |  | transcriptional regulator%2C LysR family |
| AFE_0888 | 6.76 |  | conserved hypothetical protein |
| AFE_0964 | 2.27 |  | membrane protein%2C putative |
| AFE_1426 | 3.05 |  | hypothetical protein |
| AFE_1429 | 2.12 |  | cytochrome c552%2C putative |
| AFE_1500 | 2.50 |  | hypothetical protein |
| AFE_1511 | 2.18 |  | nitrogen fixation protein NifQ |
| AFE_1517 | 2.12 |  | nitrogenase MoFe cofactor biosynthesis protein NifE |
| AFE_1518 | 4.76 |  | ferredoxin%2C putative |
| AFE_1553 | 2.14 |  |  |
| AFE_1555 | 3.29 |  | tRNA-Val |
| AFE_1768 | 3.05 |  | hypothetical protein |
| AFE_1835 | 3.99 |  | hypothetical protein |
| AFE_1848 | 2.04 |  | base excision repair protein%2C HhH-GPD family |
| AFE_1860 | 2.13 |  | hypothetical protein |
| AFE_1928 | 2.07 |  | tRNA delta(2)-isopentenylpyrophosphate transferase |
| AFE_1948 | 2.26 |  | heavy metal efflux transporter%2C MFP subunit%2C putative |
| AFE_1949 | 2.40 | 1.93±0.40 | outer membrane heavy metal efflux protein%2C putative |
| AFE_1999 | 2.30 | 4.13±1.72 | autoinducer synthesis protein |
| AFE_2013 | 2.10 |  | transcriptional regulator%2C MarR family |
| AFE_2049 | 2.05 |  | hypothetical protein |
| AFE_2050 | 2.43 |  | hypothetical protein |
| AFE_2090 | 2.18 |  | peptidase%2C M48 family |
| AFE_2097 | 3.36 |  | hypothetical protein |
| AFE_2168 | 2.85 |  | hypothetical protein |
| AFE_2183 | 2.49 |  | universal stress family protein |
| AFE_2185 | 2.88 |  | transcriptional regulator%2C MarR family |
| AFE_2191 | 3.52 |  | hypothetical protein |
| AFE_2193 | 2.15 | 3.29±083 | bis(5-nucleosyl)-tetraphosphatase (symmetrical) |
| AFE_2197 | 2.12 |  | hypothetical protein |
| AFE_2213 | 2.66 |  | tRNA-Leu |
| AFE_2250 | 2.07 | 1.58±1.22 | carbohydrate-selective porin%2C OprB family |
| AFE_2289 | 2.20 |  |  |
| AFE_2293 | 2.61 |  | hypothetical protein |
| AFE_2303 | 2.29 |  | conserved hypothetical protein |
| AFE_2305 | 3.15 |  | hypothetical protein |
| AFE_2311 | 3.31 |  | hypothetical protein |
| AFE_2372 | 3.61 | 7.58±1.34 | type I restriction-modification system%2C M subunit |
| AFE_2382 | 2.12 |  | hypothetical protein |
| AFE_2396 | 2.21 |  | hypothetical protein |
| AFE_2410 | 2.40 |  | acetyltransferase%2C GNAT family |
| AFE_2491 | 2.11 |  | lipoprotein%2C putative |
| AFE_2501 | 2.17 |  | hypothetical protein |
| AFE_2507 | 2.38 |  |  |
| AFE_2668 | 2.02 |  | hypothetical protein |
| AFE_2721 | 2.18 |  | lipoate-protein ligase A%2C putative |
| AFE_2787 | 2.01 |  | hypothetical protein |
| AFE_2837 | 3.08 |  | hypothetical protein |
| AFE_2875 | 2.54 |  | transcriptional regulator%2C LysR family |
| AFE_2878 | 3.34 |  | hypothetical protein |
| AFE_2879 | 2.23 |  | conserved hypothetical protein |
| AFE_2889 | 2.25 |  | transcriptional regulator%2C AbrB family |
| AFE_2963 | 2.02 |  | capsule polysaccharide exporter%2C ATP-binding protein |
| AFE_2969 | 2.19 |  | hypothetical protein |
| AFE_2970 | 2.16 |  |  |
| AFE_2971 | 2.10 |  | sulfate adenylate transferase%2C large subunit/adenylylsulfate kinase |
| AFE_2997 | 2.33 |  | hypothetical protein |
| AFE_3105 | 2.29 |  | ISAfe6%2C transposase%2C degenerate |
| AFE_3162 | 3.01 | 2.48±0.20 | acetyltransferase%2C GNAT family |
| AFE_3163 | 2.69 | 2.04±0.11 | isochorismate pyruvate lyase%2C putative |
| AFE_3164 | 3.12 | 2.42±0.44 | ABC transporter%2C ATP-binding protein |
| AFE_3165 | 2.58 | 2.64±0.50 | membrane protein%2C putative |
| AFE_3166 | 2.45 | 2.14±0.43 | acetyltransferase%2C GNAT family |
| AFE_3175 | 3.08 |  | hypothetical protein |
| AFE_3181 | 2.39 |  |  |
| AFE_3182 | 2.57 |  |  |
| AFE_3184 | 2.66 |  |  |
| AFE_3185 | 4.15 |  |  |
| AFE_3186 | 2.07 | 2.01±0.35 | Mrr restriction system protein |
| AFE_3187 | 2.70 | 1.83±0.19 | transposase. degenerate |
| AFE_3188 | 4.13 |  | hypothetical protein |
| AFE_3189 | 2.60 |  | conserved domain protein |
| AFE_3191 | 2.11 | 1.92±0.11 | type III restriction-modification system%2C Res subunit |
| AFE_3192 | 3.01 | 1.77±0.14 | type III restriction-modification system%2C Mod subunit |
| AFE_3193 | 2.96 |  | PHP domain protein |
| AFE_3195 | 2.62 | 1.76±0.25 | Tn5468%2C orf5 |
| AFE_3272 | 2.76 |  | hypothetical protein |
| AFE_3290 | 2.13 |  |  |
| AFE_0154 | 0.38 |  | MIP family channel protein |
| AFE_0157 | 0.45 |  | s-adenosylmethionine decarboxylase proenzyme |
| AFE_0223 | 0.47 |  | UDP-3-0-acyl N-acetylglucosamine deacetylase |
| AFE_0253 | 0.47 |  | conserved domain protein |
| AFE_0317 | 0.45 |  | ribosomal protein L10 |
| AFE_0322 | 0.42 |  | ribosomal protein S12 |
| AFE_0334 | 0.44 | 0.01±0.002 | ribosomal protein L16 |
| AFE_0348 | 0.47 |  | adenylate kinase |
| AFE_0367 | 0.41 |  | AhpC/TSA family/glutaredoxin domain protein |
| AFE_0572 | 0.48 |  | conserved hypothetical protein |
| AFE_0574 | 0.46 |  | radical SAM domain protein |
| AFE_0659 | 0.40 |  | ribosomal protein L31 |
| AFE_0960 | 0.42 |  | periplasmic glucans biosynthesis protein%2C MdoG |
| AFE_0961 | 0.44 |  | hypothetical protein |
| AFE_1565 | 0.00 |  | hypothetical protein |
| AFE_1566 | 0.01 | 0.001±0.0001 | DNA replication protein DnaC%2C putative |
| AFE_1567 | 0.06 |  | hypothetical protein |
| AFE_1568 | 0.02 |  | methyltransferase%2C putative |
| AFE_1579 | 0.28 |  | conserved hypothetical protein |
| AFE_1582 | 0.45 |  | hypothetical protein |
| AFE_1584 | 0.22 | 0.28±0.04 | DNA-binding protein HU family protein |
| AFE_1585 | 0.39 |  | hypothetical protein |
| AFE_1590 | 0.21 |  | conserved hypothetical protein |
| AFE_1591 | 0.07 |  | conserved domain protein |
| AFE_1614 | 0.33 | 0.09±0.01 | excisionase domain protein |
| AFE_1615 | 0.17 | 0.002±0.001 | DNA primase TraC |
| AFE_1616 | 0.14 |  | hypothetical protein |
| AFE_1617 | 0.33 |  | hypothetical protein |
| AFE_1618 | 0.02 |  | hypothetical protein |
| AFE_1619 | 0.06 |  | hypothetical protein |
| AFE_1620 | 0.40 |  | hypothetical protein |
| AFE_1621 | 0.21 |  | conserved hypothetical protein |
| AFE_1624 | 0.41 |  | hypothetical protein |
| AFE_1625 | 0.45 |  | hypothetical protein |
| AFE_1634 | 0.05 | 0.01±0.01 | P-type conjugative transfer protein TrbB |
| AFE_1636 | 0.01 |  | hypothetical protein |
| AFE_1637 | 0.01 | 0.001±0.0002 | conjugal transfer protein TrbD%2C putative |
| AFE_1638 | 0.02 | 0.003±0.003 | conjugal transfer protein trbE%2C putative |
| AFE_1639 | 0.03 | 0.01±0.003 | hypothetical protein |
| AFE_1640 | 0.01 | 0.01±0.001 | conjugal transfer protein trbJ%2C putative |
| AFE_1641 | 0.01 | 0.01±0.001 | lipoprotein%2C putative |
| AFE_1642 | 0.12 | 0.01±0.007 | conjugal transfer protein trbL%2C putative |
| AFE_1643 | 0.39 |  | hypothetical protein |
| AFE_1647 | 0.28 |  | hypothetical protein |
| AFE_1651 | 0.33 | 0.53±0.12 | 3-hydroxyisobutyrate dehydrogenase family protein |
| AFE_1693 | 0.46 |  | conserved hypothetical protein |
| AFE_1694 | 0.02 | 0.002±0.001 | conjugal transfer protein |
| AFE_1695 | 0.09 | 0.01±0.001 | conjugal transfer protein |
| AFE_1696 | 0.18 | 0.06±0.02 | conjugal transfer protein%2C putative |
| AFE_1697 | 0.16 |  |  |
| AFE_1699 | 0.23 | 0.03±0.003 | TraG/TraD family protein |
| AFE_1701 | 0.27 |  |  |
| AFE_1716 | 0.49 |  | GTP-binding protein |
| AFE_1717 | 0.40 |  | hypothetical protein |
| AFE_1719 | 0.26 | 0.06±0.01 | parB family protein |
| AFE_1720 | 0.15 | 0.03±0.001 | parA family protein |
| AFE_1721 | 0.17 |  | hypothetical protein |
| AFE_1726 | 0.18 | 0.06±0.01 | conjugal transfer protein TraF%2C putative |
| AFE_1730 | 0.10 |  | hypothetical protein |
| AFE_1731 | 0.26 | 0.05±0.01 | conjugal transfer protein TraB%2C putative |
| AFE_1733 | 0.35 |  | conserved hypothetical protein |
| AFE_1734 | 0.29 |  | conserved hypothetical protein |
| AFE_1737 | 0.03 |  | hypothetical protein |
| AFE_1738 | 0.22 | 0.02±0.002 | DNA topoisomerase III |
| AFE_1864 | 0.27 |  | hypothetical protein |
| AFE_1868 | 0.26 | 0.34±0.03 | LexA repressor |
| AFE_1904 | 0.43 |  | ribosomal protein L32 |
| AFE_1940 | 0.34 | 0.06±0.02 | phosphate ABC transporter%2C permease protein PstC |
| AFE_2177 | 0.35 | 0.3±0.23 | sulfur reductase%2C molybdopterin subunit |
| AFE_2188 | 0.47 |  | ribosomal protein S20 |
| AFE_2364 | 0.47 |  | rhodanese-like domain protein |
| AFE_2368 | 0.47 |  | outer membrane efflux protein |
| AFE_2611 | 0.45 |  | ribosomal protein L20 |
| AFE_2822 | 0.34 |  | hypothetical protein |
| AFE_2824 | 0.43 |  | ribosomal protein S6 |
| AFE_2855 | 0.37 |  | ribosomal protein S16 |
| AFE_2891 | 0.44 |  | conserved hypothetical protein |
| AFE_3038 | 0.46 |  | glutaredoxin-related protein |
| AFE_3075 | 0.44 |  | ribosomal protein L13 |
| AFE_3208 | 0.48 |  | ATP synthase F0%2C C subunit |
| Ferrous ---Δ*afeI* vs WT | | | |
| AFE_0012 | 2.93 |  | septum site-determining protein MinD |
| AFE_0015 | 2.51 |  | DNA topoisomerase I |
| AFE_0016 | 2.78 |  | smg family protein |
| AFE_0029 | 2.80 |  | tetrathionate hydrolase |
| AFE_0040 | 2.47 |  | drug resistance transporter%2C EmrB/QacA family |
| AFE_0057 | 2.19 |  | quinolinate synthetase complex%2C subunit A |
| AFE_0059 | 2.05 |  | conserved hypothetical protein |
| AFE_0075 | 2.14 |  | conserved hypothetical protein |
| AFE_0092 | 2.46 |  | fic family protein |
| AFE_0095 | 2.42 |  | hypothetical protein |
| AFE_0115 | 2.42 |  | conserved domain protein |
| AFE_0122 | 2.21 |  | conserved domain protein |
| AFE_0134 | 2.27 |  | oxygen-independent coproporphyrinogen III oxidase |
| AFE_0135 | 3.26 |  | transcriptional regulator%2C LysR family |
| AFE_0140 | 2.67 |  | oxidase%2C FAD-binding |
| AFE_0141 | 2.79 |  | phosphoheptose isomerase/cytidylyltransferase |
| AFE_0158 | 2.28 |  | toluene ABC transporter%2C ATP-binding protein%2C putative |
| AFE_0166 | 2.21 |  | HNH endonuclease domain protein |
| AFE_0177 | 2.64 |  | glutathione S-transferase family protein |
| AFE_0188 | 2.70 |  | conserved hypothetical protein |
| AFE_0190 | 3.11 |  | stringent starvation protein B |
| AFE_0200 | 3.61 |  | cell division protein FtsZ |
| AFE_0201 | 6.16 | 1.64±0.17 | cell division protein FtsA |
| AFE_0202 | 5.35 | 2.83±0.50 | cell division protein FtsQ |
| AFE_0203 | 5.37 | 2.87±0.32 | D-alanine--D-alanine ligase |
| AFE_0204 | 4.53 | 2.52±0.17 | UDP-N-acetylenolpyruvoylglucosamine reductase |
| AFE_0205 | 3.46 |  | UDP-N-acetylmuramate--alanine ligase |
| AFE_0206 | 3.52 |  | UDP-N-acetylglucosamine--N-acetylmuramyl-(pentapeptide) pyrophosphoryl-undecaprenol N-acetylglucosamine transferase |
| AFE_0207 | 3.88 |  | cell division protein FtsW |
| AFE_0208 | 3.80 |  | UDP-N-acetylmuramoylalanine--D-glutamate ligase |
| AFE_0209 | 2.71 |  | phospho-N-acetylmuramoyl-pentapeptide-transferase |
| AFE_0210 | 3.53 |  | UDP-N-acetylmuramoyl-tripeptide--D-alanyl-D-alanine ligase |
| AFE_0211 | 2.26 |  | UDP-N-acetylmuramoylalanyl-D-glutamate--2%2C6-diaminopimelate ligase |
| AFE_0212 | 2.12 |  | peptidoglycan synthetase FtsI |
| AFE_0213 | 4.61 |  | cell division protein FtsL |
| AFE_0236 | 2.68 |  | conserved hypothetical protein |
| AFE_0239 | 2.06 |  | aminotransferase%2C class II |
| AFE_0240 | 2.40 |  | conserved hypothetical protein |
| AFE_0243 | 2.34 |  | 8-amino-7-oxononanoate synthase |
| AFE_0244 | 2.98 |  | bioH protein |
| AFE_0245 | 2.11 |  | biotin synthesis protein BioC |
| AFE_0247 | 2.52 |  |  |
| AFE_0253 | 2.11 |  | conserved domain protein |
| AFE_0259 | 2.43 |  | biotin synthase |
| AFE_0278 | 2.40 |  | phospholipase D family protein |
| AFE_0284 | 3.47 |  | ribosomal protein L27 |
| AFE_0355 | 2.61 |  | ribosomal protein L17 |
| AFE_0364 | 2.22 |  | hypoxanthine-guanine phosphoribosyltransferase%2C putative |
| AFE_0366 | 3.30 |  | glutathione reductase |
| AFE_0367 | 3.07 |  | AhpC/TSA family/glutaredoxin domain protein |
| AFE_0373 | 3.08 |  | transcriptional regulator%2C MerR family |
| AFE_0376 | 3.37 |  |  |
| AFE_0419 | 2.36 |  | transaldolase |
| AFE_0423 | 3.75 |  | aconitate hydratase%2C putative |
| AFE_0424 | 3.38 |  | isocitrate dehydrogenase%2C NADP-dependent |
| AFE_0425 | 2.67 |  | succinyl-CoA synthase%2C beta subunit |
| AFE_0427 | 3.07 |  | succinyl-CoA synthetase%2C alpha subunit |
| AFE_0429 | 2.21 |  |  |
| AFE_0437 | 3.85 |  | ACT domain protein |
| AFE_0449 | 2.35 |  | sensor histidine kinase |
| AFE_0450 | 2.12 |  | DNA repair protein RecN |
| AFE_0451 | 2.68 |  | ATP-NAD kinase |
| AFE_0454 | 2.18 |  | gamma-glutamyl phosphate reductase |
| AFE_0468 | 2.48 |  | acid phosphatase SurE |
| AFE_0470 | 2.23 |  | RNA polymerase sigma-38 factor |
| AFE_0471 | 2.27 |  | conserved hypothetical protein |
| AFE_0527 | 2.14 |  | glycogen/starch/alpha-glucan phosphorylase |
| AFE_0536 | 3.28 |  | phosphoribulokinase |
| AFE_0539 | 2.09 |  | sulfate adenylyltransferase%2C putative/adenylylsulfate kinase |
| AFE_0540 | 2.20 |  | adenine phosphoribosyltransferase%2C putative |
| AFE_0548 | 2.23 |  | glutamate-ammonia-ligase adenylyltransferase |
| AFE_0551 | 2.95 |  | hydrolase%2C carbon-nitrogen family |
| AFE_0562 | 2.40 |  | yicC family protein |
| AFE_0565 | 2.29 |  | guanosine-3%2C5-bis(diphosphate) 3-pyrophosphohydrolase |
| AFE_0566 | 3.24 |  | endoribonuclease L-PSP%2C putative |
| AFE_0572 | 2.59 |  | conserved hypothetical protein |
| AFE_0573 | 2.69 |  | conserved hypothetical protein TIGR00296 |
| AFE_0587 | 2.27 |  | sodium/calcium exchanger |
| AFE_0590 | 6.03 |  | cold-shock protein |
| AFE_0595 | 2.36 |  | lipoprotein%2C putative |
| AFE_0596 | 2.93 |  | tRNA-i(6)A37 modification enzyme MiaB |
| AFE_0597 | 3.47 |  | PhoH-like protein |
| AFE_0609 | 2.42 |  | NAD-dependent epimerase/dehydratase family protein |
| AFE_0642 | 2.41 |  | thiamine biosynthesis protein ThiS |
| AFE_0702 | 2.02 |  | hydrogenase-2%2C large subunit |
| AFE_0732 | 4.14 | 2.58±0.32 | thymidylate synthase%2C flavin-dependent |
| AFE_0733 | 3.67 |  | 3-dehydroquinate synthase |
| AFE_0749 | 2.05 |  | pyruvate kinase barrel domain protein |
| AFE_0752 | 2.35 |  | GTP cyclohydrolase I family protein |
| AFE_0846 | 2.17 |  | alcohol dehydrogenase%2C zinc-containing |
| AFE_0848 | 2.09 |  | 3-deoxy-8-phosphooctulonate synthase |
| AFE_0852 | 2.80 |  | conserved hypothetical protein |
| AFE_0881 | 2.03 |  | conserved hypothetical protein |
| AFE_0882 | 2.60 |  | flavin monoamine oxidase family protein |
| AFE_0909 | 2.48 |  | trigger factor |
| AFE_0911 | 2.16 |  | ATP-dependent Clp protease%2C ATP-binding subunit ClpX |
| AFE_0912 | 2.10 |  |  |
| AFE_0914 | 2.17 |  | DNA-binding protein HU |
| AFE_0926 | 2.84 |  | sugar fermentation stimulation protein |
| AFE_0939 | 2.14 |  | [NiFe] hydrogenase%2C gamma subunit%2C putative |
| AFE_0941 | 3.02 |  | murein transglycosylase%2C putative |
| AFE_0951 | 2.60 |  | aldehyde dehydrogenase (NAD) family protein |
| AFE_0953 | 3.45 |  | conserved domain protein |
| AFE_0954 | 2.53 |  | cytochrome d ubiquinol oxidase%2C subunit II |
| AFE_0960 | 2.50 |  | periplasmic glucans biosynthesis protein%2C MdoG |
| AFE_0975 | 5.85 |  | molybdopterin converting factor%2C subunit 1 |
| AFE_0976 | 2.55 |  | molybdopterin converting factor%2C subunit 2 |
| AFE_0991 | 2.43 |  | tRNA (guanosine-2'-O-)-methyltransferase%2C putative |
| AFE_1388 | 2.23 |  | ABC transporter%2C CydDC cysteine exporter (CydDC-E) family%2C permease/ATP-binding protein CydD |
| AFE_1392 | 2.48 |  | L-aspartate oxidase |
| AFE_1450 | 2.31 |  | 1-deoxy-D-xylulose 5-phosphate reductoisomerase |
| AFE_1474 | 3.49 |  | glutathione S-transferase%2C putative |
| AFE_1508 | 2.26 |  | nitrogen fixation protein nifU |
| AFE_1546 | 2.04 |  | succinate-semialdehyde dehydrogenase%2C putative |
| AFE_1552 | 2.20 |  | sucrose synthase%2C putative |
| AFE_1667 | 2.60 |  | transketolase pyridine binding domain protein |
| AFE_1762 | 3.62 |  | conserved hypothetical protein |
| AFE_1771 | 2.17 |  | 3-methyl-2-oxobutanoate hydroxymethyltransferase |
| AFE_1780 | 2.98 |  | plasmid stability protein%2C putative |
| AFE_1785 | 2.16 |  | hypothetical protein |
| AFE_1826 | 2.10 |  | amidohydrolase family protein |
| AFE_1827 | 2.10 |  | membrane-bound lytic murein transglycosylase B%2C putative |
| AFE_1828 | 2.11 |  | lipoprotein%2C NLP/P60 family |
| AFE_1874 | 2.19 |  | adenylate cyclase |
| AFE_1881 | 2.08 |  | hypothetical protein |
| AFE_1887 | 2.31 |  | hflC protein |
| AFE_1897 | 2.08 |  | phosphoribosylformylglycinamidine cyclo-ligase |
| AFE_1900 | 3.17 |  | CDP-diacylglycerol--glycerol-3-phosphate 3-phosphatidyltransferase%2C putative |
| AFE_1901 | 2.16 |  | conserved hypothetical protein |
| AFE_1923 | 2.79 |  | YjeF-related protein |
| AFE_2023 | 2.33 |  | phosphoribosylglycinamide formyltransferase 2 |
| AFE_2028 | 2.13 |  | DNA topoisomerase IV%2C A subunit |
| AFE_2029 | 2.41 |  | DNA topoisomerase IV%2C subunit B |
| AFE_2032 | 5.76 | 2.00±0.20 | DNA-binding response regulator |
| AFE_2033 | 8.49 |  | conserved domain protein |
| AFE_2034 | 3.53 |  | conserved domain protein |
| AFE_2035 | 5.15 | 5.62±1.28 | transporter%2C AcrB/AcrD/AcrF family |
| AFE_2036 | 6.79 | 9.25±0.58 | efflux transporter%2C RND family%2C MFP subunit |
| AFE_2037 | 9.65 | 9.35±0.98 | outer membrane efflux protein |
| AFE_2064 | 2.40 |  | amidophosphoribosyltransferase |
| AFE_2067 | 2.58 |  | acetyl-CoA carboxylase%2C carboxyl transferase%2C beta subunit |
| AFE_2069 | 2.29 |  | tryptophan synthase%2C beta subunit |
| AFE_2070 | 2.31 |  | N-(5'phosphoribosyl)anthranilate isomerase |
| AFE_2076 | 2.45 |  | molybdopterin-guanine dinucleotide biosynthesis protein A |
| AFE_2079 | 2.22 |  | endonuclease |
| AFE_2134 | 3.31 |  | lipoprotein%2C putative |
| AFE_2135 | 2.55 |  | mce-related protein |
| AFE_2136 | 4.06 |  |  |
| AFE_2141 | 3.53 |  | 6S / SsrS RNA |
| AFE_2170 | 2.15 |  | polysaccharide deacetylase family protein |
| AFE_2171 | 2.23 |  | GTP-binding protein |
| AFE_2186 | 2.48 |  | prolipoprotein diacylglyceryl transferase |
| AFE_2188 | 2.97 |  | ribosomal protein S20 |
| AFE_2201 | 2.31 |  | cytosol aminopeptidase |
| AFE_2207 | 2.17 |  | hypothetical protein |
| AFE_2222 | 2.05 |  |  |
| AFE_2253 | 2.27 |  | conserved hypothetical protein |
| AFE_2260 | 2.31 |  | phosphoribosylaminoimidazolecarboxamide formyltransferase/IMP cyclohydrolase |
| AFE_2261 | 2.31 |  | phosphoribosylamine--glycine ligase |
| AFE_2270 | 2.59 |  | MotA/TolQ/ExbB proton channel family protein |
| AFE_2272 | 2.10 |  | hypothetical protein |
| AFE_2314 | 2.07 |  | phosphohistidine phosphatase SixA |
| AFE_2323 | 2.23 |  | conserved hypothetical protein |
| AFE_2351 | 2.54 |  | membrane protein%2C putative |
| AFE_2361 | 2.57 |  | N-acetylmuramoyl-L-alanine amidase%2C putative |
| AFE_2388 | 2.45 |  |  |
| AFE_2403 | 2.05 |  |  |
| AFE_2416 | 2.94 |  | hypothetical protein |
| AFE_2480 | 2.75 |  | mercuric resistance protein MerC |
| AFE_2481 | 2.43 |  | mercuric reductase |
| AFE_2504 | 2.06 |  | transposon%2C transposition helper protein C%2C putative |
| AFE_2528 | 3.16 |  | glutamine amidotransferase%2C class I |
| AFE_2531 | 2.32 |  | transcriptional regulator%2C LysR family |
| AFE_2547 | 2.41 |  | glycine cleavage system H protein%2C putative |
| AFE_2548 | 2.13 |  | conserved hypothetical protein |
| AFE_2578 | 2.51 |  | roadblock/LC7 domain protein |
| AFE_2596 | 2.67 |  |  |
| AFE_2597 | 2.05 |  | sigma-54 dependent transcriptional regulator%2C putative |
| AFE_2611 | 2.06 |  | ribosomal protein L20 |
| AFE_2619 | 2.07 |  | NADH-quinone oxidoreductase%2C L subunit |
| AFE_2637 | 2.20 |  | tldD protein |
| AFE_2641 | 2.19 |  | transcriptional regulator%2C ArsR family |
| AFE_2642 | 2.05 |  | membrane protein%2C putative |
| AFE_2659 | 2.01 |  | amino acid permease family protein |
| AFE_2661 | 2.43 |  | Mrp protein |
| AFE_2662 | 2.78 |  | antibiotic biosynthesis monooxygenase family protein |
| AFE_2677 | 2.12 |  | 3-deoxy-D-manno-octulosonic-acid transferase domain protein |
| AFE_2687 | 4.01 |  | hypothetical protein |
| AFE_2697 | 2.70 |  | conserved hypothetical protein |
| AFE_2702 | 3.50 |  | tight adherence protein TadA |
| AFE_2703 | 2.24 |  | pilus assembly protein CpaE%2C putative |
| AFE_2710 | 4.64 |  | pilin%2C putative |
| AFE_2725 | 2.02 |  |  |
| AFE_2738 | 2.44 |  | apaG protein |
| AFE_2743 | 3.77 |  | diaminopimelate epimerase |
| AFE_2745 | 3.09 |  | conserved hypothetical protein |
| AFE_2746 | 3.43 |  | tyrosine recombinase XerC |
| AFE_2747 | 2.56 |  | heat shock protein HslV |
| AFE_2748 | 2.34 |  | heat shock protein HslVU%2C ATPase subunit HslU |
| AFE_2751 | 2.11 |  | cell division protein FtsX%2C putative |
| AFE_2752 | 2.04 |  | cell division ATP-binding protein FtsE |
| AFE_2753 | 2.09 |  | signal recognition particle-docking protein FtsY |
| AFE_2779 | 2.02 |  | copper-translocating P-type ATPase |
| AFE_2793 | 2.08 |  | type I restriction-modification system%2C M subunit |
| AFE_2815 | 2.73 |  | Xaa-Pro aminopeptidase |
| AFE_2816 | 2.09 |  | hypothetical protein |
| AFE_2830 | 2.16 |  | conserved hypothetical protein |
| AFE_2836 | 2.25 |  | 1%2C4-alpha-glucan branching enzyme |
| AFE_2840 | 2.88 |  | glycosyl hydrolase%2C family 57 |
| AFE_2841 | 2.58 |  | ROK family protein |
| AFE_2845 | 2.41 |  |  |
| AFE_2847 | 2.34 |  | excinuclease ABC%2C A subunit |
| AFE_2855 | 3.66 |  | ribosomal protein S16 |
| AFE_2860 | 2.41 |  | arsenate reductase |
| AFE_2882 | 2.04 |  | uracil-DNA glycosylase family 4 protein |
| AFE_2886 | 2.84 |  | PemI family protein |
| AFE_2913 | 2.43 |  | SpoVR like family protein |
| AFE_2919 | 2.10 |  | lipoprotein%2C putative |
| AFE_2921 | 2.02 |  | rare lipoprotein A%2C putative |
| AFE_2928 | 2.06 |  | hypothetical protein |
| AFE_2933 | 2.06 |  | exodeoxyribonuclease V%2C alpha subunit |
| AFE_2949 | 2.72 |  | iojap-related protein |
| AFE_2957 | 2.29 |  | sensor histidine kinase |
| AFE_2975 | 2.48 |  | capsule polysaccharide export protein%2C BexD/CtrA/VexA family |
| AFE_3008 | 2.36 |  | glycosyl transferase%2C group 1 |
| AFE_3019 | 2.47 |  | phosphocarrier protein HPr |
| AFE_3032 | 3.11 |  | nucleotidyltransferase family protein |
| AFE_3040 | 2.21 |  | ATP phosphoribosyltransferase |
| AFE_3041 | 2.65 |  | histidinol dehydrogenase |
| AFE_3043 | 2.79 |  | imidazoleglycerol-phosphate dehydratase |
| AFE_3051 | 2.10 |  |  |
| AFE_3052 | 2.73 |  |  |
| AFE_3062 | 2.38 |  | conserved hypothetical protein |
| AFE_3064 | 2.13 |  | glutamate--cysteine ligase |
| AFE_3090 | 2.74 |  | membrane protein%2C putative |
| AFE_3092 | 4.56 |  | ribosome biogenesis GTP-binding protein YsxC |
| AFE_3102 | 2.39 |  | curved DNA-binding protein |
| AFE_3103 | 3.10 |  | phosphoribosyl transferase domain protein |
| AFE_3104 | 2.20 |  | DNA polymerase X family protein |
| AFE_3107 | 2.37 |  |  |
| AFE_3146 | 2.30 | 1.54±0.49 | rusticyanin |
| AFE_3164 | 3.77 |  | ABC transporter%2C ATP-binding protein |
| AFE_3165 | 4.80 | 1.59±0.14 | membrane protein%2C putative |
| AFE_3172 | 4.58 |  | glyoxalase family protein |
| AFE_3174 | 2.48 |  | prevent-host-death family protein |
| AFE_3181 | 3.40 |  |  |
| AFE_3186 | 3.24 |  | Mrr restriction system protein |
| AFE_3189 | 2.58 |  | conserved domain protein |
| AFE_3191 | 4.94 | 4.14±0.20 | type III restriction-modification system%2C Res subunit |
| AFE_3197 | 2.99 |  |  |
| AFE_3198 | 3.66 |  |  |
| AFE_3199 | 3.56 |  |  |
| AFE_3212 | 2.11 |  | chromosome partitioning protein parA |
| AFE_3217 | 2.12 |  | molybdenum cofactor biosynthesis protein B |
| AFE_3237 | 2.54 |  | conserved hypothetical protein |
| AFE_3238 | 2.20 |  | ferredoxin%2C putative |
| AFE_3239 | 2.25 |  | Coq7 family protein |
| AFE_3240 | 2.12 |  | Rrf2 family protein |
| AFE_3241 | 2.72 |  | OsmC/Ohr family protein |
| AFE_3244 | 2.51 |  | para-aminobenzoate synthase glutamine amidotransferase component II |
| AFE_3245 | 2.47 |  | anthranilate synthase component I |
| AFE_3253 | 2.41 |  | fructose-1%2C6-bisphosphatase%2C class II |
| AFE_3277 | 2.04 |  | argininosuccinate lyase |
| AFE_3279 | 2.20 |  | diaminopimelate decarboxylase |
| AFE_3281 | 2.17 |  | hydrogenase maturation protease |
| AFE_3282 | 3.36 |  | hydrogenase expression protein%2C putative |
| AFE_3283 | 2.09 |  | [Ni/Fe] hydrogenase%2C small subunit |
| AFE_3284 | 2.39 |  | membrane protein%2C putative |
| AFE_3285 | 2.48 |  | iron-sulfur cluster-binding protein |
| AFE_0001 | 0.44 |  | DNA polymerase III%2C beta subunit |
| AFE_0005 | 0.42 |  | spore coat protein |
| AFE_0009 | 0.05 |  |  |
| AFE_0017 | 0.07 |  | DNA processing protein DprA%2C putative |
| AFE_0031 | 0.17 |  | tRNA modification GTPase TrmE |
| AFE_0039 | 0.46 |  | RND efflux system%2C drug efflux transporter%2C outer membrane lipoprotein |
| AFE_0047 | 0.41 | 0.30±0.14 | conserved hypothetical protein |
| AFE_0049 | 0.46 | 0.21±0.01 | periplasmic solute-binding protein%2C putative |
| AFE_0050 | 0.30 | 0.26±0.03 | Tat (twin-arginine translocation) pathway signal sequence domain protein |
| AFE_0052 | 0.10 |  | hypothetical protein |
| AFE_0066 | 0.45 |  | TolA protein |
| AFE_0069 | 0.32 |  | radical SAM domain protein |
| AFE_0079 | 0.22 |  | glycosyltransferase%2C putative |
| AFE_0080 | 0.11 |  | membrane protein%2C putative |
| AFE_0082 | 0.40 |  | O antigen biosynthesis rhamnosyltransferase%2C putative |
| AFE_0083 | 0.06 |  |  |
| AFE_0091 | 0.10 |  | hypothetical protein |
| AFE_0108 | 0.20 |  | RND efflux system%2C outer membrane lipoprotein%2C NodT family |
| AFE_0109 | 0.10 |  | efflux transporter%2C RND family%2C MFP subunit |
| AFE_0111 | 0.49 |  | hypothetical protein |
| AFE_0116 | 0.07 |  | hypothetical protein |
| AFE_0117 | 0.42 |  | conserved domain protein |
| AFE_0132 | 0.19 |  | hypothetical protein |
| AFE_0144 | 0.44 |  | tetrapyrrole methylase family protein |
| AFE_0146 | 0.11 |  | hypothetical protein |
| AFE_0147 | 0.12 |  | conserved hypothetical protein |
| AFE_0167 | 0.31 |  | dimethyladenosine transferase |
| AFE_0169 | 0.41 |  | conserved domain protein |
| AFE_0171 | 0.08 |  | drug resistance transporter%2Cputative%2C EmrB/QacA subfamily |
| AFE_0182 | 0.50 |  | shikimate 5-dehydrogenase |
| AFE_0186 | 0.25 |  | type IV pilus prepilin peptidase PilD |
| AFE_0187 | 0.12 |  | dephospho-CoA kinase |
| AFE_0191 | 0.12 |  | transcriptional regulator%2C Fis family |
| AFE_0222 | 0.09 |  | hypothetical protein |
| AFE_0228 | 0.26 |  | glycosyl transferase%2C group 2 family protein |
| AFE_0229 | 0.13 |  | polysaccharide deacetylase family protein |
| AFE_0230 | 0.09 |  | glycosyl transferase%2C group 1 family protein |
| AFE_0242 | 0.48 |  | competence protein |
| AFE_0246 | 0.27 |  |  |
| AFE_0250 | 0.49 |  | membrane protein%2C putative |
| AFE_0281 | 0.49 |  |  |
| AFE_0289 | 0.42 |  | ubiquinone/menaquinone biosynthesis methlytransferase UbiE |
| AFE_0290 | 0.11 |  | hypothetical protein |
| AFE_0291 | 0.43 |  | glutathione S-transferase family protein |
| AFE_0292 | 0.31 |  | methionine biosynthesis protein MetW |
| AFE_0328 | 0.27 |  | ribosomal protein L4 |
| AFE_0353 | 0.33 |  | ribosomal protein S4 |
| AFE_0372 | 0.35 |  | tRNA pseudouridine synthase D |
| AFE_0382 | 0.46 |  | ErfK/YbiS/YcfS/YnhG family protein |
| AFE_0396 | 0.34 |  | peptidase%2C M16 family |
| AFE_0399 | 0.27 |  |  |
| AFE_0400 | 0.23 |  | DNA polymerase III%2C epsilon subunit |
| AFE_0409 | 0.35 |  | conserved domain protein |
| AFE_0412 | 0.12 |  | addiction module toxin%2C Txe/YoeB family |
| AFE_0432 | 0.49 |  | conserved hypothetical protein TIGR00726 |
| AFE_0438 | 0.33 |  |  |
| AFE_0452 | 0.28 |  | transcriptional activator%2C putative%2C Baf family |
| AFE_0456 | 0.04 |  | hypothetical protein |
| AFE_0473 | 0.47 |  | homoserine dehydrogenase |
| AFE_0475 | 0.41 |  | conserved hypothetical protein |
| AFE_0476 | 0.25 |  | single-stranded-DNA-specific exonuclease RecJ |
| AFE_0487 | 0.17 |  | conserved hypothetical protein |
| AFE_0494 | 0.37 |  |  |
| AFE_0497 | 0.33 |  |  |
| AFE_0499 | 0.06 |  | hypothetical protein |
| AFE_0500 | 0.05 |  | conserved hypothetical protein |
| AFE_0518 | 0.12 |  | conserved hypothetical protein |
| AFE_0528 | 0.44 |  | conserved hypothetical protein |
| AFE_0538 | 0.21 |  | conserved domain protein |
| AFE_0541 | 0.07 |  | hypothetical protein |
| AFE_0544 | 0.42 |  | conserved hypothetical protein |
| AFE_0555 | 0.20 |  | signal peptidase II |
| AFE_0561 | 0.15 |  | hypothetical protein |
| AFE_0576 | 0.25 |  | para-aminobenzoate synthase%2C component I |
| AFE_0577 | 0.38 |  | membrane protein%2C putative |
| AFE_0583 | 0.47 |  | hypothetical protein |
| AFE_0584 | 0.09 |  |  |
| AFE_0592 | 0.18 |  | 4-hydroxybenzoate polyprenyl transferase |
| AFE_0593 | 0.21 |  | chorismate lyase family protein |
| AFE_0621 | 0.47 |  | phosphoglycerate mutase family protein |
| AFE_0631 | 0.34 | 0.14±0.03 | cytochrome o ubiquinol oxidase%2C subunit II |
| AFE_0632 | 0.41 | 0.13±0.02 | cytochrome o ubiquinol oxidase%2C subunit I |
| AFE_0633 | 0.41 | 0.11±0.01 | cytochrome o ubiquinol oxidase%2C subunit III |
| AFE_0637 | 0.12 |  | major facilitator family transporter |
| AFE_0646 | 0.41 |  | ABC transporter%2C ATP-binding protein |
| AFE_0661 | 0.38 |  | membrane protein%2C putative |
| AFE_0681 | 0.34 |  | patatin-like phospholipase family protein |
| AFE_0686 | 0.37 |  | type I restriction-modification system%2C S subunit%2C putative |
| AFE_0687 | 0.22 |  | hypothetical protein |
| AFE_0689 | 0.23 |  | transposase%2C degenerate |
| AFE_0694 | 0.23 |  | hypothetical protein |
| AFE_0696 | 0.13 |  | S-(hydroxymethyl)glutathione synthase |
| AFE_0697 | 0.18 |  | S-(hydroxymethyl)glutathione dehydrogenase/class III alcohol dehydrogenase |
| AFE_0698 | 0.30 |  | S-formylglutathione hydrolase |
| AFE_0721 | 0.37 |  | conserved hypothetical protein |
| AFE_0739 | 0.22 |  | type IV pilus assembly protein PilM |
| AFE_0741 | 0.40 |  | cytidine and deoxycytidylate deaminase family protein |
| AFE_0750 | 0.23 |  | CDP-diacylglycerol--serine O-phosphatidyltransferse |
| AFE_0753 | 0.31 |  | chromosome segregation protein SMC |
| AFE_0763 | 0.17 |  | TonB-dependent receptor%2C putative |
| AFE_0764 | 0.49 |  | membrane protein%2C putative |
| AFE_0768 | 0.17 |  | MotA/TolQ/ExbB proton channel family protein |
| AFE_0769 | 0.03 | 0.38±0.25 | biopolymer transport protein%2C ExbD/TolR family |
| AFE_0770 | 0.04 | 0.34±0.01 | TonB family protein |
| AFE_0771 | 0.09 |  | cytosine/purines/uracil/thiamine/allantoin permease family protein |
| AFE_0772 | 0.46 |  | guanine deaminase%2C putative |
| AFE_0773 | 0.40 |  | adenosine deaminase |
| AFE_0774 | 0.47 |  | phosphorylase%2C putative |
| AFE_0775 | 0.39 |  | xanthine dehydrogenase%2C molybdenum-binding subunit%2C putative |
| AFE_0776 | 0.47 |  | xanthine dehydrogenase%2C iron-sulfur binding subunit%2C putative |
| AFE_0785 | 0.35 |  | gamma-glutamyltranspeptidase |
| AFE_0789 | 0.38 |  | dioxygenase%2C putative |
| AFE_0790 | 0.05 | 0.34±0.02 | acyltransferase%2C putative |
| AFE_0791 | 0.16 |  | Na+/H+ antiporter%2C putative |
| AFE_0798 | 0.11 |  | membrane protein%2C putative |
| AFE_0801 | 0.12 |  | conserved hypothetical protein |
| AFE_0811 | 0.11 |  | hypothetical protein |
| AFE_0812 | 0.09 |  | hypothetical protein |
| AFE_0819 | 0.06 |  | hypothetical protein |
| AFE_0823 | 0.06 |  | hypothetical protein |
| AFE_0827 | 0.39 |  | ATP-dependent DNA helicase%2C putative |
| AFE_0829 | 0.15 |  | type I restriction-modification system%2C S subunit |
| AFE_0839 | 0.05 |  | hypothetical protein |
| AFE_0840 | 0.19 |  | hypothetical protein |
| AFE_0841 | 0.18 |  | type I restriction-modification system%2C R subunit |
| AFE_0843 | 0.21 |  | conserved hypothetical protein |
| AFE_0851 | 0.42 |  | translation elongation factor P |
| AFE_0857 | 0.33 |  | transcriptional regulator%2C Crp/Fnr family |
| AFE_0862 | 0.40 |  | drug resistance transporter%2C putative |
| AFE_0873 | 0.14 |  | hypothetical protein |
| AFE_0874 | 0.07 |  |  |
| AFE_0879 | 0.30 |  | transcriptional regulator%2C LysR family |
| AFE_0885 | 0.04 |  | hypothetical protein |
| AFE_0886 | 0.20 |  |  |
| AFE_0888 | 0.23 |  | conserved hypothetical protein |
| AFE_0892 | 0.24 |  | conserved hypothetical protein |
| AFE_0901 | 0.30 |  | 3-phosphoshikimate 1-carboxyvinyltransferase |
| AFE_0905 | 0.23 |  | integration host factor%2C beta subunit |
| AFE_0906 | 0.33 |  | conserved hypothetical protein |
| AFE_0913 | 0.09 |  | hypothetical protein |
| AFE_0917 | 0.23 |  |  |
| AFE_0918 | 0.13 |  | PAP2 family protein |
| AFE_0922 | 0.24 |  | drug resistance transporter%2C EmrB/QacA family |
| AFE_0923 | 0.31 |  | conserved hypothetical protein |
| AFE_0945 | 0.37 |  | ABC transporter%2C ATP-binding protein |
| AFE_0946 | 0.04 |  | hypothetical protein |
| AFE_0964 | 0.02 |  | membrane protein%2C putative |
| AFE_0965 | 0.08 |  | hypothetical protein |
| AFE_0966 | 0.43 |  | conserved hypothetical protein |
| AFE_0983 | 0.47 |  | ABC transporter%2C ATP-binding protein%2C MsbA family |
| AFE_0993 | 0.47 |  | metallo-beta-lactamase family protein |
| AFE_1397 | 0.24 |  | GTP-binding protein LepA |
| AFE_1398 | 0.21 |  | signal peptidase I |
| AFE_1402 | 0.43 |  | DNA repair protein RecO%2C putative |
| AFE_1404 | 0.40 |  | holo-(acyl-carrier-protein) synthase |
| AFE_1405 | 0.41 |  | UDP-glucose 6-dehydrogenase |
| AFE_1408 | 0.32 |  | 33 kDa chaperonin |
| AFE_1421 | 0.06 |  | hypothetical protein |
| AFE_1422 | 0.41 |  | Ser/Thr protein phosphatase family protein |
| AFE_1423 | 0.04 |  | hypothetical protein |
| AFE_1426 | 0.14 |  | hypothetical protein |
| AFE_1427 | 0.39 |  | glycosyl transferase%2C group 1 family protein |
| AFE_1428 | 0.20 |  | cytochrome c |
| AFE_1433 | 0.39 |  | ATP-dependent RNA helicase RhlE |
| AFE_1444 | 0.48 |  | ribosomal protein S2 |
| AFE_1449 | 0.39 |  | phosphatidate cytidylyltransferase |
| AFE_1451 | 0.44 |  | membrane-associated zinc metalloprotease%2C putative |
| AFE_1453 | 0.48 |  | outer membrane protein%2C OmpH family |
| AFE_1454 | 0.32 |  | UDP-3-O-3-hydroxymyristoyl glucosamine N-acyltransferase |
| AFE_1458 | 0.41 |  | pleiotropic regulatory protein%2C putative |
| AFE_1459 | 0.13 |  | lipid A disaccharide synthase LpxB |
| AFE_1482 | 0.11 |  | conserved domain protein |
| AFE_1483 | 0.21 |  | TonB-dependent receptor |
| AFE_1484 | 0.24 |  | conserved hypothetical protein |
| AFE_1487 | 0.23 |  | TonB family protein |
| AFE_1489 | 0.18 |  | iron compound ABC transporter%2C periplasmic iron-binding protein%2C putative |
| AFE_1490 | 0.23 |  | ABC transporter%2C permease protein%2C FecCD family |
| AFE_1491 | 0.16 |  | iron compound ABC transporter%2C ATP-binding protein%2C putative |
| AFE_1492 | 0.15 |  | TonB-dependent receptor |
| AFE_1494 | 0.24 |  | iron compound ABC transporter%2C periplasmic iron-binding protein%2C putative |
| AFE_1497 | 0.25 | 0.30±0.10 | conserved hypothetical protein |
| AFE_1498 | 0.35 |  | ABC transporter%2C periplasmic substrate-binding protein%2C putative |
| AFE_1499 | 0.22 |  | ATPase%2C AAA family |
| AFE_1510 | 0.17 |  | conserved hypothetical protein |
| AFE_1511 | 0.35 |  | nitrogen fixation protein NifQ |
| AFE_1514 | 0.14 |  | conserved hypothetical protein |
| AFE_1516 | 0.48 |  | nitrogenase molybdenum-iron cofactor biosynthesis protein NifN |
| AFE_1517 | 0.26 |  | nitrogenase MoFe cofactor biosynthesis protein NifE |
| AFE_1524 | 0.33 |  | NAD(+)--dinitrogen-reductase ADP-D-ribosyltransferase |
| AFE_1526 | 0.42 |  | hypothetical protein |
| AFE_1543 | 0.48 |  | conserved hypothetical protein |
| AFE_1554 | 0.48 |  | membrane protein%2C putative |
| AFE_1555 | 0.06 |  | tRNA-Val |
| AFE_1584 | 0.33 |  | DNA-binding protein HU family protein |
| AFE_1612 | 0.09 |  | hypothetical protein |
| AFE_1615 | 0.22 |  | DNA primase TraC |
| AFE_1616 | 0.39 |  | hypothetical protein |
| AFE_1620 | 0.14 |  | hypothetical protein |
| AFE_1621 | 0.20 |  | conserved hypothetical protein |
| AFE_1624 | 0.38 |  | hypothetical protein |
| AFE_1631 | 0.40 |  | conserved hypothetical protein |
| AFE_1632 | 0.08 |  | hypothetical protein |
| AFE_1633 | 0.06 |  | hypothetical protein |
| AFE_1635 | 0.07 | 0.05±0.01 | membrane protein%2C putative |
| AFE_1642 | 0.45 |  | conjugal transfer protein trbL%2C putative |
| AFE_1644 | 0.15 |  | conserved hypothetical protein |
| AFE_1646 | 0.37 |  | serine protease%2C DO/DeqQ family |
| AFE_1647 | 0.15 |  | hypothetical protein |
| AFE_1650 | 0.08 |  | hypothetical protein |
| AFE_1654 | 0.45 |  |  |
| AFE_1656 | 0.31 |  | ferredoxin/oxidoreductase%2C FAD/NAD-binding |
| AFE_1657 | 0.50 |  | hybrid cluster protein |
| AFE_1663 | 0.24 |  | glycolate oxidase%2C iron-sulfur subunit |
| AFE_1664 | 0.19 |  | glycolate oxidase%2C subunit GlcE |
| AFE_1671 | 0.14 |  | hypothetical protein |
| AFE_1672 | 0.40 |  | dihydroorotase%2C homodimeric type |
| AFE_1693 | 0.34 |  | conserved hypothetical protein |
| AFE_1695 | 0.14 |  | conjugal transfer protein |
| AFE_1696 | 0.37 | 0.36±0.10 | conjugal transfer protein%2C putative |
| AFE_1697 | 0.38 |  |  |
| AFE_1699 | 0.22 |  | TraG/TraD family protein |
| AFE_1704 | 0.14 |  | hypothetical protein |
| AFE_1710 | 0.26 |  | hypothetical protein |
| AFE_1713 | 0.41 |  | hypothetical protein |
| AFE_1714 | 0.11 |  | hypothetical protein |
| AFE_1715 | 0.03 |  | hypothetical protein |
| AFE_1718 | 0.21 |  | hypothetical protein |
| AFE_1720 | 0.43 |  | parA family protein |
| AFE_1722 | 0.12 |  | hypothetical protein |
| AFE_1724 | 0.39 |  | hypothetical protein |
| AFE_1726 | 0.17 | 0.35±0.08 | conjugal transfer protein TraF%2C putative |
| AFE_1729 | 0.08 |  | hypothetical protein |
| AFE_1730 | 0.10 |  | hypothetical protein |
| AFE_1731 | 0.05 | 0.31±0.08 | conjugal transfer protein TraB%2C putative |
| AFE_1732 | 0.16 |  | hypothetical protein |
| AFE_1738 | 0.34 | 0.33±0.32 | DNA topoisomerase III |
| AFE_1765 | 0.08 |  | hypothetical protein |
| AFE_1767 | 0.27 |  | exodeoxyribonuclease X%2C putative |
| AFE_1768 | 0.04 |  | hypothetical protein |
| AFE_1778 | 0.50 | 0.56±0.01 | GMP synthase |
| AFE_1783 | 0.34 |  |  |
| AFE_1784 | 0.41 |  | mechanosensitive ion channel family protein |
| AFE_1786 | 0.35 |  | conserved hypothetical protein |
| AFE_1787 | 0.40 |  | Sua5/YciO/YrdC/YwlC family protein |
| AFE_1791 | 0.14 |  | segregation and condensation protein B |
| AFE_1794 | 0.22 |  | base excision repair protein%2C HhH-GPD family |
| AFE_1798 | 0.50 |  | membrane protein%2C putative |
| AFE_1822 | 0.19 |  | hypothetical protein |
| AFE_1824 | 0.22 |  | membrane protein%2C putative |
| AFE_1829 | 0.11 |  | tRNA-Asn |
| AFE_1830 | 0.18 |  | toluene tolerance protein%2C putative |
| AFE_1835 | 0.15 |  | hypothetical protein |
| AFE_1837 | 0.11 |  | hypothetical protein |
| AFE_1839 | 0.41 |  | conserved hypothetical protein |
| AFE_1891 | 0.45 |  | ribonuclease R |
| AFE_1899 | 0.21 |  | hypothetical protein |
| AFE_1906 | 0.26 |  | 3-oxoacyl-(acyl-carrier-protein) synthase III |
| AFE_1915 | 0.27 |  | type IV pilus assembly protein PilZ%2C putative |
| AFE_1920 | 0.37 |  | conserved hypothetical protein TIGR00103 |
| AFE_1921 | 0.31 |  | recombination protein RecR |
| AFE_1922 | 0.22 |  | ammonium transporter family protein |
| AFE_1937 | 0.23 |  | ribosome-associated GTPase EngA |
| AFE_1938 | 0.05 |  | hypothetical protein |
| AFE_1940 | 0.29 |  | phosphate ABC transporter%2C permease protein PstC |
| AFE_1941 | 0.29 |  | phosphate ABC transporter%2C permease protein%2C putative |
| AFE_1943 | 0.22 |  | dsbG domain protein |
| AFE_1945 | 0.21 |  | membrane protein%2C putative |
| AFE_1948 | 0.17 |  | heavy metal efflux transporter%2C MFP subunit%2C putative |
| AFE_1950 | 0.05 |  | hypothetical protein |
| AFE_1953 | 0.16 |  | ferredoxin%2C putative |
| AFE_1959 | 0.34 |  | hypothetical protein |
| AFE_1961 | 0.26 |  | AmpG permease protein%2C putative |
| AFE_1971 | 0.38 |  | transporter%2C putative |
| AFE_1979 | 0.31 |  | thioredoxin |
| AFE_1988 | 0.45 |  | cation-transporting P-type ATPase%2C putative |
| AFE_1989 | 0.10 |  |  |
| AFE_1990 | 0.20 |  | transcriptional regulator%2C AsnC family |
| AFE_1999 | 0.02 |  | autoinducer synthesis protein |
| AFE_2002 | 0.16 |  | conserved hypothetical protein |
| AFE_2003 | 0.08 |  |  |
| AFE_2013 | 0.12 |  | transcriptional regulator%2C MarR family |
| AFE_2016 | 0.07 |  | tRNA-Arg |
| AFE_2022 | 0.39 |  | aspartate aminotransferase |
| AFE_2038 | 0.03 |  | hypothetical protein |
| AFE_2043 | 0.48 |  | transporter%2C AcrB/AcrD/AcrF family |
| AFE_2044 | 0.38 |  | efflux transporter%2C RND family%2C MFP subunit |
| AFE_2049 | 0.02 |  | hypothetical protein |
| AFE_2065 | 0.45 |  | CvpA family protein |
| AFE_2085 | 0.02 |  | conserved hypothetical protein |
| AFE_2086 | 0.16 |  | heat shock protein%2C Hsp20 family |
| AFE_2090 | 0.32 |  | peptidase%2C M48 family |
| AFE_2091 | 0.41 |  | drug resistance transporter%2C EmrB/QacA family |
| AFE_2092 | 0.03 |  | hypothetical protein |
| AFE_2096 | 0.16 |  | hypothetical protein |
| AFE_2097 | 0.07 |  | hypothetical protein |
| AFE_2100 | 0.09 |  | hypothetical protein |
| AFE_2102 | 0.22 |  |  |
| AFE_2104 | 0.42 |  | transporter%2C AcrB/AcrD/AcrF family |
| AFE_2105 | 0.11 |  | ABC transporter%2C ATP-binding protein |
| AFE_2106 | 0.09 |  | ABC transporter%2C permease protein%2C putative |
| AFE_2107 | 0.04 | 0.13±0.05 | ABC transporter%2C permease protein%2C putative |
| AFE_2117 | 0.28 |  | membrane protein%2C putative |
| AFE_2121 | 0.20 |  | glycosyl transferase%2C group 2 family protein |
| AFE_2123 | 0.45 |  | MTA/SAH nucleosidase%2C putative |
| AFE_2128 | 0.40 |  | tRNA-Glu |
| AFE_2133 | 0.38 |  | universal stress family protein |
| AFE_2154 | 0.30 |  | hydrogenase-4%2C I subunit |
| AFE_2157 | 0.30 |  | von Willebrand factor type A domain protein |
| AFE_2159 | 0.12 |  | alkylphosphonate utilization operon protein PhnA |
| AFE_2163 | 0.06 |  | hypothetical protein |
| AFE_2164 | 0.11 |  | hypothetical protein |
| AFE_2165 | 0.06 |  | hypothetical protein |
| AFE_2166 | 0.30 |  | toxin secretion protein%2C HlyD family |
| AFE_2167 | 0.13 |  | toxin secretion ABC transporter%2C ATP-binding protein%2C HlyB family |
| AFE_2168 | 0.07 |  | hypothetical protein |
| AFE_2169 | 0.02 | 0.35±0.16 | outer membrane toxin secretion efflux protein%2C putative |
| AFE_2177 | 0.46 |  | sulfur reductase%2C molybdopterin subunit |
| AFE_2183 | 0.18 |  | universal stress family protein |
| AFE_2184 | 0.36 |  | drug resistance transporter%2C EmrB/QacA family |
| AFE_2187 | 0.08 |  | ATP-dependent DNA helicase Rep |
| AFE_2197 | 0.06 |  | hypothetical protein |
| AFE_2205 | 0.32 |  | Na+/H+ antiporter NhaA |
| AFE_2217 | 0.36 |  | hydrolase%2C alpha/beta hydrolase fold family |
| AFE_2227 | 0.22 |  | HAD-superfamily hydrolase%2C subfamily IA |
| AFE_2228 | 0.44 |  | DNA-binding response regulator KdpE |
| AFE_2237 | 0.04 |  | hypothetical protein |
| AFE_2239 | 0.08 |  | hypothetical protein |
| AFE_2244 | 0.04 |  | hypothetical protein |
| AFE_2246 | 0.40 |  | dsbG domain protein |
| AFE_2247 | 0.10 |  | hypothetical protein |
| AFE_2248 | 0.32 |  | lipoprotein%2C putative |
| AFE_2249 | 0.11 |  | hypothetical protein |
| AFE_2264 | 0.43 |  | dihydroorotate dehydrogenase |
| AFE_2267 | 0.22 |  | phosphoesterase family protein |
| AFE_2274 | 0.35 |  | conserved hypothetical protein |
| AFE_2276 | 0.06 |  | conserved hypothetical protein |
| AFE_2277 | 0.12 |  | major facilitator family transporter |
| AFE_2281 | 0.48 |  | phosphonate metabolism protein PhnJ |
| AFE_2282 | 0.17 |  | phosphonate ABC transporter%2C ATP-binding protein PhnK |
| AFE_2283 | 0.18 |  | phosphonate ABC transporter%2C ATP-binding protein PhnL |
| AFE_2284 | 0.46 |  | phnM protein%2C putative |
| AFE_2285 | 0.30 |  | conserved hypothetical protein |
| AFE_2286 | 0.32 |  | PhnN protein%2C putative |
| AFE_2288 | 0.08 |  | TonB-dependent receptor |
| AFE_2289 | 0.14 |  |  |
| AFE_2290 | 0.08 |  | hypothetical protein |
| AFE_2292 | 0.06 | 0.25±0.10 | TonB-dependent receptor |
| AFE_2294 | 0.06 |  |  |
| AFE_2295 | 0.06 |  | conserved hypothetical protein |
| AFE_2296 | 0.05 |  | conserved domain protein |
| AFE_2298 | 0.29 |  | TonB-dependent receptor |
| AFE_2299 | 0.04 |  | MotA/TolQ/ExbB proton channel family protein |
| AFE_2301 | 0.15 |  | TonB family protein |
| AFE_2302 | 0.46 |  | TonB-dependent receptor |
| AFE_2303 | 0.12 |  | conserved hypothetical protein |
| AFE_2304 | 0.14 |  | TonB family protein |
| AFE_2306 | 0.10 |  | DNA-binding response regulator |
| AFE_2308 | 0.07 |  | hypothetical protein |
| AFE_2309 | 0.45 |  | hypothetical protein |
| AFE_2311 | 0.06 |  | hypothetical protein |
| AFE_2312 | 0.20 |  | sugar transporter family protein |
| AFE_2329 | 0.30 |  | hypothetical protein |
| AFE_2330 | 0.36 |  | membrane protein%2C putative |
| AFE_2331 | 0.05 |  | hypothetical protein |
| AFE_2332 | 0.08 |  | transcriptional regulator%2C LysR family |
| AFE_2333 | 0.11 |  | major facilitator family transporter |
| AFE_2338 | 0.44 |  | MutS2 family protein |
| AFE_2339 | 0.44 |  | GatB/Yqey domain protein |
| AFE_2345 | 0.47 |  | ATP-dependent protease La domain protein |
| AFE_2353 | 0.08 |  | tRNA-Ala |
| AFE_2354 | 0.23 |  | phosphoribosylformylglycinamidine synthase |
| AFE_2355 | 0.40 |  | phosphoribosylaminoimidazole-succinocarboxamide synthase |
| AFE_2356 | 0.44 |  | adenylosuccinate lyase |
| AFE_2370 | 0.23 |  | tRNA-Gly |
| AFE_2379 | 0.05 |  | hypothetical protein |
| AFE_2382 | 0.03 |  | hypothetical protein |
| AFE_2385 | 0.32 |  | SCP-2 sterol transfer family protein |
| AFE_2392 | 0.23 |  | hypothetical protein |
| AFE_2394 | 0.49 |  | ISAfe5%2C transposase orfB |
| AFE_2395 | 0.27 |  |  |
| AFE_2396 | 0.05 |  | hypothetical protein |
| AFE_2404 | 0.03 |  | transposase%2C degenerate |
| AFE_2412 | 0.24 |  | phosphoenolpyruvate-utilizing enzyme mobile domain protein |
| AFE_2413 | 0.20 |  | transposase%2C degenerate |
| AFE_2419 | 0.27 |  | plasmid recombination enzyme%2C putative |
| AFE_2421 | 0.48 |  | Cd(II)/Pb(II)-responsive transcriptional regulator |
| AFE_2422 | 0.05 | 0.39±0.02 | conserved domain protein |
| AFE_2424 | 0.23 |  | cation efflux permease%2C putative |
| AFE_2430 | 0.27 |  | conserved hypothetical protein |
| AFE_2431 | 0.38 |  | heavy metal efflux pump%2C CzcA family |
| AFE_2471 | 0.20 |  | DNA-binding protein HU family protein |
| AFE_2477 | 0.38 |  |  |
| AFE_2478 | 0.39 |  | hypothetical protein |
| AFE_2484 | 0.15 |  | hypothetical protein |
| AFE_2488 | 0.26 |  | hypothetical protein |
| AFE_2490 | 0.26 |  | hypothetical protein |
| AFE_2492 | 0.36 |  | hypothetical protein |
| AFE_2494 | 0.23 |  | conserved hypothetical protein |
| AFE_2495 | 0.28 |  | hypothetical protein |
| AFE_2502 | 0.28 |  | conserved hypothetical protein |
| AFE_2512 | 0.26 |  | hypothetical protein |
| AFE_2521 | 0.26 |  | hypothetical protein |
| AFE_2525 | 0.32 |  | hypothetical protein |
| AFE_2535 | 0.45 |  | von Willebrand factor type A domain protein |
| AFE_2538 | 0.16 |  | hypothetical protein |
| AFE_2539 | 0.48 |  | hypothetical protein |
| AFE_2544 | 0.44 |  | geranylgeranyl reductase family protein |
| AFE_2545 | 0.35 |  | lipoate-protein ligase A%2C putative |
| AFE_2565 | 0.47 |  | penicillin-binding protein 2 |
| AFE_2566 | 0.24 |  | rod shape-determining protein RodA |
| AFE_2584 | 0.19 | 0.87±0.25 | acyltransferase family protein |
| AFE_2593 | 0.17 |  | hypothetical protein |
| AFE_2594 | 0.48 |  | glutathione S-transferase |
| AFE_2603 | 0.44 |  | conserved hypothetical protein |
| AFE_2605 | 0.23 |  | ABC transporter%2C permease protein%2C putative |
| AFE_2646 | 0.16 |  | hypothetical protein |
| AFE_2647 | 0.17 |  | hypothetical protein |
| AFE_2648 | 0.35 |  | hypothetical protein |
| AFE_2652 | 0.21 |  | hypothetical protein |
| AFE_2653 | 0.05 |  | hypothetical protein |
| AFE_2668 | 0.06 |  | hypothetical protein |
| AFE_2670 | 0.40 |  | ribosomal protein L28 |
| AFE_2689 | 0.22 |  | TonB family protein |
| AFE_2690 | 0.23 |  | type I secretion outer membrane protein%2C putative |
| AFE_2692 | 0.35 |  | type I secretion membrane fusion protein%2C HlyD family |
| AFE_2694 | 0.42 |  | conserved hypothetical protein |
| AFE_2695 | 0.25 |  | hypothetical protein |
| AFE_2698 | 0.27 |  | hypothetical protein |
| AFE_2707 | 0.45 |  | conserved hypothetical protein |
| AFE_2711 | 0.10 |  | hypothetical protein |
| AFE_2713 | 0.31 |  | hypothetical protein |
| AFE_2721 | 0.21 |  | lipoate-protein ligase A%2C putative |
| AFE_2722 | 0.11 |  | peptidase%2C U32 family |
| AFE_2723 | 0.23 |  | peptidase%2C U32 family |
| AFE_2724 | 0.16 |  | conserved hypothetical protein |
| AFE_2726 | 0.36 |  | hypothetical protein |
| AFE_2734 | 0.19 |  | hypothetical protein |
| AFE_2756 | 0.25 |  | conserved hypothetical protein |
| AFE_2758 | 0.17 |  | formamidopyrimidine-DNA glycosylase |
| AFE_2759 | 0.12 |  | modification methylase%2C HemK family |
| AFE_2760 | 0.23 |  | peptide chain release factor 1 |
| AFE_2766 | 0.40 |  | ribose-phosphate pyrophosphokinase |
| AFE_2774 | 0.18 |  | transposase%2C degenerate |
| AFE_2775 | 0.11 |  | membrane protein |
| AFE_2776 | 0.43 |  | benzoylformate decarboxylase |
| AFE_2783 | 0.15 |  | hypothetical protein |
| AFE_2785 | 0.44 |  | hypothetical protein |
| AFE_2787 | 0.04 |  | hypothetical protein |
| AFE_2788 | 0.28 |  | conserved hypothetical protein |
| AFE_2789 | 0.06 |  | hypothetical protein |
| AFE_2792 | 0.45 |  | conserved hypothetical protein |
| AFE_2794 | 0.35 |  | hypothetical protein |
| AFE_2795 | 0.13 |  | hypothetical protein |
| AFE_2797 | 0.11 |  | hypothetical protein |
| AFE_2798 | 0.18 |  |  |
| AFE_2813 | 0.12 |  | hypothetical protein |
| AFE_2825 | 0.38 |  | beta-hexosaminidase |
| AFE_2827 | 0.37 |  | ErfK/YbiS/YcfS/YnhG family protein |
| AFE_2849 | 0.46 |  | serine protease%2C DO/DeqQ family |
| AFE_2850 | 0.33 |  | tyrosine recombinase XerD |
| AFE_2857 | 0.25 |  | arsenical resistance protein ArsH |
| AFE_2875 | 0.09 |  | transcriptional regulator%2C LysR family |
| AFE_2876 | 0.18 |  | conserved hypothetical protein |
| AFE_2877 | 0.43 |  | conserved hypothetical protein |
| AFE_2878 | 0.03 |  | hypothetical protein |
| AFE_2879 | 0.12 |  | conserved hypothetical protein |
| AFE_2887 | 0.15 |  | hypothetical protein |
| AFE_2909 | 0.30 |  | hypothetical protein |
| AFE_2925 | 0.25 |  | dolichyl-phosphate-mannose-protein mannosyltransferase family protein |
| AFE_2934 | 0.43 |  | transcriptional regulator%2C putative |
| AFE_2935 | 0.36 |  | TonB-dependent receptor |
| AFE_2959 | 0.38 |  | conserved hypothetical protein |
| AFE_2962 | 0.02 | 0.40±0.16 | capsule polysaccharide exporter%2C inner-membrane protein CtrC |
| AFE_2963 | 0.06 |  | capsule polysaccharide exporter%2C ATP-binding protein |
| AFE_2964 | 0.17 |  | methyltransferase%2C FkbM family domain protein |
| AFE_2968 | 0.18 |  | hypothetical protein |
| AFE_2969 | 0.08 |  | hypothetical protein |
| AFE_2970 | 0.47 |  |  |
| AFE_2973 | 0.22 |  | 3'(2')%2C5'-bisphosphate nucleotidase |
| AFE_2985 | 0.14 |  | ABC transporter%2C permease/ATP-binding protein |
| AFE_2986 | 0.10 |  | acetyltransferase%2C GNAT family |
| AFE_2987 | 0.14 |  | peptide ABC transporter%2C periplasmic peptide-binding protein |
| AFE_2988 | 0.36 |  | peptide ABC transporter%2C ATP-binding protein |
| AFE_2989 | 0.18 |  | peptide ABC transporter%2C ATP-binding protein |
| AFE_2990 | 0.46 |  | peptide ABC transporter%2C permease protein |
| AFE_2991 | 0.26 |  | peptide ABC transporter%2C permease protein |
| AFE_2992 | 0.16 |  | peptide ABC transporter%2C periplasmic peptide-binding protein |
| AFE_2993 | 0.32 |  | conserved hypothetical protein |
| AFE_2994 | 0.24 |  | conserved hypothetical protein |
| AFE_2997 | 0.03 |  | hypothetical protein |
| AFE_2998 | 0.11 |  | TonB-dependent receptor |
| AFE_3000 | 0.11 |  | lactate/malate dehydrogenase family protein |
| AFE_3003 | 0.12 |  | MotA/TolQ/ExbB proton channel family protein |
| AFE_3005 | 0.06 | 0.24±0.09 | acetyltransferase%2C GNAT family |
| AFE_3015 | 0.16 |  | A/G-specific adenine glycosylase |
| AFE_3016 | 0.47 |  | capsular polysaccharide biosynthesis protein%2C putative |
| AFE_3017 | 0.09 |  | hypothetical protein |
| AFE_3036 | 0.12 |  | ABC-2 type transporter |
| AFE_3050 | 0.14 |  | Sec-independent protein translocase TatC |
| AFE_3055 | 0.23 |  | hypothetical protein |
| AFE_3069 | 0.44 |  | pyruvate dehydrogenase%2C E1 component%2C pyruvate dehydrogenase beta subunit |
| AFE_3073 | 0.30 |  | N-acetyl-gamma-glutamyl-phosphate reductase |
| AFE_3076 | 0.03 |  | hypothetical protein |
| AFE_3077 | 0.25 |  | hypothetical protein |
| AFE_3100 | 0.49 |  | competence protein ComEA helix-hairpin-helix repeat protein |
| AFE_3105 | 0.32 |  | ISAfe6%2C transposase%2C degenerate |
| AFE_3106 | 0.32 |  | hypothetical protein |
| AFE_3112 | 0.30 |  | cytochrome c-type biogenesis protein ResB |
| AFE_3114 | 0.28 |  | conserved hypothetical protein |
| AFE_3120 | 0.25 |  | membrane protein%2C putative |
| AFE_3123 | 0.34 |  | adenylylsulfate reductase%2C thioredoxin dependent |
| AFE_3129 | 0.12 |  | voltage-gated chloride channel |
| AFE_3132 | 0.41 |  | hypothetical protein |
| AFE_3137 | 0.40 |  | DNA-binding response regulator |
| AFE_3138 | 0.37 |  | oxidoreductase%2C 2OG-Fe(II) oxygenase family |
| AFE_3139 | 0.23 |  | hypothetical protein |
| AFE_3155 | 0.26 |  | hypothetical protein |
| AFE_3156 | 0.11 |  | hypothetical protein |
| AFE_3157 | 0.15 |  | ISAfe6%2C transposase%2C degenerate |
| AFE_3162 | 0.37 |  | acetyltransferase%2C GNAT family |
| AFE_3184 | 0.41 |  |  |
| AFE_3185 | 0.13 |  |  |
| AFE_3221 | 0.21 |  | magnesium and cobalt transport protein%2C putative |
| AFE_3223 | 0.45 |  | WrbA family protein |
| AFE_3224 | 0.35 |  | pirin family protein |
| AFE_3246 | 0.42 |  |  |
| AFE_3261 | 0.50 |  | lipoprotein%2C putative |
| AFE_3266 | 0.17 |  | conserved domain protein |
| AFE_3267 | 0.37 |  | YaiI/YqxD family protein |
| AFE_3271 | 0.32 |  | hypothetical protein |
| AFE_3272 | 0.07 |  | hypothetical protein |
| AFE_3297 | 0.11 |  | TonB family protein |
| Ferrous ---*afeI overexpression* vs WT(pJRD215) | | | |
| AFE_0001 | 2.67 |  | DNA polymerase III%2C beta subunit |
| AFE_0007 | 2.38 |  | outer membrane usher protein%2C putative |
| AFE_0008 | 3.81 | 2.53±0.12 | spore coat protein |
| AFE_0020 | 2.05 |  | methionyl-tRNA formyltransferase |
| AFE_0022 | 2.79 |  | hypothetical protein |
| AFE_0029 | #### | 9.85±0.79 | tetrathionate hydrolase |
| AFE_0030 | #### |  | hypothetical protein |
| AFE_0032 | 2.28 |  | inner membrane protein%2C 60 kDa |
| AFE_0040 | 2.77 |  | drug resistance transporter%2C EmrB/QacA family |
| AFE_0058 | 3.14 |  | acyltransferase family protein |
| AFE_0064 | 2.19 |  | TolQ protein |
| AFE_0065 | 2.48 |  | TolR protein |
| AFE_0068 | 2.44 |  | exsB protein |
| AFE_0072 | 2.01 |  | conserved hypothetical protein |
| AFE_0076 | 2.94 |  | polysaccharide biosynthesis protein |
| AFE_0077 | 2.67 |  | conserved hypothetical protein |
| AFE_0078 | 3.03 |  | glycosyl transferase%2C group 2 family protein |
| AFE_0098 | 2.86 |  | DoxD family protein/pyridine nucleotide-disulfide oxidoreductase |
| AFE_0138 | 3.06 |  | LPS heptosyltransferase II |
| AFE_0139 | 2.29 |  | conserved hypothetical protein |
| AFE_0142 | 2.28 |  | conserved hypothetical protein TIGR00252 |
| AFE_0194 | 2.79 |  | ribosomal protein L11 methyltransferase |
| AFE_0195 | 2.10 |  | acetyl-CoA carboxylase%2C biotin carboxylase |
| AFE_0196 | 2.03 |  | acetyl-CoA carboxylase%2C biotin carboxyl carrier protein |
| AFE_0227 | 2.64 |  | mutator mutT protein/thiamine-phosphate pyrophosphorylase family protein |
| AFE_0237 | 2.48 |  | 3-deoxy-D-manno-octulosonic-acid transferase |
| AFE_0298 | 2.52 |  | riboflavin synthase%2C alpha subunit |
| AFE_0300 | 2.88 |  | 6%2C7-dimethyl-8-ribityllumazine synthase |
| AFE_0338 | 2.36 |  | ribosomal protein L24 |
| AFE_0344 | 2.50 |  | ribosomal protein S5 |
| AFE_0355 | 2.04 |  | ribosomal protein L17 |
| AFE_0365 | 2.21 |  | methylthioadenosine phosphorylase |
| AFE_0369 | 2.16 |  | transcription-repair coupling factor |
| AFE_0446 | 2.18 |  | conserved hypothetical protein |
| AFE_0464 | 2.03 |  | hypothetical protein |
| AFE_0482 | 2.49 |  | conserved hypothetical protein |
| AFE_0485 | 2.02 |  | MotA/TolQ/ExbB proton channel family protein |
| AFE_0496 | 6.15 |  | bacterial transferase hexapeptide repeat protein |
| AFE_0556 | 2.44 |  | isoleucyl-tRNA synthetase |
| AFE_0557 | 2.33 |  | riboflavin biosynthesis protein RibF |
| AFE_0597 | 2.60 |  | PhoH-like protein |
| AFE_0598 | 2.72 |  | conserved hypothetical protein TIGR00043 |
| AFE_0599 | 2.69 |  | magnesium and cobalt efflux protein%2C putative |
| AFE_0601 | 2.30 |  | apolipoprotein N-acyltransferase |
| AFE_0646 | 2.20 |  | ABC transporter%2C ATP-binding protein |
| AFE_0649 | 3.29 |  | endonuclease/exonuclease/phosphatase family protein |
| AFE_0651 | 2.70 |  | hypothetical protein |
| AFE_0654 | 2.45 |  | ABC transporter%2C permease protein |
| AFE_0660 | 2.24 |  | malic enzyme family protein |
| AFE_0687 | 3.70 |  | hypothetical protein |
| AFE_0746 | 2.00 |  | acetolactate synthase%2C small subunit |
| AFE_0748 | 3.57 | 2.02±0.08 | phosphatidylserine decarboxylase |
| AFE_0750 | 2.56 |  | CDP-diacylglycerol--serine O-phosphatidyltransferse |
| AFE_0800 | 3.53 |  | conserved hypothetical protein |
| AFE_0802 | 2.93 |  | AMP-binding protein |
| AFE_0827 | 2.06 |  | ATP-dependent DNA helicase%2C putative |
| AFE_0828 | 3.23 |  | conserved hypothetical protein |
| AFE_0840 | 3.54 |  | hypothetical protein |
| AFE_0917 | 2.19 |  |  |
| AFE_0929 | 4.25 |  | phosphatidylglycerophosphatase A |
| AFE_0951 | 2.05 |  | aldehyde dehydrogenase (NAD) family protein |
| AFE_0954 | 2.08 | 1.61±0.21 | cytochrome d ubiquinol oxidase%2C subunit II |
| AFE_0955 | 2.12 | 4.04±1.32 | cytochrome d ubiquinol oxidase%2C subunit I |
| AFE_0966 | 2.58 |  | conserved hypothetical protein |
| AFE_0993 | 2.16 |  | metallo-beta-lactamase family protein |
| AFE_1387 | 2.11 |  | conserved hypothetical protein |
| AFE_1408 | 2.26 |  | 33 kDa chaperonin |
| AFE_1435 | 2.65 |  | phosphate regulon sensor protein PhoR |
| AFE_1443 | 3.87 |  | membrane protein%2C DedA family |
| AFE_1451 | 2.22 |  | membrane-associated zinc metalloprotease%2C putative |
| AFE_1468 | 2.01 |  | antioxidant%2C AhpC/Tsa family |
| AFE_1544 | 2.07 |  | pyrophosphorylase%2C putative |
| AFE_1554 | 2.08 |  | membrane protein%2C putative |
| AFE_1725 | 3.18 |  | sensory box-containing diguanylate cyclase%2C putative |
| AFE_1769 | 4.12 |  | oxidoreductase%2C FAD-binding%2C putative |
| AFE_1770 | 2.09 |  | pantoate--beta-alanine ligase |
| AFE_1781 | 2.40 |  | transcriptional regulator%2C AbrB family |
| AFE_1788 | 2.20 |  | membrane-associated zinc metalloprotease%2C putative |
| AFE_1797 | 3.40 |  | drug resistance transporter%2C EmrB/QacA family |
| AFE_1808 | 2.31 |  | phosphate acetyl/butyryl transferase |
| AFE_1817 | 2.59 |  | sulfotransferase domain protein |
| AFE_1819 | 2.08 |  | succinyl-diaminopimelate desuccinylase |
| AFE_1821 | 2.16 |  | aminotransferase%2C classes I and II |
| AFE_1831 | 2.37 |  | hypothetical protein |
| AFE_1832 | 3.87 |  | hypothetical protein |
| AFE_1833 | 3.17 |  | RNA polymerase sigm-70 factor family |
| AFE_1860 | 2.42 |  | hypothetical protein |
| AFE_1876 | 2.90 |  | polyphosphate kinase |
| AFE_1891 | 2.19 |  | ribonuclease R |
| AFE_1900 | 2.27 |  | CDP-diacylglycerol--glycerol-3-phosphate 3-phosphatidyltransferase%2C putative |
| AFE_1901 | 3.01 |  | conserved hypothetical protein |
| AFE_1905 | 2.43 |  | fatty acid/phospholipid synthesis protein PlsX |
| AFE_1926 | 2.10 |  | conserved hypothetical protein TIGR00150 |
| AFE_1927 | 3.08 |  | mismatch repair protein MutL |
| AFE_1928 | 3.05 |  | tRNA delta(2)-isopentenylpyrophosphate transferase |
| AFE_1935 | 2.06 |  | conserved hypothetical protein |
| AFE_1936 | 2.50 |  | PQQ enzyme repeat domain protein |
| AFE_1957 | 3.25 |  | conserved hypothetical protein |
| AFE_1958 | 3.25 |  | outer membrane protein%2C OMP85 family |
| AFE_1979 | 2.75 |  | thioredoxin |
| AFE_1987 | 2.71 |  | UDP-2%2C3-diacylglucosamine hydrolase |
| AFE_1998 | 2.62 | 2.24±0.21 | hypothetical protein |
| AFE_1999 | #### | 27.18±1.43 | autoinducer synthesis protein |
| AFE_2026 | 4.31 | 2.27±0.19 | transcriptional regulator%2C ArsR family |
| AFE_2027 | 2.04 |  | tRNA-dihydrouridine synthase A |
| AFE_2031 | 3.58 | 5.16±0.08 | sensor histidine kinase |
| AFE_2032 | 3.91 | 2.30±0.09 | DNA-binding response regulator |
| AFE_2033 | 3.58 |  | conserved domain protein |
| AFE_2034 | 2.03 |  | conserved domain protein |
| AFE_2040 | 3.89 | 2.75±0.08 | TonB-dependent receptor |
| AFE_2059 | 5.75 | 9.51±0.14 | queuine tRNA-ribosyltransferase |
| AFE_2060 | 2.94 |  | S-adenosylmethionine:tRNA ribosyltransferase-isomerase |
| AFE_2061 | 3.09 |  | CBS domain protein |
| AFE_2081 | 3.11 |  | alpha-amylase family protein |
| AFE_2122 | 2.56 |  | conserved hypothetical protein |
| AFE_2124 | 2.19 |  | squalene-hopene cyclase |
| AFE_2126 | 2.34 |  | glycerol-3-phosphate dehydrogenase (NAD(P)+) |
| AFE_2132 | 3.27 |  | prolyl-tRNA synthetase |
| AFE_2133 | 3.26 |  | universal stress family protein |
| AFE_2138 | 3.66 |  | PDZ domain protein |
| AFE_2161 | 8.97 |  | conserved hypothetical protein |
| AFE_2224 | 2.32 |  | glycosyl hydrolase%2C family 15 |
| AFE_2225 | 2.49 |  | glucose 1-dehydrogenase%2C putative |
| AFE_2227 | 2.59 |  | HAD-superfamily hydrolase%2C subfamily IA |
| AFE_2238 | 3.72 | 4.87±0.07 | amino acid kinase family protein |
| AFE_2257 | 3.77 |  | conserved hypothetical protein |
| AFE_2267 | 2.21 |  | phosphoesterase family protein |
| AFE_2269 | 3.16 |  | biopolymer transport protein%2C ExbD/TolR family |
| AFE_2270 | 4.59 | 2.53±0.07 | MotA/TolQ/ExbB proton channel family protein |
| AFE_2338 | 2.32 |  | MutS2 family protein |
| AFE_2372 | 3.18 |  | type I restriction-modification system%2C M subunit |
| AFE_2374 | 4.63 | 5.23±0.40 | anticodon nuclease%2C putative |
| AFE_2380 | 2.83 |  | conserved domain protein |
| AFE_2387 | 2.15 |  | hydrolase%2C TatD family |
| AFE_2393 | 2.11 |  | ISAfe5%2C transposase orfA |
| AFE_2472 | 5.47 |  | hypothetical protein |
| AFE_2473 | 8.43 |  |  |
| AFE_2474 | 4.17 |  |  |
| AFE_2475 | 3.85 |  |  |
| AFE_2477 | 7.95 |  |  |
| AFE_2478 | 7.81 |  | hypothetical protein |
| AFE_2484 | 2.47 |  | hypothetical protein |
| AFE_2527 | 2.73 |  | membrane protein%2C DedA family |
| AFE_2528 | 2.22 |  | glutamine amidotransferase%2C class I |
| AFE_2575 | 2.03 |  | conserved domain protein |
| AFE_2583 | 2.12 |  | alpha-L-glutamate ligase%2C RimK family |
| AFE_2605 | 2.26 |  | ABC transporter%2C permease protein%2C putative |
| AFE_2610 | 2.47 |  | phenylalanyl-tRNA synthetase%2C alpha subunit |
| AFE_2618 | 2.59 |  | NADH-quinone oxidoreductase%2C M subunit |
| AFE_2621 | 2.59 |  | NADH-quinone oxidoreductase%2C J subunit |
| AFE_2623 | 2.38 |  | NADH-quinone oxidoreductase%2C H subunit |
| AFE_2628 | 2.14 |  | NADH-quinone oxidoreductase%2C C subunit |
| AFE_2629 | 2.05 |  | NADH-quinone oxidoreductase%2C B subunit |
| AFE_2643 | 4.35 |  | conserved hypothetical protein |
| AFE_2644 | 2.60 | 2.49±0.46 | metallo-beta-lactamase family protein |
| AFE_2682 | 6.00 |  | endonuclease III |
| AFE_2746 | 2.20 |  | tyrosine recombinase XerC |
| AFE_2747 | 2.57 |  | heat shock protein HslV |
| AFE_2748 | 3.04 |  | heat shock protein HslVU%2C ATPase subunit HslU |
| AFE_2819 | 2.19 |  | replicative DNA helicase |
| AFE_2826 | 3.47 | 1.63±0.08 | membrane protein%2C putative |
| AFE_2828 | 4.01 | 2.48±0.49 | 23S rRNA (uracil-5-)-methyltransferase |
| AFE_2841 | 2.08 |  | ROK family protein |
| AFE_2842 | 3.72 | 2.94±0.09 | glutamate racemase |
| AFE_2864 | 2.08 |  | ABC transporter%2C permease protein%2C putative |
| AFE_2925 | 3.34 |  | dolichyl-phosphate-mannose-protein mannosyltransferase family protein |
| AFE_2931 | 2.72 |  | exodeoxyribonuclease V%2C gamma subunit |
| AFE_2932 | 2.85 |  | exodeoxyribonuclease V%2C beta subunit |
| AFE_2933 | 2.20 |  | exodeoxyribonuclease V%2C alpha subunit |
| AFE_2934 | 3.35 | 1.80±0.17 | transcriptional regulator%2C putative |
| AFE_2935 | #### | 9.64±0.70 | TonB-dependent receptor |
| AFE_2936 | 2.75 |  | conserved hypothetical protein |
| AFE_2947 | 2.00 |  | thiol:disulfide interchange protein DsbG%2C putative |
| AFE_2954 | 2.33 |  | ribonuclease%2C Rne/Rng family |
| AFE_2960 | 3.49 | 2.88±0.30 | mannose-1-phosphate guanylyltransferase/mannose-6-phosphate isomerase |
| AFE_2961 | 4.73 | 2.38±0.17 | capsule polysaccharide export inner-membrane protein |
| AFE_2978 | 4.84 |  | conserved domain protein |
| AFE_2998 | 2.35 |  | TonB-dependent receptor |
| AFE_3013 | 2.26 |  | capsule polysaccharide modification protein |
| AFE_3015 | 2.90 |  | A/G-specific adenine glycosylase |
| AFE_3016 | 2.84 |  | capsular polysaccharide biosynthesis protein%2C putative |
| AFE_3046 | 3.11 |  | imidazoleglycerol phosphate synthase%2C cyclase subunit |
| AFE_3050 | 3.54 |  | Sec-independent protein translocase TatC |
| AFE_3072 | 5.14 |  | hypothetical protein |
| AFE_3089 | 2.00 |  | aspartate 1-decarboxylase |
| AFE_3109 | 2.02 | 1.94±0.15 | |
| AFE_3110 | 2.32 | 2.00±0.23 | |
| AFE_3111 | 2.54 | 1.53±0.15 | |
| AFE_3123 | 2.44 |  | adenylylsulfate reductase%2C thioredoxin dependent |
| AFE_3124 | 2.98 |  |  |
| AFE_3125 | 3.05 |  | sulfate adenylyltransferase%2C large subunit |
| AFE_3126 | 3.11 |  | uroporphyrin-III C-methyltransferase |
| AFE_3127 | 5.14 |  | conserved hypothetical protein |
| AFE_3128 | 7.34 |  | hypothetical protein |
| AFE_3135 | 2.02 |  | hypothetical protein |
| AFE_3137 | 2.31 |  | DNA-binding response regulator |
| AFE_3138 | 2.19 |  | oxidoreductase%2C 2OG-Fe(II) oxygenase family |
| AFE_3162 | 3.64 | 2.54±0.19 | acetyltransferase%2C GNAT family |
| AFE_3163 | 2.96 |  | isochorismate pyruvate lyase%2C putative |
| AFE_3164 | 4.42 | 2.28±0.17 | ABC transporter%2C ATP-binding protein |
| AFE_3165 | 4.11 | 2.48±0.13 | membrane protein%2C putative |
| AFE_3175 | 2.35 |  | hypothetical protein |
| AFE_3182 | 7.19 |  |  |
| AFE_3189 | 4.43 |  | conserved domain protein |
| AFE_3191 | 2.24 |  | type III restriction-modification system%2C Res subunit |
| AFE_3197 | 3.18 |  |  |
| AFE_3213 | 2.07 |  | methyltransferase GidB |
| AFE_3236 | 2.44 |  | ABC transporter%2C ATP-binding protein%2C NodI family |
| AFE_3267 | 3.35 |  | YaiI/YqxD family protein |
| AFE_3287 | 4.23 |  |  |
| AFE_3288 | 4.72 |  |  |
| AFE_3289 | 2.84 |  |  |
| AFE_3290 | 3.26 |  |  |
| AFE_3298 | 6.19 | 3.27±0.31 | delta-aminolevulinic acid dehydratase |
| AFE_3299 | 2.34 |  | uroporphyrinogen-III synthase |
| AFE_0105 | 0.28 | 0.24±0.05 | metal ion transporter%2C NRAMP family |
| AFE_0114 | 0.38 |  | lipase/esterase%2C putative |
| AFE_0124 | 0.49 |  | TPR domain protein |
| AFE_0127 | 0.41 |  | MiaB-like tRNA modifying enzyme YliG%2C putative |
| AFE_0137 | 0.33 |  | conserved hypothetical protein |
| AFE_0223 | 0.34 |  | UDP-3-0-acyl N-acetylglucosamine deacetylase |
| AFE_0256 | 0.49 |  | acetylglutamate kinase |
| AFE_0257 | 0.48 | 0.33±0.07 | secretion protein%2C HlyD family |
| AFE_0258 | 0.41 |  | hypothetical protein |
| AFE_0366 | 0.29 | 0.25±0.01 | glutathione reductase |
| AFE_0409 | 0.16 |  | conserved domain protein |
| AFE_0530 | 0.45 | 0.25±0.01 | carbohydrate kinase%2C PfkB family |
| AFE_0531 | 0.34 |  | conserved domain protein |
| AFE_0542 | 0.16 |  | chaperonin%2C 60 kDa |
| AFE_0543 | 0.22 |  | chaperonin%2C 10 kDa |
| AFE_0564 | 0.42 |  | DNA-directed RNA polymerase%2C omega subunit |
| AFE_0582 | 0.43 |  | conserved hypothetical protein |
| AFE_0594 | 0.47 |  | phosphatidylethanolamine N-methyltransferase%2C putative |
| AFE_0614 | 0.40 |  | conserved hypothetical protein |
| AFE_0615 | 0.40 | 0.33±0.03 | glycosyl transferase%2C group 1 |
| AFE_0616 | 0.43 | 0.34±0.02 | glycosyltransferase%2C group 1 |
| AFE_0622 | 0.44 |  | conserved hypothetical protein |
| AFE_0631 | 0.41 | 0.30±0.02 | cytochrome o ubiquinol oxidase%2C subunit II |
| AFE_0632 | 0.36 | 0.21±0.02 | cytochrome o ubiquinol oxidase%2C subunit I |
| AFE_0633 | 0.31 | 0.22±0.03 | cytochrome o ubiquinol oxidase%2C subunit III |
| AFE_0672 | 0.38 | 0.22±0.02 | iron-sulfur cluster assembly transcription factor IscR |
| AFE_0673 | 0.35 |  |  |
| AFE_0674 | 0.32 | 0.28±0.02 | FeS cluster assembly scaffold protein IscU |
| AFE_0675 | 0.36 | 0.35±0.01 | iron-sulfur cluster assembly protein IscA |
| AFE_0700 | 0.30 | 0.21±0.01 | hydrogenase sigma-54 dependent DNA-binding response regulator |
| AFE_0701 | 0.38 | 0.28±0.01 | hydrogenase-2%2C small subunit |
| AFE_0702 | 0.33 | 0.20±0.003 | hydrogenase-2%2C large subunit |
| AFE_0703 | 0.49 | 0.40±0.01 | conserved hypothetical protein |
| AFE_0705 | 0.47 | 0.39±0.01 | hydrogenase maturation protease |
| AFE_0707 | 0.12 | 0.10±0.01 | conserved hypothetical protein |
| AFE_0708 | 0.11 | 0.07±0.004 | hypothetical protein |
| AFE_0709 | 0.12 | 0.11±0.01 | membrane protein%2C putative |
| AFE_0710 | 0.19 | 0.08±0.01 | iron-sulfur cluster-binding protein%2C Rieske family |
| AFE_0711 | 0.22 | 0.14±0.01 | NHL repeat protein |
| AFE_0712 | 0.29 | 0.19±0.03 | [NiFe] hydrogenase maturation protein HypF |
| AFE_0713 | 0.32 | 0.23±0.01 | |
| AFE_0714 | 0.36 | 0.20±0.09 | phosphoheptose isomerase |
| AFE_0715 | 0.32 | 0.21±0.002 | |
| AFE_0716 | 0.34 | 0.27±0.003 | hydrogenase expression/formation protein HypE |
| AFE_0717 | 0.40 | 0.20±0.02 | |
| AFE_0718 | 0.40 | 0.26±0.03 | |
| AFE_0719 | 0.40 | 0.28±0.03 | amino acid permease family protein |
| AFE_0768 | 0.13 | 0.09±0.01 | MotA/TolQ/ExbB proton channel family protein |
| AFE_0774 | 0.45 |  | phosphorylase%2C putative |
| AFE_0852 | 0.49 |  | conserved hypothetical protein |
| AFE_0907 | 0.41 |  | orotidine 5`-phosphate decarboxylase |
| AFE_1440 | 0.25 |  | phosphate transport system protein PhoU |
| AFE_1527 | 0.41 |  | Nif-specific regulatory protein |
| AFE_1662 | 0.21 | 0.08±0.01 | NADH dehydrogenase%2C putative |
| AFE_1664 | 0.04 | 0.40±0.04 | glycolate oxidase%2C subunit GlcE |
| AFE_1669 | 0.22 |  | hypothetical protein |
| AFE_1670 | 0.10 |  | conserved hypothetical protein |
| AFE_1689 | 0.26 | 0.14±0.01 | Tat (twin-arginine translocation) pathway signal sequence domain protein |
| AFE_1690 | 0.22 |  |  |
| AFE_1691 | 0.24 |  |  |
| AFE_1779 | 0.47 |  | plasmid stability protein%2C putative |
| AFE_1785 | 0.49 |  | hypothetical protein |
| AFE_1792 | 0.34 | 0.25±0.03 | sulfide-quinone reductase%2C putative |
| AFE_1806 | 0.44 |  | hypothetical protein |
| AFE_1850 | 0.28 |  | conserved domain protein |
| AFE_1856 | 0.45 |  | dehydrogenase%2C homolog |
| AFE_1869 | 0.48 |  | cell division inhibitor%2C putative |
| AFE_1870 | 0.48 |  | conserved hypothetical protein |
| AFE_1939 | 0.49 |  |  |
| AFE_1951 | 0.48 |  | conserved hypothetical protein TIGR00481 |
| AFE_1955 | 0.49 |  | transcriptional regulator%2C Fur family |
| AFE_1968 | 0.43 |  | potassium-efflux system protein |
| AFE_1992 | 0.32 | 0.23±0.12 | C4-dicarboxylate transporter/malic acid transport protein |
| AFE_2023 | 0.50 |  | phosphoribosylglycinamide formyltransferase 2 |
| AFE_2077 | 0.36 |  | molybdopterin-guanine dinucleotide biosynthesis protein B |
| AFE_2109 | 0.44 |  |  |
| AFE_2193 | 0.40 |  | bis(5-nucleosyl)-tetraphosphatase (symmetrical) |
| AFE_2214 | 0.46 |  | conserved hypothetical protein |
| AFE_2255 | 0.40 |  | hypothetical protein |
| AFE_2256 | 0.45 |  | metallo-beta-lactamase family protein |
| AFE_2261 | 0.48 |  | phosphoribosylamine--glycine ligase |
| AFE_2271 | 0.29 | 0.19±0.01 | transcriptional regulator%2C putative |
| AFE_2278 | 0.08 | 0.08±0.01 | phosphonate metabolism protein PhnG |
| AFE_2316 | 0.33 | 0.26±0.03 | outer membrane lipoprotein Slp%2C putative |
| AFE_2342 | 0.30 | 0.17±0.01 | RNA polymerase sigm-70 factor family |
| AFE_2357 | 0.42 |  | lipoprotein%2C putative |
| AFE_2463 | 0.48 |  | hypothetical protein |
| AFE_2495 | 0.35 |  | hypothetical protein |
| AFE_2518 | 0.34 |  | hypothetical protein |
| AFE_2522 | 0.43 |  | carbohydrate-selective porin%2C OprB family |
| AFE_2524 | 0.48 | 0.27±0.04 | ferrous iron transport protein B |
| AFE_2542 | 0.35 | 0.20±0.02 | outer membrane protein%2C OMPP1/FadL/TodX family |
| AFE_2554 | 0.50 | 0.44±0.07 | heterodisulfide reductase subunit B%2C homolog |
| AFE_2567 | 0.50 |  | D-alanyl-D-alanine carboxypeptidase |
| AFE_2638 | 0.38 |  | ribonucleotide-diphosphate reductase%2C alpha subunit |
| AFE_2639 | 0.32 |  | ribonucleoside-diphosphate reductase%2C beta subunit |
| AFE_2667 | 0.28 |  | heat-inducible transcription repressor HrcA |
| AFE_2676 | 0.49 |  | hypothetical protein |
| AFE_2684 | 0.37 |  | hemagluttinin family protein |
| AFE_2687 | 0.40 |  | hypothetical protein |
| AFE_2718 | 0.46 |  | anaerobic ribonucleoside-triphosphate reductase%2C alpha subunit |
| AFE_2719 | 0.29 |  | conserved hypothetical protein |
| AFE_2785 | 0.29 |  | hypothetical protein |
| AFE_2907 | 0.41 |  | transglutaminase-like domain protein |
| AFE_2910 | 0.48 |  | conserved hypothetical protein |
| AFE_2958 | 0.45 |  | hypothetical protein |
| AFE_3038 | 0.49 |  | glutaredoxin-related protein |
| AFE_3075 | 0.43 |  | ribosomal protein L13 |
| AFE_3120 | 0.38 |  | membrane protein%2C putative |
| AFE_3152 | 0.41 | 0.32±0.04 | cytochrome c552 |
| AFE_3245 | 0.44 |  | anthranilate synthase component I |
| AFE_3281 | 0.49 |  | hydrogenase maturation protease |
| AFE_3307 | 0.50 |  | oxidoreductase%2C short-chain dehydrogenase/reductase family |

**Table S2 List of the primers used in this study.**

| **Primer name** | **Primer sequence (5'-3')** |
| --- | --- |
| Construction primers | |
| IUP-F | TTACGCGTCGACGTCTAGAGCGCAGCACTTCCACCT |
| IUP-R | TTTCGGGGTACCCTCGTCATCCTCATAGAGT |
| IDW-F | TTTCGGGGTACCCCGAAATTTCCGAATTCTG |
| IDW-R | GGAATTCCATATGAAGCTTTGATGCTGGGCTTGTGGTC |
| Ptac-F | TTTCCCAAGCTTGAATTCCGGCTCTAGACGACATCATAACGGTTCTG |
| Ptac-R | GAATTTGTTTCCTGTGTGAAATTG |
| PI-F | GCGGATAACAATTTCACACAGGAAACAAATTCATGCAGGTTATAACCGGG |
| PI-R | ATTTCTAGCTAGCCGGGATCCTTAGTCCAGATCTATCC |
| ACTUP-F | TTTGCTCTAGAATCGGACGGAGATTCGGAG |
| ACTUP-R | GGAATTCCATATGGCGTATCATGGAAAGCCGA |
| ACTDW-F | GGAATTCCATATGTCCCTCAACCAGCAGGCGCA |
| ACTDW-R | TCTAGCTAGCCGAATCTCTTCTGGCGTCC |
| Identification primers | |
| P1-F | TTACGCGTCGACGTCTAGAAACCCGAGCATCGCCGCCT |
| P1-R | GGAATTCCATATGAAGCTTTGTGGTGGGTATTCTCTGG |
| P2-F | TCCCACGAAGCAACATCA |
| P2-R | ACGGATAACCGCCAGA |
| P3-F | CCGAATAATCTGTATGCGA |
| P3-R | CGCTCGATGTGCGTGATAT |
| P4-F | TTTCCCAAGCTTGAATTCCGGCTCTAGACGACATCATAACGGTTCTG |
| P4-R | ATTTCTAGCTAGCCGGGATCCTTAGTCCAGATCTATCC |
| PA1-F | ATTGCTCTAGAAACCGTGTTGCCACAGCCA |
| PA1-R | CTAGCTAGCGAACAAACTGTACCCCCCA |
| PA2-F | CACCCTGACCGCTATCCACC |
| PA2-R | TACTGTTCGGCAGGGTCATT |
| PA3-F | TTCTATGGGGGCTTTACGAT |
| PA3-R | GAAACACCCGCTATTCACAG |
| RT-qPCR primers | |
| AFE_0042(tsd2)-F | TGGTCGTTCAAGCATCCG |
| AFE_0042(tsd2)-R | GCGTTCGCCTTCCATTTA |
| AFE_0043(sbp2)-F | GACAATCCGCAACTCACGA |
| AFE_0043(sbp2)-R | TGCCACCCTGCTTCATCTG |
| AFE_0044(doxDA2)F | CCACTGGATGGCCTACAAAT |
| AFE_0044(doxDA2)R | CCGACAATCAGCATAAAGCCTG |
| AFE_0045(rhd1)-F | TGTGCCAGGTGCTCAGAATA |
| AFE_0045(rhd1)-R | CAAGGCGTAGGCGTCATAAC |
| AFE_0046(rhd2)-F | GATGACGCACTGGTGGATAA |
| AFE_0046(rhd2)-R | GCATTCCGCAGCATCACA |
| AFE_0047(resA)-F | TCTCGTCCATCGGCTCCT |
| AFE_0047(resA)-R | GGCCCACGTGACTTATCCTT |
| AFE_0048(doxDA1)F | CCAGTTGTCCAGGGAATAAGC |
| AFE_0048(doxDA1)R | GCTTGTCCTTCGTGCTGATG |
| AFE_0049(sbp1)-F | GGGTGCTCCTTCTCAAACG |
| AFE_0049(sbp1)-R | AATCCAGCGACCTCCAAA |
| AFE_0050(tsd1)-F | TGTTCCAGATGAAATCGTCGTA |
| AFE_0050(tsd1)-R | AGTCCGTGGCTGAACTTGAT |
| AFE_2550(sdhC)-F | GGGAAATCCACGCTCACG |
| AFE_2550(sdhC)-R | CAACGGCCTGAAGATCGC |
| AFE_2551(hdrC)-F | GTTGCCCGGCACGTTTGA |
| AFE_2551(hdrC)-R | CGCCCACTGGATGGAAGA |
| AFE_2552(hyp)-F | GCGTTTCGTCTTTCCCGTAG |
| AFE_2552(hyp)-R | CGTGGAGCAGATGTTTACCG |
| AFE_2553(hdrA)-F | GCCAGATTCGTTGTCCTTC |
| AFE_2553(hdrA)-R | GGAAACGGTGCAACAGATC |
| AFE_2554(hdrB)-F | CGGATGTTGCTCATGTCGAT |
| AFE_2554(hdrB)-R | GCGGGAGAAGGTCAAGAAGA |
| AFE_2555(hdrC)-F | GTCCATCGCATCAATCCC |
| AFE_2555(hdrC)-R | AGGTCTGGTCGGTCTCGTC |
| AFE_2556(dsrE)-F | GTTATCCGCCCTTCATCCT |
| AFE_2556(dsrE)-R | CAGATGGCTCAAATCCTTC |
| AFE_2557(tusA)-F | CCGATTCTCCGCACCAA |
| AFE_2557(tusA)-R | GCTTCCGCCTGTTCCAA |
| AFE_2558(rhd)-F | ACAGGGAGGGGCGTTTA |
| AFE_2558(rhd)-R | CGAGCGGTTGCGATTTAT |
| AFE_2586(hdrB)-F | CAACTTCCATCAGGCCTAC |
| AFE_2586(hdrB)-R | TCTGGTGGACGGCAAGAT |
| AFE_0029(tetH)-F | TGTGGTCTATGCGGAAAG |
| AFE_0029(tetH)-R | GTTGGCGAAGTTGAAGGCT |
| AFE_0267(sqr)-F | ATACCTTCCGCAGCGAGTT |
| AFE_0267(sqr)-R | GGTAATGCAACAGATAACGCC |
| AFE_1792(sqr)-F | TGCCATGATCGTCGCTTCC |
| AFE_1792(sqr)-R | TTCCTTGAGGCCCTTCGTC |
| AFE_0269(sdo)-F | GAAACCAGCACCTACACCT |
| AFE_0269(sdo)-R | TGGGTATCGAGCACGTATG |
| AFE_2644(sdo)-F | ACATACCGATACCCACCACG |
| AFE_2644(sdo)-R | TTCCTGAACGGAGCGGTA |
| AFE_3136(regB)-F | AGTTATTACCGTTGCCACCCTT |
| AFE_3136(regB)-R | ATTCAAACCACATCCCCCATA |
| AFE_3137(regA)-F | ACGCCAGCATCGCAACAG |
| AFE_3137(regA)-R | ACAAGAACTCGTTTTTGACCG |
| AFE_3146(rus)-F | CGGGAATCTCCAAGGTCG |
| AFE_3146(rus)-R | GGAGAAAGACACCGGGAAA |
| AFE_3147(coxD)-F | TTCCTTGCCTGCCTCCTT |
| AFE_3147(coxD)-R | ATCGTTCGTTATCCCGTCC |
| AFE_3148(coxC)-F | GACCACCATCAGGGTTGCC |
| AFE_3148(coxC)-R | CGGTGCCCGCTACATTGAG |
| AFE_3149(coxA)-F | GAAACGCCAACCAGTCCA |
| AFE_3149(coxA)-R | GGCACCCCTTAGCGTATCT |
| AFE_3150(coxB)-F | CGTCCACTTCCATTGTTGCG |
| AFE_3150(coxB)-R | GACCTGGGCACCTTTCGTAG |
| AFE_3151(cup)-F | TGGGCACCACAAAACTGAAG |
| AFE_3151(cup)-R | TAGCCGTGGGATGGAAAGAT |
| AFE_3152(cyc1)-F | GGCACGGGAGTGGTCCTTAT |
| AFE_3152(cyc1)-R | GCGTTTCCAGCGATTGTATG |
| AFE_3153(cyc2)-F | GAGAAGCACCCGCATTAGAGT |
| AFE_3153(cyc2)-R | ACGGCAACAGAAACTCCAAGC |
| AFE_0954(cydB)-F | GGGTTGTCCGTGGAAGGC |
| AFE_0954(cydB)-R | TGCCCTTGCTGGGTTTGG |
| AFE_0955(cydA)-F | CGTGTTGTAATCCGTGTTCC |
| AFE_0955(cydA)-R | TATGGCGTTGCGTTCCTT |
| AFE_0631(cyoA)-F | ACCAACCATCTGAAACCCGTAA |
| AFE_0631(cyoA)-R | CGTGGTCACCGCAGAGGA |
| AFE_0632(cyoB)-F | TTTCTTCACGGCGGGTCT |
| AFE_0632(cyoB)-R | GCCAAATAATGGTTTTTCGGA |
| AFE_0633(cyoC)-F | GTGTATTGTTTCTGGCACTGGA |
| AFE_0633(cyoC)-R | CCATCATCACCAGAATCCAGA |
| AFE_0634(cyoD)-F | CGCCATTCGGTTTGCTGA |
| AFE_0634(cyoD)-R | TCCACCAGGTCAGTCCGAT |
| AFE_3107(cycA-1)-F | CCAGACTCCACCGAAAGCA |
| AFE_3107(cycA-1)-R | AAGCGAGGAAAGTAAGGCG |
| AFE_3108(sdr-1)-F | CGACGAGGAGTTGTGGGA |
| AFE_3108(sdr-1)-R | CGGGCGCAGTCATCAGTA |
| AFE_3109(petA1)-F | CCGCAGCAGCCTCCATAC |
| AFE_3109(petA1)-R | TGAAATCCGCCAAGCCAC |
| AFE_3110(petB1)-F | ATGGCGGAAGCCTTTGTC |
| AFE_3110(petB1)-R | TGTCGCAGACCCAATCCC |
| AFE_3111(petC1)-F | GGCAAGCCTCCTCCAAAC |
| AFE_3111(petC1)-R | TGGCAACCATTGGGAACA |
| AFE_2727(cycA2)-F | ACCCGCAATCACCCATCA |
| AFE_2727(cycA2)-R | GCAGACCACCGAAGTACAGCT |
| AFE_2728(sdr2)-F | CCAACACGCCTATTCGGGTA |
| AFE_2728(sdr2)-R | TCGCCGTTATGACCCAGA |
| AFE_2729(petA2)-F | CAGACGAAACGCAAGACAGC |
| AFE_2729(petA2)-R | GGCTGTTGGGTCCAGTGATT |
| AFE_2730(petB2)-F | TTTATCGGCGGTTGGGTTAC |
| AFE_2730(petB2)-R | TTGGAACCGACTTTATGGAGAT |
| AFE_2731(petC2)-F | CATAAATGGTTCGGGCTTCC |
| AFE_2731(petC2)-R | CCATAGGGGGCAAGCACG |
| AFE_2732(iro)-F | GGACGGTGGTCGGAGTAAA |
| AFE_2732(iro)-R | GCTACCACCTTACAGCACTTCG |
| AFE_1497(OprD)-F | GATGTCGGCAGGGCGTAGT |
| AFE_1497(OprD)-R | ATGGCGGACCCAATGTGC |
| AFE_1991(TonB)-F | TGGTGTAACCCAGAGTGCC |
| AFE_1991(TonB)-R | ACCCTGCTGAATTTCAACGA |
